# Analysis of East Asia Genetic Substructure Using Genome-Wide SNP Arrays

**DOI:** 10.1371/journal.pone.0003862

**Published:** 2008-12-05

**Authors:** Chao Tian, Roman Kosoy, Annette Lee, Michael Ransom, John W. Belmont, Peter K. Gregersen, Michael F. Seldin

**Affiliations:** 1 Rowe Program in Human Genetics, Departments of Biochemistry and Medicine, University of California Davis, Davis, California, United States of America; 2 The Robert S. Boas Center for Genomics and Human Genetics, Feinstein Institute for Medical Research, North Shore LIJ Health System, Manhasset, New York, United States of America; 3 Department of Molecular and Human Genetics, Baylor College of Medicine, Houston, Texas, United States of America; University of Montreal, Canada

## Abstract

Accounting for population genetic substructure is important in reducing type 1 errors in genetic studies of complex disease. As efforts to understand complex genetic disease are expanded to different continental populations the understanding of genetic substructure within these continents will be useful in design and execution of association tests. In this study, population differentiation (Fst) and Principal Components Analyses (PCA) are examined using >200 K genotypes from multiple populations of East Asian ancestry. The population groups included those from the Human Genome Diversity Panel [Cambodian, Yi, Daur, Mongolian, Lahu, Dai, Hezhen, Miaozu, Naxi, Oroqen, She, Tu, Tujia, Naxi, Xibo, and Yakut], HapMap [ Han Chinese (CHB) and Japanese (JPT)], and East Asian or East Asian American subjects of Vietnamese, Korean, Filipino and Chinese ancestry. Paired Fst (Wei and Cockerham) showed close relationships between CHB and several large East Asian population groups (CHB/Korean, 0.0019; CHB/JPT, 00651; CHB/Vietnamese, 0.0065) with larger separation with Filipino (CHB/Filipino, 0.014). Low levels of differentiation were also observed between Dai and Vietnamese (0.0045) and between Vietnamese and Cambodian (0.0062). Similarly, small Fst's were observed among different presumed Han Chinese populations originating in different regions of mainland of China and Taiwan (Fst's <0.0025 with CHB). For PCA, the first two PC's showed a pattern of relationships that closely followed the geographic distribution of the different East Asian populations. PCA showed substructure both between different East Asian groups and within the Han Chinese population. These studies have also identified a subset of East Asian substructure ancestry informative markers (EASTASAIMS) that may be useful for future complex genetic disease association studies in reducing type 1 errors and in identifying homogeneous groups that may increase the power of such studies.

## Introduction

Analysis of population genetic substructure has been enhanced by the ability to perform large genome array studies. The differences and patterns of variation within continental populations are useful for several reasons including recapitulating population migration and origins of ethnic groups, forensic identification, and for defining and applying an understanding of allele frequency variation to genetic association studies. Recent studies by several groups including our own have examined European population substructure [1]–[4]. Importantly, these studies have shown that discerning and accounting for differences in substructure can improve error rates in association studies [5]. With the availability of East Asian (EAS) SNP genotypes, we undertook the current study to perform similar studies for this sub-continental region that contains the largest contribution to the world's population. East Asian population genetic structure is particularly important since multiple genetic studies of complex disease are currently underway including studies of autoimmune diseases in Korean, Chinese and Japanese populations[6]–[11]. An understanding of the relationship among these different populations and ascertaining ancestry informative markers (AIMs) that can discern East Asian substructure will undoubtedly facilitate accurate interpretation of such studies[5].

This study combines high density SNP array genotypes from studies of EAS population groups within the Human Genome Diversity Panel (HGDP) [12], [13] with those of several additional population groups of EAS ancestry. The use of high density SNP genotypes containing over 200 K common autosomal genotypes allows a more comprehensive analyses than those previously performed using limited number of autosomal genotypes. It also complements studies of mitochondrial and Y chromosome haplogroups as well as classical markers that provide important information with respect to part of the history of particular EAS ethnic groups [14]–[20]. Our study expands on previous analyses using HGDP population groups [13] by examining additional parameters of population structure/diversity and by including many additional samples including those from several of the most populous EAS groups (Korean, Filipino and Vietnamese) and Chinese American participants of diverse origin. We apply the genotypic information to identify a set of SNPs that may be useful in the design and execution of association studies.

## Results

### Population Differentiation between East Asian Populations

**Figure 1:**
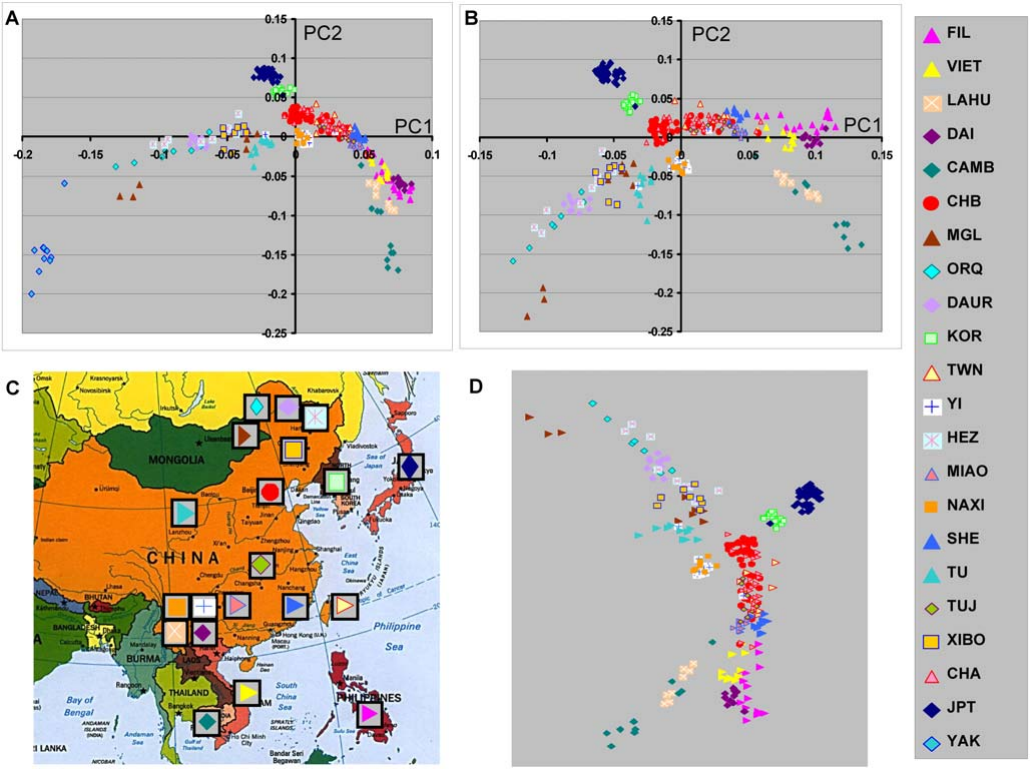
Principal component analyses of substructure in a diverse set of subjects of East Asian descent. Graphic representation of the first two PCs based on analysis with >200 K SNPs are shown. Color code shows subgroup of subjects for each population group. The subjects included Filipino (FIL), Vietnamese (VIET), Lahu, Dai, Cambodian (CAMB), Han Chinese (CHB), Mongola (MGL), Oroqen (ORQ), Daur, Korean (KOR), Chinese Americans from Taiwan (TWN),Yi, Hezhen (HEZ), Miaozu (MIAO), Naxi, She, Tu, Tujia (TUJ), Xibo, Chinese Americans (CHA), Japanese (JPT), and Yakut (YAK). A, Analyses including the Yakut population group. B, Analysis without Yakut is shown. C, Approximate geographic origin of population group is depicted on a map of East Asia (downloaded from University of Texas Library website). The positions of the HGDP population groups are based on the collection site information[12] and the other population groups were placed based on self-identified country or region of origin. [Note: Yakut are not shown on the map since this population is from Siberia and is a considerable distance north of the depicted region.] D, Shows rotated results of PC1 and PC2 to assist illustration of geographic correspondence of ethnic group locations.

To examine similarities and differences in population differentiation among EAS populations paired Fst values were determined between 19 EAS population groups that were typed with genome-wide SNP arrays (see **Methods**). The studies included samples derived from HapMap [21], [22], HGDP[13], samples collected in Korea and East Asian American participants (see **Methods**). The Fst values were obtained using three random non-overlapping sets of 3500 SNPs distributed over the autosomal genome (minimum of 50 kb distance between SNPs). This approach was taken to limit potential bias from SNPs in close linkage disequilibrium and to measure of variability of Fst. The small differences in these independent samplings (mean SD = 0.0015; median SD = 0.0013) indicate that this approach resulted in good estimations of paired Fst values. Relatively large Fst values were evident between many of the relatively small ethnic groups within China (**Table 1** **and see** **Figure 1** **for geographical information**). In particular, those population groups derived from Mongolia or near by provinces including Oroqen, Hezhen, and Daur show relatively large differences with Han Chinese. Similarly, two of the ethnic groups in the southeastern region of China, Lahu and Dai, also showed large paired Fst values with Han Chinese. With respect to population groups derived from very populous groups, the data indicate that Japanese and Korean were very closely related, as were Korean and Han Chinese but that these groups are much further from the south-east Asian populations (Filipino and Vietnamese). The Han Chinese and Japanese groups showed larger separation than either with Korean, although the paired Fst values were still small relative to Chinese/Filipino Fst. The Fst values also showed a close relationship between the Dai ethnic group in China and the Vietnamese population sample. Each of the groups had large paired Fst values with the Yakut from Siberia with the exception of the Mongolian, Hezhen and Oroqen ethnic groups that derive from north-eastern China or Mongolia. The relative size of the Fst values also generally corresponded to the geographical separation of the EAS population groups (depicted in **Figure 1**).

**Table 1:** Paired Fst values and Standard Deviations for EAS Population Groups^a^.

	CHB	KOR	FIL	VIET	CAMB	YAK	YI	DUAR	MGL	LAHU	DAI	HEZH	MIAO	NAXI	OROQ	SHE	TU	TUJ	XIB0
JPT^b^	0.0065+/−0.0003																		
KOR	0.0019+/−0.0002	0.0028+/−0.0003																	
FIL	0.0140+/−0.0007	0.0204+/−0.0004	0.0182+/−0.0015																
VIET	0.0065+/−0.0009	0.0146+/−0.0014	0.0106+/−0.0002	0.0112+/−0.0007															
CAMB	0.0136+/−0.0011	0.0210+/−0.0009	0.0191+/−0.0021	0.0158+/−0.0006	0.0062+/−0.0011														
YAK	0.0289+/−0.0009	0.0297+/−0.0020	0.0279+/−0.0010	0.0430+/−0.0002	0.0376+/−0.0008	0.0377+/−0.0023													
YI	0.0059+/−0.0008	0.0127+/−0.0002	0.0083+/−0.0006	0.0207+/−0.0015	0.0126+/−0.0012	0.0156+/−0.0021	0.0296+/−0.0016												
DUAR	0.0088+/−0.0023	0.0103+/−0.0016	0.0072+/−0.0023	0.0262+/−0.0017	0.0185+/−0.0028	0.0227+/−0.0017	0.0163+/−0.0010	0.0143+/−0.0021											
MGL	0.0049+/−0.0010	0.0082+/−0.0014	0.0052+/−0.0008	0.0212+/−0.0009	0.0139+/−0.0025	0.0161+/−0.0020	0.0141+/−0.0013	0.0085+/−0.0013	0.0081+/−0.0096										
LAHU	0.0223+/−0.0016	0.0295+/−0.0015	0.0271+/−0.0021	0.0300+/−0.0006	0.0219+/−0.0010	0.0237+/−0.0029	0.0511+/−0.0022	0.0261+/−0.0016	0.0346+/−0.0016	0.0280+/−0.0030									
DAI	0.0110+/−0.0004	0.0195+/−0.0007	0.0171+/−0.0023	0.0119+/−0.0020	0.0045+/−0.0008	0.0079+/−0.0020	0.0429+/−0.0015	0.0152+/−0.0018	0.0226+/−0.0006	0.0179+/−0.0016	0.0229+/−0.0016								
HEZH	0.0084+/−0.0011	0.0104+/−0.0016	0.0069+/−0.0010	0.0264+/−0.0009	0.0191+/−0.0001	0.0230+/−0.0009	0.0195+/−0.0032	0.0130+/−0.0010	0.0036+/−0.0007	0.0042+/−0.0005	0.0343+/−0.0037	0.0252+/−0.0016							
MIAO	0.0059+/−0.0010	0.0141+/−0.0003	0.0100+/−0.0001	0.0152+/−0.0010	0.0068+/−0.0008	0.0129+/−0.0005	0.0355+/−0.0019	0.0105+/−0.0008	0.0166+/−0.0017	0.0128+/−0.0017	0.0240+/−0.0032	0.0097+/−0.0014	0.0166+/−0.0004						
NAXI	0.0100+/−0.0014	0.0177+/−0.0021	0.0120+/−0.0010	0.0255+/−0.0025	0.0172+/−0.0004	0.0209+/−0.0008	0.0356+/−0.0023	0.0078+/−0.0013	0.0183+/−0.0030	0.0133+/−0.0033	0.0310+/−0.0007	0.0214+/−0.0016	0.0185+/−0.0010	0.0157+/−0.0019					
OROQ	0.0164+/−0.0011	0.0175+/−0.0003	0.0146+/−0.0007	0.0334+/−0.0027	0.0273+/−0.0038	0.0308+/−0.0021	0.0177+/−0.0006	0.0213+/−0.0014	0.0055+/−0.0014	0.0095+/−0.0008	0.0415+/−0.0010	0.0320+/−0.0028	0.0086+/−0.0013	0.0247+/−0.0028	0.0246+/−0.0032				
SHE	0.0090+/−0.0006	0.0168+/−0.0012	0.0123+/−0.0014	0.0187+/−0.0006	0.0110+/−0.0018	0.0181+/−0.0025	0.0418+/−0.0008	0.0159+/−0.0008	0.0202+/−0.0025	0.0160+/−0.0005	0.0303+/−0.0010	0.0148+/−0.0005	0.0220+/−0.0021	0.0117+/−0.0011	0.0211+/−0.0020	0.0293+/−0.0034			
TU	0.0035+/−0.0008	0.0093+/−0.0010	0.0055+/−0.0003	0.0191+/−0.0009	0.0120+/−0.0010	0.0145+/−0.0014	0.0243+/−0.0011	0.0043+/−0.0014	0.0078+/−0.0017	0.0045+/−0.0030	0.0261+/−0.0020	0.0163+/−0.0022	0.0101+/−0.0021	0.0086+/−0.0012	0.0098+/−0.0007	0.0173+/−0.0006	0.0145+/−0.0015		
TUJ	0.0003+/−0.0002	0.0083+/−0.0006	0.0038+/−0.0011	0.0120+/−0.0013	0.0037+/−0.0025	0.0122+/−0.0007	0.0318+/−0.0027	0.0054+/−0.0012	0.0113+/−0.0007	0.0065+/−0.0038	0.0195+/−0.0027	0.0076+/−0.0019	0.0119+/−0.0015	0.0034+/−0.0014	0.0087+/−0.0007	0.0196+/−0.0026	0.0076+/−0.0011	0.0032+/−0.0005	
XIB0	0.0034+/−0.0014	0.0076+/−0.0012	0.0033+/−0.0011	0.0197+/−0.0004	0.0120+/−0.0020	0.0167+/−0.0004	0.0185+/−0.0012	0.0096+/−0.0012	0.0045+/−0.0003	0.0017+/−0.0015	0.0286+/−0.0009	0.0182+/−0.0025	0.0052+/−0.0017	0.0114+/−0.0028	0.0139+/−0.0026	0.0099+/−0.0032	0.0148+/−0.0011	0.0035+/−0.0006	0.0055+/−0.0009

a Paired Fst values are the mean+/−S.D determined from three nonoverlapping sets of 3500 SNPs using the Weir and Cockerham algorithm (see **Methods**).

b The EAS population groups included Japanese from Tokyo (JPT) and Chinese from Behjing (CHB) both from HapMap data, Korean (KOR), Filipino (FIL), Vietnamese (VIET) from new typing studies and the following HGDP groups: Cambodian (CAMB), Yakut (YAK), Yi, Daur, Mongolian (MGL), Lahu, Dai, , Hezhen (HEZH), , Miaozu (MIAO), Naxi, Oroqen (OROQ), She, Tu, Tujia (TIJ), and Xibo. The geographic positions of these populations are provided in Figure 1 with the exception of the Yakut that derive from northern Siberia.

Fis values were also determined for each of the population sample and did not indicate a strong inbreeding component for any of the tested sample groups (**Supplemental Table S1**).

The different Chinese subjects derived from different regions of origin were also examined. For each of the Chinese American groups with self reported origin from North China, South China and Taiwan the paired Fst values with the Han Chinese from Beijing was small (<0.0025) (**Supplemental Table S2**).

### Principal Component Analyses Using >200 K SNPs Show Substructure Relationships

To further explore the relationship among EAS population groups and examine population substructure PCA was performed using the genotype results from a set of >200 K SNPs. Analyses were done with and without the inclusion of the Yakut population thought to originate in central Asia, since PCA results are influenced by the inclusion or exclusion of different population groups and we were interested in the relationship between EAS and central Asian populations. The first two principal components in these analyses display the largest genotype variation (**Table 2**) and are graphically depicted in **Figure 1**. Inclusion of the Yakut group showed a possible cline in PC1/PC2 that extends from the current Siberian location of the Yakut to the northern East Asian population groups (**Figure 1A**). Interestingly, the position of the different population groups shows a remarkable correspondence with the geographic origin of each group. This is more clearly suggested when the Yakut population is excluded (**Figure 1B**) and is best illustrated by comparing these geographic locations with rotated PCA results (**Figure 1C and D**). Additional, PCA analyses including the central Asian Uygur and Hazara population groups were also performed but these did not show a clear relationship with the EAS (Supplemental **Figure S1**).

**Table 2:** Evaluation of Principal Components Analyses in East Asian Populations using 200 K SNPs.

PC	All EAS Population Groups^a^			Five Population Groups		
	% Eigen^b^	SHT^c^	K-W Test^d^	% Eigen	SHT	K-W Test
1	17.9%	0.969+/−0.030	2.39E-41	18.0%	0.985+/−0.001	2.66E-26
2	12.0%	0.950+/−0.017	1.01E-40	12.0%	0.951+/−0.010	4.43E-24
3	10.6%	0.798+/−0.109	9.81E-39	9.2%	0.774+/−0.045	1.74E-14
4	10.0%	0.690+/−0.198	2.56E-36	8.8%	0.301+/−0.127	9.32E-01
5	8.9%	0.738+/−0.139	1.43E-31	8.8%	0.011+/−0.013	2.54E-01
6	8.4%	0.481+/−0.055	1.05E-28	8.7%	0.051+/−0.041	4.71E-02
7	8.2%	0.177+/−0.028	6.39E-23	8.7%	0.038+/−0.041	1.79E-01
8	8.0%	0.129+/−0.162	4.97E-10	8.6%	0.069+/−0.032	1.89E-01
9	8.0%	0.033+/−0.016	6.09E-05	8.6%	0.016+/−0.011	5.67E-01
10	7.9%	0.006+/−0.004	4.88E-02	8.6%	0.005+/−0.007	2.00E-01

PC	CHB, KOR, JPT			“Chinese” Groups Alone		
	% Eigen	SHT	K-W Test	% Eigen	SHT	K-W Test
1	15.2%	0.982+/−0.002	6.75E-22	11.5%	0.685+/−0.049	5.39E-06
2	9.8%	0.616+/−0.081	3.26E-08	10.0%	0.059+/−0.060	6.02E-01
3	9.5%	0.003+/−0.003	1.55E-01	10.0%	0.120+/−0.019	3.07E-01
4	9.5%	0.036+/−0.032	1.87E-01	9.9%	0.098+/−0.065	6.90E-01
5	9.5%	0.038+/−0.032	4.98E-01	9.9%	0.014+/−0.018	2.68E-01
6	9.3%	0.053+/−0.045	7.27E-01	9.8%	0.051+/−0.067	1.83E-01
7	9.3%	0.037+/−0.008	2.50E-02	9.8%	0.069+/−0.084	3.77E-01
8	9.3%	0.024+/−0.013	1.18E-01	9.7%	0.113+/−0.073	9.36E-01
9	9.3%	0.035+/−0.041	1.26E-01	9.7%	0.040+/−0.063	4.70E-01
10	9.3%	0.014+/−0.010	4.65E-02	9.7%	0.018+/−0.018	3.05E-01

a EAS population groups included each of the populations indicated in Figure 1.

b The % eigenvalue (Eigen) is the percentage of the total variance in the first ten PCs.

c The Spearman-Brown split half reliability test (SHT)[39] r^2^ is the mean+/−SD from the adjusted correlations between: 1) every other chromosomes; 2) half chromosomes (first half each chromosome and second half each chromosome); and 3) first half genome and second half genome (see **Methods**). These correlations were determined after PCA of each individual set.

d The Kruskal-Wallis test [23], a nonparametric alternative to the ANOVA was used to examine the statistical significance of the difference in PC scores among subject groups pre-assigned based on self-identification.

**Figure 2:**
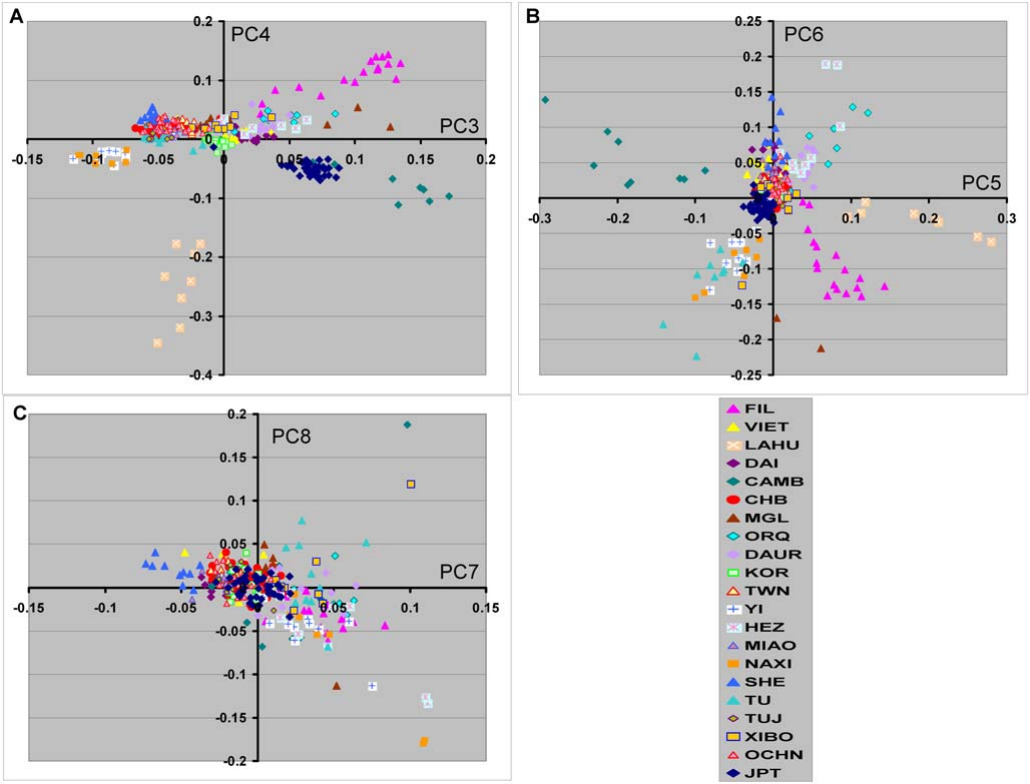
Graphic representation of additional principal components (PCs 3–8) in a diverse set of subjects of East Asian Descent. Color key shows groups as defined in Fig 1. A, PC3 and PC4. B, PC5 and PC6. C, PC7 and PC8.

The PCA results for PC1 and PC2 are generally consistent with the relative paired Fst values with respect to the distance separation among the different population groups. For example the position of the Korean group approximately midway between the HapMap CHB and JPT groups both graphically (**Figure 1**) and as discussed above for paired Fst values. It is also consistent with the closer relationship between the Dai ethnic group and the Vietnamese subjects. However, the first two PCs do not show the full relationships among the population groups. For example the Lahu ethnic group appears to be closely related to the Cambodian ethnic group (**Figure 1**), although the paired Fst value is relatively large (Table 1). Examination of additional PCs shows the large difference between the Lahu and Cambodian ethnic groups in PCs 3, 4 and 5 (**Figure 2**). Using both the Kruskal-Wallis test [23], a nonparametric alternative to the ANOVA, and a split half reliability test (see Methods) substructure was present in multiple principal components (**Table 2**). Substantial population substructure can be observed by the nonrandom grouping of population groups that extends through PC7.

**Figure 3:**
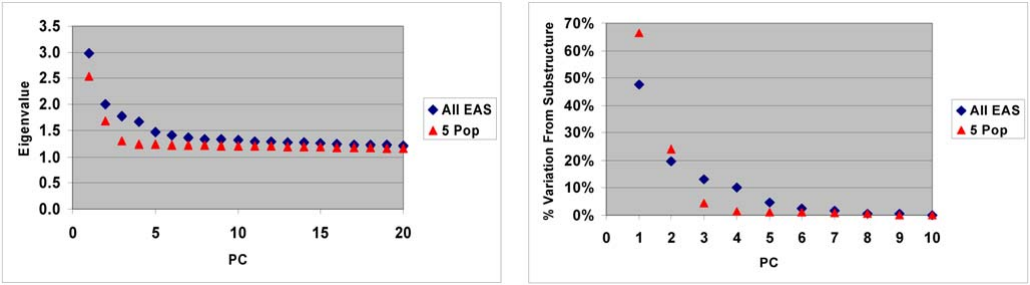
Eigenvalue distribution for principal components. A, The eigenvalues for each PC are shown for both the entire group of EAS (excluding Yakut), and for the five most populous ethnic groups (Chinese, Korean, Japanese, Filipino and Vietnamese). B, The proportion of the adjusted eigenvalue for each PC for the first 10 PCs is shown. For this measurement the PC10 eigenvalue for each group was used as the baseline. [Note: the eigenvalues plateau as shown in panel A and there is no discernable substructure beyond PC10 for these analyses (Table 2)]. For each PC, the PC10 eigen value was subtracted to determine an “adjusted” eigenvalue. The % substructure variation measurement was the proportion of each adjusted eigenvalue divided by the sum of the adjusted eigenvalues (PC1 through PC10).

For the entire EAS population groups studied, the majority of substructure variation defined by PCA appears to be within the first 4 PCs (**Table 2**). The eigenvalues plateau after PC4 with only small differences observed in subsequent PCs (**Figure 3a**). The proportion of the sum of the eigenvalues above this plateau provides a measure of the relative amount of substructure variation defined by each PC (**Figure 3b**). For the total EAS group, >90% of the substructure is defined in the first four PCs by this measurement. For the group of the five populations representing the most populous ethnic groups studied the first two PCs account for 90% of the variation above the plateau.

**Figure 4:**
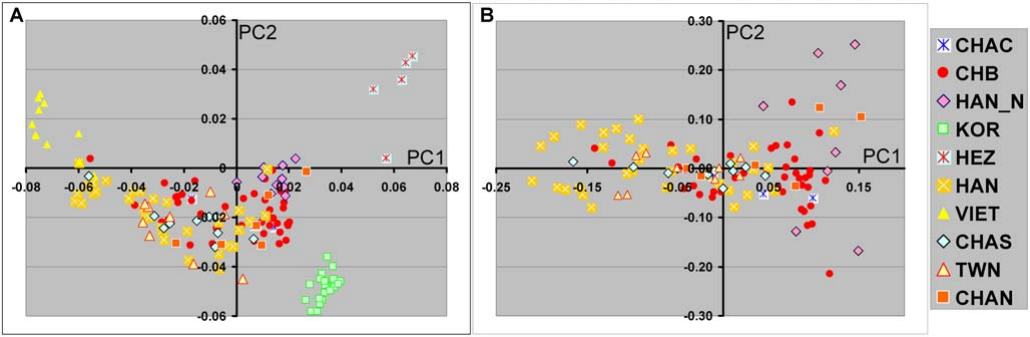
PCA analyses of Han Chinese and Chinese American population groups. A, Results from PCA performed together with EAS populations. B, PCA performed using only Chinese and Chinese American participants. The color coded population groups included the HapMap Han Chinese from Beijing (CHB), HGDP Han Chinese (HAN), HGDP North Han Chinese (HAN_N), Chinese American North (CHAN), Chinese American South (CHAS), Chinese American Central (CHAC), Taiwan Chinese American (TWN), Korean (KOR), and Hezhen (HEZ).

Similar analyses were also performed using population sets restricted to the more closely related Han Chinese, Japanese, and Korean groups, as well as a group restricted to Han Chinese and Chinese Americans (**Table 2**). These results as expected indicated substantially less substructure. However, even the subject set limited to Han Chinese and Chinese Americans showed substructure in PC1 using the split half reliability test and with the self identified groupings (ANOVA result). The relationship among the Han Chinese can be demonstrated in PCAs performed either including or excluding other EAS populations (**Figure 4**). Although there is variability in the distribution of many of the self-identified groups there was a general northwest/southeast gradient within these Chinese participants. In PC1 the North Han Chinese (HGDP from north central China[12]) were most separated from the southern Chinese participants including the Chinese American participants from Taiwan or with self-reported southern China origin.

### Informativeness of Smaller Sets of SNPs for Large East Asian Population Groups

We next examined the ability of smaller sets of SNPs to define population genetic structure in EAS populations. Random sets of 20 K, 5 K and 1 K SNPs were used to examine substructure in the combined population set and a subset of subjects from the most populous EAS groups (Han Chinese, Japanese, Korean, Filipino and Vietnamese). Correlation values (r^2^) were calculated comparing these SNP subsets with the 200 K SNP set. These results, summarized in Table 3, showed that the 20 K random SNP set and 5 K random SNP set corresponded closely with the >200 K SNP set for the first 4 PCs, with decreased correlations observed for the 1 K random SNP set. The relatively poor performance of the 1 K random sets was more pronounced when more closely related population groups were considered e.g. Japanese and Korean for PC1, 20 K/200 K r^2^ = 0.82+/−0.12 (mean+/−SD), 5 K/200 K r^2^ = 0.69+/−0.03, and 1 K/200 K r^2^ = 0.28+/−0.06. These results suggest that random sets of 5 K SNPs may be necessary for resolving and adjusting for substructure in these EAS populations (see discussion).

**Table 3:** Correlation of PCA Results using Random and Selected Sets with 200 K SNPs.

	All EAS Groups^a^			
	PC1	PC2	PC3	PC4
20 K random^b^	0.992+/−0.005	0.977+/−0.002	0.854+/−0.139	0.851+/−0.137
5 K random	0.956+/−0.005	0.888+/−0.021	0.725+/−0.086	0.705+/−0.081
1 K random	0.813+/−0.007	0.514+/−0.019	0.228+/−0.047	0.125+/−0.052

	Five Population Groups (CHB, JPT, KOR, FIL, VIET)			
	PC1	PC2	PC3	PC4
20 K random	0.991+/−0.002	0.961+/−0.005	0.897+/−0.073	0.754+/−0.198
5 K random	0.961+/−0.004	0.862+/−0.019	0.616+/−0.141	0.708+/−0.02
1 K random	0.814+/−0.024	0.419+/−0.074	0.192+/−0.165	0.218+/−0.074

	Test Population Group^c^	
	PC1	PC2
3 K random	0.862+/−0.035	0.446+/−0.096
3.0 K AIMs	0.953	0.848
1.5 K AIMs	0.939	0.819
750 AIMs	0.886	0.579

a Includes all EAS population groups (see Methods).

b Summary of analyses is provided for correlations of three independent random marker sets for each random marker group. For each random group the correlation with the full array set (>200 K SNPs) and is expressed as the mean r^2^+/−S.D.

c The tester population panel consisted of 20 Chinese, 20 Japanese, 4 Korean, 3 Filipino, 1 Vietnamese, 10 Dai and 10 Cambodian. . This test group did not contain any subjects used in the selection of the EAS-AIMs. As with other comparisons the correlation with the full array set (>200 K SNPs) is expressed as the mean r^2^+/−S.D. The EAS-AIMs are provided in Supplemental **Table S3**.

### East Asian Substructure Ancestry Informative Markers

**Figure 5:**
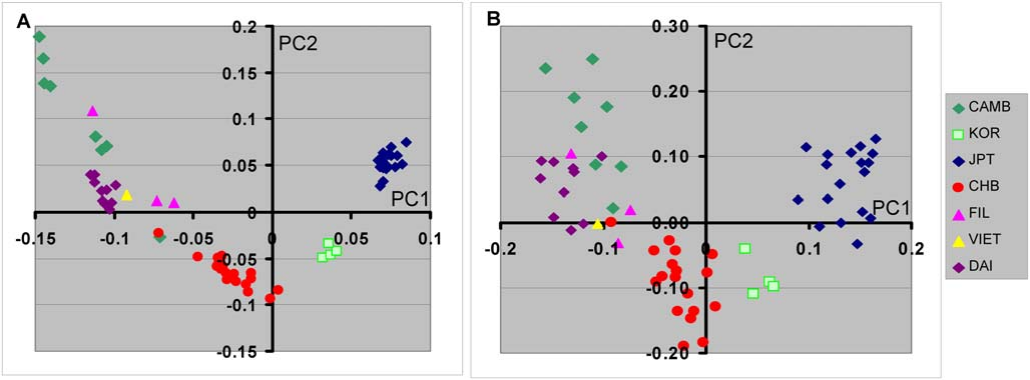
Ability of EAS-AIMs to discern population substructure. A, PCA analysis of tester population samples (see Table 3) using 200 K SNPs. B, PCA analysis of same tester population samples using 1500 EAS-AIMs.

AIMs that discern population substructure are likely to be useful in candidate gene, chromosomal position based association studies and defining homogeneous subject sets [24]. Since the application of these methods is most applicable to large population groups we restricted our ascertainment to five populations (Han Chinese, Japanese, Korean, Vietnamese and Filipino)(See **Methods**). To access the potential usefulness of these AIMs an independent set of samples was used and compared with the same number of random SNPs. For this assessment we included Cambodian and Dai samples since we had limited samples from the Vietnamese and Filipino populations. 3 K AIMs showed close correlation between the 200 K results for the first two PCs (Table 3). A set of the best 1.5 K AIMs also showed close correlation (**Figure 5** and **Table 3**). A reduced set of 750 AIMs showed a fall-off in correlation but was still equivalent to 3 K random SNPs. None of the AIM sets correlated with PC3 or PC4 (r2<0.01, p<0.05), however, these PCs distinguished the Dai and Cambodian from the other population groups and these were not included in our AIM selections. Nevertheless, for the common EAS populations these data suggest that the EAS-AIMs (**Table S3**) will be useful for association studies in the majority of EAS and EAS-American populations.

## Discussion

The current study extends the definition of EAS population substructure and the relationships among these ethnic groups. The inclusion of participant groups from populous countries in this region with large contributions to the USA population is an important aspect of our study. These population groups complement those included within HapMap studies as well as the HGDP in showing relationships between EAS groups and demonstrating that autosomal genotypes can be used to ascertain membership to various EAS groups. These results emphasize that EAS substructure, similar that previously shown for European substructure, will likely be important for complex disease association studies in defining study participants and reducing type 1 and type 2 error rates.

Our study extends the results of PCA analyses of EAS populations including those of HGDP populations that was recently reported [13]. The graphic representation of the first two PCs showed close correspondence to the historical geographical location and/or sample collection site for most of the EAS population groups. Thus, despite admixture and perhaps uncertain migration patterns, overall the largest component of genotypic variation that is discernable by reducing high order data (all genotypes) to lower order variations (PCs) is consistent with the population geography. This finding supports hypotheses that the relationships among the EAS populations are largely explained by clines formed by demic expansion(s). We speculate that the inclusion of many different related ethnic groups has recapitulated the most common events that separated these ethnic groups. The first PC axis accounting for the largest variation has a north/south orientation. One major part of this pattern forms a line from Siberia (Yakut) to Mongolia to Eastern China (**Figure 1**). The PCA analyses also suggest that at least two separate clines originating or terminating in eastern China at one end and Cambodia and the Philippines at the other end. In addition there is another cline extending from Eastern China to the Korean peninsular and Japan.

Multiple previous studies have examined the relationship between and possible origins of different EAS population groups. Analysis of mitochondrial and Y chromosome haplogroups as well as a limited numbers of classical markers and microsatellite polymorphisms have also provided results that are generally consistent with a north/south orientation of relationships between different EAS population groups [15]–[18]. However, there are exceptions with some studies failing to show this relationship [19], [25]. Summarized by a recent review [26] there are three different postulates regarding the origins of EAS population groups: 1) South East Asian origin [14]–[18], 2) North Asian origin [27] and 3) a combination of northern and southern origin [19], [20]. However, the majority of studies have supported a South-East Asian origin for most EAS populations and include detailed analyses of the age of specific mitochondrial haplogroups, Y chromosome sequences as well as limited marker studies [26]. In contrast, hierarchical trees in the recent HGDP study [13] show branching points consistent with a Yakut derivation. Recent studies using a novel copying model statistical approach appear to suggest an initial northern and southern origin (Cambodians, Mongolians, Xibo, Yi , Tu, Daur, and Naxi receiving large contributions from central-Asian populations) that contribute to Han ancestry [28]. These studies also provide data supporting the derivation of many other EAS groups from a Han expansion (including She, Japanese, Dai, Lahu and Miao). While the current study does not strongly support any of these hypotheses, it does suggest that eastern China is central to the events shaping the population groups in this region.

Multiple additional PCs are necessary to define the overall substructure relationships for the entire group of EAS populations studied as shown in **Figure 2**. However, most of the variation is discerned in the first four PCs for the EAS populations examined and in the first two PCs for the five most populous EAS groups studied. There was no geographic correspondence of the additional PCs and it is unclear whether these additional patterns correspond to individual or multiple different events in the histories of these population groups. Overall the size of the paired Fst values, as expected, showed a strong correlation with the PC eigenvalues summed over the first four PCs (data not shown). Although Fis values do not provide evidence for inbreeding in the current populations, it is unclear whether inbreeding or other factors including bottlenecks during the history of particular EAS ethnic groups may have contributed to the relationships between these populations.

An important aspect of the current study was the identification of EAS-AIM sets. The results show that these AIMs can distinguish the major variation between the populous population groups including Han Chinese, Japanese, Korean, Vietnamese, and Filipino. Additional testing to examine correction for stratification with these population groups was not possible due to limited genotypes currently available. However, by analogy with previous studies in European population groups, these AIMs particular the 1500 EAS-AIM set should be effective in addressing population stratification. The close correspondences of the relative positions in the first two PCs in individual subjects, even within the Han Chinese group, support the potential use of these SNP AIMs. Furthermore, the SHT analysis suggests that studies within the Han Chinese population and Chinese-Americans will benefit from the use of such AIMs in candidate gene studies.

## Methods

### Populations studied

The populations including those from the HGDP, HapMap, the I-control database, a Korean sample set and East Asian Americans. For all but the East Asian American and Korean samples set, genotypes were available from online databases. These included HapMap subjects (44 CHB and 44 JPT) and HGDP subjects (10 Cambodian, 10 Dai, 24 Hazara, 9 Hezhen, 27 Japanese, 10 Miaozu, 7 Naxi, 8 Oroqen, 10 She, 10 Tu, 10 Tujia, 8 Xibo, 13 Yakut and 44 Han Chinese) from the I-ControlDB (www.illumina.com/iControlDB, Illumina, San Diego, CA). Genotypes from other HGDP subjects (10 Daur, 8 Lahu, 9 Mongola, 10 Uygur, 10 Yi,) were from the NIH Laboratory of Neurogenetics (http://neurogenetics.nia.nih.gov/paperdata/public/).

For all EAS American and Korean subjects, blood cell samples were obtained from all individuals, according to protocols and informed-consent procedures approved by institutional review boards, and were labeled with an anonymous code number linked only to demographic information.

The Korean participants were from recruited in Korea (21 subjects). The EAS American samples were individuals born in the respective EAS country and were from Vietnam (22 subjects), Philippines (17 subjects) and different regions of the Peoples Republic of China (23 subjects) and Taiwan (9 subjects). The Filipino American participants included 15 that were recruited as part of the New York Cancer Project (NYCP); a prospective longitudinal study [29] and two recruited in Houston TX. 3 Filipino, 15 Vietnamese and 32 Chinese American samples were recruited in Houston TX. An additional 7 Vietnamese and 3 Korean genotypes were from the I-ControlDB. Of the Chinese American participants (CHA), 28 also indicated their general origin from regions within China (6 north, 10 south, 3 central and 9 subjects Taiwan).

### Genotyping

Genotyping was performed using a 300 K Illumina array according to the Illumina Infinium 2 assay manual (Illumina, San Diego), as previously described [30].

### Data Filters

SNPs and individual samples with less than 90% complete genotyping information from any data set were excluded from analyses. SNPs that showed extreme deviation from Hardy Weinberg equilibrium (p<0.00001) in individual population groups were also excluded from analysis. These filters resulted in a total of 215 K autosomal SNPs that were used for these studies. In addition, for samples from nonHGDP origin individuals with evidence of >10% contribution from other continents were excluded from further study. This was either performed prior to Illumina array genotyping for the Filipino, Vietnamese and CHA subjects using 128 continental AIMS [31]. Samples were also filtered for possible cryptic relationships using the PLINK program [32].

### Statistical Analyses

F_st_ and F_is_ was determined using Genetix software[33] that applies the Weir and Cockerham algorithm[34]. A measure of informativeness for each SNP (I_n_) was determined using an algorithm previously described [35]. Hardy-Weinberg equilibrium was determined using HelixTree 5.0.2 software (Golden Helix, Bozeman, MT, USA).

Population structure was examined using STRUCTURE v2.1[36], [37] using parameters and AIMs previously described [31]. This analysis was performed to exclude individuals with evidence of substantial continental admixture from Europe, Africa or the American continent (see Data Filters).

PCA was performed using the EIGENSTRAT statistical package[38]. All analyses were performed after deleting the MHC region on chromosome 6 since regions of high linkage disequilibrium can overly influence PCA results. The Kruskal-Wallis test [23], a nonparametric alternative to the ANOVA was used to examine the statistical significance of the difference in PC scores among subject groups pre-assigned based on self-identification.

The split half reliability test can determine whether independent (non-overlapping) SNP sets provide the same or different results. The split half reliability test was adjusted by the Spearman-Brown formula [39] and was performed three times using 1) alternate chromosomes, 2) alternate half chromosomes, and 3) half genome SNP sets. These sets were chosen to eliminate any dependency in each test between the two half data sets based on linkage disequilibrium.

### Selection of EAS-AIMs

Genotypes from 32 Han Chinese (CHA and CHB), 36 Japanese (JPT), 19 Korean, 21 Filipino and 14 Vietnamese were used for SNP selection. An initial set of 3000 EAS substructure AIMs (EAS-AIMs) were based on either I_n_ values or using SNP scores from PCA. The best performance using a testing panel was observed using a set of SNPs selected using I_n_ values from a combination of 1) all five population groups (top 600 SNPs), 2) Chinese and Japanese (top 1200 SNPs), and 3) Chinese and Filipino (top 1200 SNPs). The best performance of a 1500 SNP set and a 750 SNP set were observed using a combination of 500 or 250 from each of these three groups. The testing panel consisted of 20 Chinese, 20 Japanese, 4 Korean, 3 Filipino, 1 Vietnamese, 10 Dai and 10 Cambodian. None of the samples in the testing panel overlapped with the ascertainment samples. The Dai and Cambodian samples were included since there were limited numbers of samples available from the Vietnamese group. The performance of the EAS-AIMs was evaluated using correlations in PC1 and PC2 with the >200 K SNP set.

## Supporting Information

Table S1 Fis Values for East Asian populations
(0.06 MB DOC)

Table S2 Paired Fst Values for Chinese-Americans of Different Geographic Origin and CHB.
(0.05 MB DOC)

Table S3 List of 3K East Asian Ancestry Informative Markers
(0.45 MB DOC)

Figure S1 Principal component analyses of relationship between Central Asian and East Asian population groups. Both panels show graphic representation of the first two PCs genotyped with >200K SNPs A, East Asian population plus Uygur (UYG). B, East Asian population groups plus Hazara (HAZ). Color code shows subgroup of subjects for each population group. The subjects included Filipino (FIL), Vietnamese (VIET), Lahu, Dai, Cambodian (CAMB), Han Chinese (CHB), Mongola (MGL), Oroqen (ORQ), Daur, Korean (KOR), Chinese Americans from Taiwan (TWN),Yi, Hezhen (HEZ), Miaozu (MIAO), Naxi, She, Tu, Tujia (TUJ), Xibo, Chinese Americans (CHA), Japanese (JPT), and Yakut (YAK).
(1.65 MB TIF)
