# Supplementary material for: Analysis of East Asia Genetic Substructure Using Genome-Wide SNP Arrays
Source: PLoS One. 2008 Dec 5;3(12):e3862. doi: 10.1371/journal.pone.0003862 (PMC2587696; doi:10.1371/journal.pone.0003862)
Supplement: Table S1 — Fis Values for East Asian populations (0.06 MB DOC) [file pone.0003862.s001.doc]

**Table S1. Fis Values for East Asian populations^a^**

|  | Mean | S.D. |
| --- | --- | --- |
| CHB^b^ | 0.0028 | 0.0010 |
| JPT | 0.0055 | 0.0031 |
| KOR | 0.0105 | 0.0032 |
| FIL | -0.0146 | 0.0217 |
| VIET | 0.0037 | 0.0057 |
| CAMB | 0.0012 | 0.0077 |
| YAKUT | 0.0068 | 0.0012 |
| YI | 0.0037 | 0.0024 |
| DAUR | 0.0006 | 0.0041 |
| MONG | 0.0055 | 0.0053 |
| Lahu | -0.0057 | 0.0078 |
| DAI | 0.0097 | 0.0059 |
| HEZH | -0.0002 | 0.0052 |
| MIA | 0.0013 | 0.0049 |
| NAXI | -0.0013 | 0.0140 |
| OROQ | -0.0042 | 0.0067 |
| SHE | -0.0024 | 0.0090 |
| TU | -0.0076 | 0.0023 |
| TUIJA | 0.0114 | 0.0011 |
| XIBI | -0.0002 | 0.0032 |

a. Fis values are the mean +/- S.D determined from three non-overlapping sets of 3500 SNPs using the Weir and Cockerham algorithm (see Methods).

b. The EAS population groups included Japanese from Tokyo (JPT) and Chinese from Behjing (CHB) both from HapMap data, Korean (KOR), Filipino (FIL), Vietnamese (VIET) from new typing studies and the following HGDP groups: Mongolian (MONG), Lahu, Dai, Yakut (YAK) and Hezhen (HEZH). The central Asian HGDP Yakut (YAK) are included for comparison
