# Supplementary material for: Analysis of East Asia Genetic Substructure Using Genome-Wide SNP Arrays
Source: PLoS One. 2008 Dec 5;3(12):e3862. doi: 10.1371/journal.pone.0003862 (PMC2587696; doi:10.1371/journal.pone.0003862)
Supplement: Table S2 — Paired Fst Values for Chinese-Americans of Different Geographic Origin and CHB. (0.05 MB DOC) [file pone.0003862.s002.doc]

**Table S2. Paired Fst values among Chinese populations^a^**

|  | CHB | TWN | CHAN | CHAS | HAN |
| --- | --- | --- | --- | --- | --- |
| TWN | 0.0015 +/- 0.0002 |  |  |  |  |
| CHAN | -0.0024 +/- 0.0013 | 0.0032 +/- 0.0017 |  |  |  |
| CHAS | -0.0006 +/- 0.0003 | 0.0026 +/- 0.0044 | -0.0017 +/- 0.0015 |  |  |
| HAN | 0.0021 +/- 0.0012 | 0.0025 +/- 0.0011 | 0.0008 +/- 0.0013 | 0.0014 +/- 0.0010 |  |
| HAN_N | 0.0019 +/- 0.0020 | 0.0047 +/- 0.0016 | 0.0008 +/- 0.0018 | 0.0026 +/- 0.0014 | 0.0026 +/- 0.0003 |

a. Fst values are the mean +/- S.D determined from three nonoverlapping sets of 3500 SNPs using the Weir and Cockerham algorithm (see Methods).

b. The population groups included the HapMap Han Chinese from Beijing (CHB, 43 subjects), HGDP Han Chinese (HAN, 34 subjects), HGDP North Han Chinese (HAN_N, 10 subjects), Chinese American North (CHAN, 6 subjects), Chinese American South (CHAS, 10 subjects), Chinese American Central (CHAC, 3 subjects), and Taiwan Chinese American (TWN, 9 subjects).
