# Supplementary figures and images for: Analysis of East Asia Genetic Substructure Using Genome-Wide SNP Arrays
Source: PLoS One. 2008 Dec 5;3(12):e3862. doi: 10.1371/journal.pone.0003862 (PMC2587696; doi:10.1371/journal.pone.0003862)

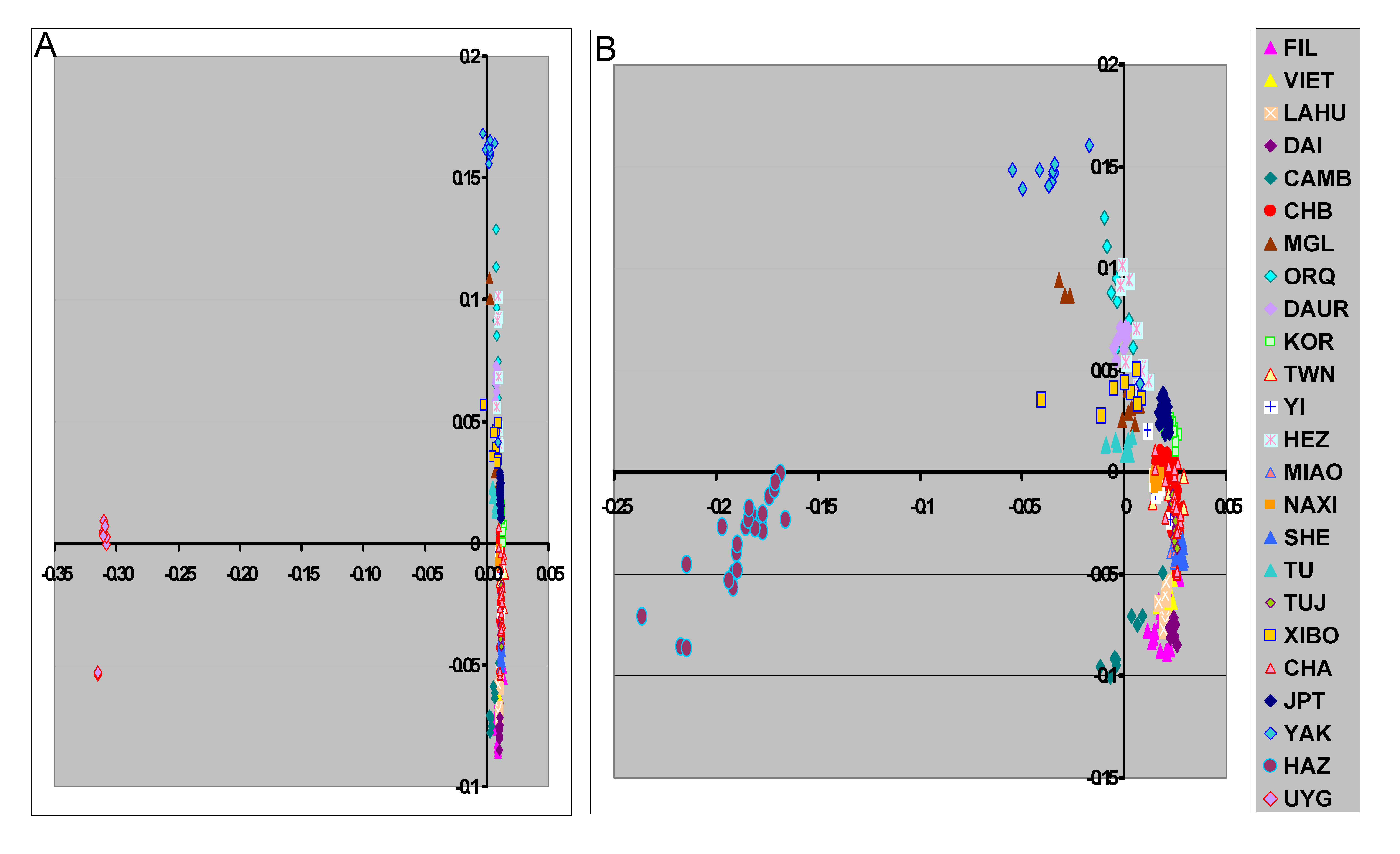

Supplement: Figure S1 — Principal component analyses of relationship between Central Asian and East Asian population groups. Both panels show graphic representation of the first two PCs genotyped with >200K SNPs A, East Asian population plus Uygur (UYG). B, East Asian population groups plus Hazara (HAZ). Color code shows subgroup of subjects for each population group. The subjects included Filipino (FIL), Vietnamese (VIET), Lahu, Dai, Cambodian (CAMB), Han Chinese (CHB), Mongola (MGL), Oroqen (ORQ), Daur, Korean (KOR), Chinese Americans from Taiwan (TWN),Yi, Hezhen (HEZ), Miaozu (MIAO), Naxi, She, Tu, Tujia (TUJ), Xibo, Chinese Americans (CHA), Japanese (JPT), and Yakut (YAK). (1.65 MB TIF) [file pone.0003862.s004.tif]
