# Supplementary material for: Analysis of East Asia Genetic Substructure Using Genome-Wide SNP Arrays
Source: PLoS One. 2008 Dec 5;3(12):e3862. doi: 10.1371/journal.pone.0003862 (PMC2587696; doi:10.1371/journal.pone.0003862)
Supplement: Supplementary file 4 [file pone.0003862.s003.doc]

Table S3. List of 3K East Asian Ancestry Informative Markers

rs number	Chr	bp	I_n 5 pop[a]	I_n CHN/FIL	I_n CHN/JPN	1.5K EAS-AIMs[b]	0.75K
rs3795263	1	2962146	0.030	0.000	0.046	1	
rs4654461	1	4805173	0.041	0.096	0.008	1	1
rs2898853	1	4902577	0.023	0.003	0.040		
rs770702	1	5679187	0.025	0.051	0.001		
rs1894647	1	7140216	0.017	0.004	0.036		
rs10864291	1	7261013	0.026	0.049	0.000		
rs11121129	1	8202361	0.025	0.051	0.005		
rs6667629	1	10458733	0.063	0.017	0.000	1	1
rs17037122	1	11623929	0.028	0.036	0.047	1	
rs1801133	1	11790644	0.071	0.101	0.021	1	1
rs2697983	1	13902883	0.024	0.001	0.033		
rs12024171	1	14312997	0.028	0.052	0.000		
rs1737352	1	14865832	0.038	0.042	0.000		
rs3738814	1	17076982	0.021	0.046	0.004		
rs1578215	1	17864907	0.043	0.076	0.022	1	1
rs2743189	1	18801688	0.032	0.001	0.049	1	
rs4278387	1	19596322	0.020	0.000	0.033		
rs1473689	1	19825616	0.046	0.080	0.006	1	1
rs212531	1	21333584	0.038	0.060	0.012	1	
rs885814	1	21621222	0.065	0.016	0.044	1	1
rs7524102	1	22443753	0.058	0.001	0.001	1	
rs2869511	1	22886006	0.048	0.006	0.068	1	1
rs10799802	1	23754850	0.032	0.031	0.033		
rs2076346	1	23828955	0.062	0.131	0.019	1	
rs4564081	1	24339214	0.051	0.126	0.032		
rs2248219	1	24390662	0.104	0.205	0.024	1	1
rs419655	1	25058268	0.026	0.045	0.000		
rs4568778	1	25143273	0.056	0.001	0.048	1	
rs4233461	1	26365448	0.027	0.044	0.012		
rs11247653	1	27673762	0.043	0.047	0.003		
rs4256835	1	27960531	0.019	0.005	0.036		
rs508448	1	29002141	0.025	0.000	0.041		
rs1321117	1	30296712	0.017	0.026	0.032		
rs4949444	1	31691124	0.028	0.000	0.049	1	
rs12564600	1	33580740	0.042	0.080	0.004	1	1
rs6697504	1	34056957	0.022	0.025	0.041		
rs1342476	1	34721274	0.055	0.017	0.006	1	
rs7548924	1	34764461	0.020	0.032	0.032		
rs4320749	1	34830895	0.033	0.054	0.000		
rs2745481	1	37535436	0.060	0.051	0.011		
rs4653309	1	37773433	0.067	0.015	0.015	1	1
rs10908366	1	37791027	0.041	0.021	0.044	1	
rs2147840	1	38891625	0.063	0.056	0.000	1	1
rs704782	1	39853826	0.031	0.004	0.041		
rs7520394	1	40778962	0.040	0.067	0.053	1	1
rs928508	1	40892507	0.070	0.170	0.036	1	1
rs883062	1	42278960	0.026	0.060	0.023	1	
rs841852	1	43070592	0.067	0.060	0.000	1	1

rs669446	1	43798872	0.025	0.001	0.040		
rs6693689	1	44180502	0.091	0.139	0.003	1	1
rs7525212	1	44698772	0.058	0.077	0.000	1	
rs6429566	1	45610945	0.018	0.042	0.011		
rs2236560	1	46207454	0.036	0.062	0.002	1	
rs10489770	1	46519617	0.054	0.015	0.040		
rs1440487	1	46879095	0.032	0.043	0.000		
rs505399	1	47648716	0.034	0.051	0.000		
rs875398	1	47987283	0.024	0.006	0.035		
rs11205541	1	48966436	0.027	0.054	0.001		
rs1769299	1	53297744	0.021	0.048	0.007		
rs1288617	1	53664162	0.026	0.000	0.032		
rs6661652	1	55488720	0.029	0.056	0.003		
rs4367814	1	55981842	0.031	0.002	0.042	1	
rs12081589	1	56693646	0.032	0.000	0.047	1	
rs12132541	1	58434484	0.040	0.048	0.000		
rs2760494	1	58950391	0.057	0.085	0.000	1	1
rs6677752	1	59118045	0.063	0.028	0.019	1	1
rs2897708	1	59913216	0.045	0.065	0.002	1	
rs636154	1	60290795	0.063	0.002	0.006	1	1
rs10515373	1	60413745	0.033	0.058	0.000	1	
rs17303103	1	61016586	0.023	0.045	0.000		
rs1572312	1	61129750	0.028	0.025	0.042	1	
rs2207792	1	61606730	0.033	0.064	0.002	1	
rs10493310	1	61843828	0.035	0.011	0.038		
rs825191	1	64420032	0.027	0.049	0.035		
rs310247	1	65019430	0.022	0.042	0.009		
rs4567260	1	66665503	0.020	0.004	0.036		
rs11209216	1	68283452	0.034	0.063	0.000	1	
rs2033348	1	68286950	0.028	0.057	0.042	1	
rs1021737	1	70616821	0.023	0.044	0.001		
rs602383	1	71103667	0.065	0.008	0.018	1	1
rs2639336	1	71388741	0.026	0.018	0.034		
rs6695355	1	71474224	0.026	0.062	0.011	1	
rs696699	1	74780082	0.078	0.004	0.007	1	1
rs12096276	1	75014468	0.058	0.057	0.003		
rs942699	1	76532456	0.036	0.005	0.059	1	1
rs2103119	1	79909593	0.035	0.043	0.018		
rs6424687	1	80254099	0.059	0.012	0.005	1	
rs1896176	1	81189080	0.021	0.051	0.005		
rs11163183	1	81190058	0.021	0.001	0.042	1	
rs4364877	1	82980363	0.029	0.001	0.047	1	
rs3861699	1	84243138	0.036	0.075	0.012	1	1
rs12727784	1	85980053	0.021	0.034	0.037		
rs2791473	1	86700730	0.054	0.021	0.000		
rs1321681	1	86717423	0.049	0.065	0.000	1	
rs11161993	1	87550629	0.019	0.024	0.032		
rs305217	1	88932493	0.057	0.038	0.009	1	
rs12732279	1	89537341	0.021	0.008	0.032		
rs2212940	1	90815248	0.018	0.027	0.037		
rs6696232	1	91268678	0.026	0.045	0.000		

rs2296620	1	91886584	0.027	0.008	0.042	1	
rs6677523	1	91914488	0.040	0.063	0.001	1	
rs236301	1	93826002	0.050	0.122	0.052	1	1
rs472908	1	94199375	0.054	0.000	0.047		
rs4847303	1	94602933	0.042	0.080	0.003	1	1
rs2039614	1	95402290	0.027	0.066	0.019	1	
rs4949960	1	95493238	0.022	0.020	0.045	1	
rs11804383	1	97223504	0.026	0.000	0.051	1	1
rs822607	1	98442946	0.055	0.000	0.002		
rs10875145	1	98555392	0.030	0.048	0.012		
rs4908314	1	99512152	0.029	0.047	0.000		
rs12129732	1	100921674	0.033	0.051	0.008		
rs10493940	1	101089866	0.037	0.009	0.039		
rs952484	1	102429031	0.063	0.052	0.001	1	1
rs7524694	1	103731652	0.033	0.047	0.004		
rs1930212	1	104036840	0.066	0.021	0.022	1	1
rs1928329	1	104478200	0.025	0.047	0.008		
rs11184189	1	104753516	0.019	0.035	0.035		
rs1444038	1	107693358	0.025	0.052	0.040		
rs1332684	1	108126272	0.063	0.125	0.004	1	1
rs484959	1	110078125	0.027	0.041	0.014		
rs975230	1	111053199	0.040	0.075	0.012	1	1
rs269110	1	112284484	0.022	0.043	0.005		
rs4378202	1	113043176	0.023	0.049	0.013		
rs10494155	1	113602512	0.030	0.053	0.022		
rs1935745	1	113606669	0.024	0.018	0.036		
rs10494164	1	114258570	0.048	0.060	0.003	1	
rs4274061	1	114267379	0.023	0.009	0.040		
rs6681880	1	115331464	0.034	0.065	0.009	1	
rs195176	1	115467753	0.074	0.032	0.000	1	1
rs3884586	1	115848867	0.027	0.000	0.032		
rs1655200	1	118051391	0.023	0.043	0.000		
rs1830853	1	118781152	0.043	0.061	0.019	1	
rs10754391	1	119341746	0.022	0.046	0.004		
rs699774	1	119607263	0.017	0.018	0.037		
rs3009197	1	120072040	0.027	0.043	0.003		
rs4926386	1	146699208	0.018	0.000	0.032		
rs3790506	1	148351439	0.037	0.083	0.007	1	1
rs12066445	1	148553493	0.043	0.000	0.080	1	1
rs6587641	1	148791112	0.059	0.075	0.005	1	
rs6662637	1	149323421	0.037	0.011	0.035		
rs10888541	1	150022661	0.029	0.002	0.032		
rs4661162	1	152737578	0.023	0.051	0.005		
rs2050568	1	154583314	0.035	0.063	0.013	1	
rs2188114	1	155646823	0.014	0.007	0.032		
rs11265265	1	156526629	0.064	0.001	0.008	1	1
rs905594	1	158472932	0.057	0.059	0.000	1	
rs954280	1	159684237	0.025	0.025	0.057	1	1
rs1891733	1	161539488	0.013	0.017	0.032		
rs10489744	1	162112281	0.023	0.015	0.050	1	1
rs7522756	1	162693596	0.032	0.056	0.001		

rs909935	1	164264672	0.045	0.047	0.000		
rs1810467	1	165144879	0.034	0.025	0.042	1	
rs7551639	1	165339810	0.028	0.051	0.017		
rs2420511	1	166544241	0.038	0.063	0.060	1	1
rs513287	1	167395895	0.019	0.019	0.032		
rs10910863	1	168692650	0.017	0.001	0.033		
rs479336	1	168931230	0.021	0.043	0.003		
rs1546844	1	171989307	0.035	0.060	0.032		
rs10798580	1	174596211	0.055	0.032	0.003		
rs10157480	1	174604990	0.041	0.064	0.000	1	
rs1410594	1	176314822	0.060	0.000	0.062	1	
rs2504057	1	176418916	0.034	0.052	0.000		
rs2764453	1	176980964	0.058	0.057	0.001	1	
rs12565492	1	177222352	0.052	0.105	0.036		
rs2609473	1	178067164	0.021	0.047	0.008		
rs2225875	1	178238196	0.022	0.015	0.032		
rs1409810	1	180922096	0.020	0.008	0.032		
rs4651287	1	182448621	0.071	0.081	0.000	1	1
rs723848	1	184628041	0.062	0.018	0.043	1	
rs4571932	1	185045487	0.038	0.090	0.005	1	1
rs815721	1	186675709	0.020	0.018	0.032		
rs1572474	1	188318629	0.039	0.064	0.040		
rs2785761	1	188451084	0.054	0.008	0.036		
rs4657856	1	190056062	0.019	0.043	0.023		
rs12085890	1	194726752	0.042	0.012	0.036		
rs6667273	1	195877214	0.030	0.045	0.002		
rs4915403	1	196698219	0.020	0.014	0.039		
rs3795600	1	199441977	0.085	0.124	0.002	1	1
rs4951373	1	201123914	0.030	0.058	0.009	1	
rs12025272	1	201601216	0.022	0.017	0.045	1	
rs823088	1	202525832	0.018	0.009	0.035		
rs2232360	1	203429054	0.036	0.052	0.000		
rs2184820	1	204530627	0.026	0.056	0.012		
rs2495295	1	205878083	0.020	0.049	0.006		
rs1002382	1	206701155	0.014	0.015	0.032		
rs1538757	1	207281628	0.069	0.007	0.020	1	1
rs1361193	1	207771524	0.067	0.116	0.003	1	1
rs11119953	1	209059389	0.019	0.000	0.040		
rs3795842	1	209188344	0.026	0.064	0.010	1	
rs1341152	1	210387829	0.055	0.096	0.003		
rs7528988	1	211636812	0.022	0.043	0.005		
rs11120677	1	212422445	0.029	0.065	0.038	1	
rs1006236	1	212426040	0.031	0.039	0.047	1	
rs2820879	1	213272038	0.021	0.031	0.037		
rs2789557	1	213547605	0.027	0.056	0.006		
rs2788193	1	214846622	0.069	0.067	0.000	1	1
rs12038116	1	215107795	0.021	0.000	0.032		
rs10779363	1	216175285	0.046	0.053	0.003		
rs1338243	1	216327071	0.025	0.000	0.038		
rs6541130	1	216879546	0.026	0.011	0.047	1	
rs6604694	1	218560800	0.015	0.003	0.032		

rs2000319	1	220192578	0.023	0.022	0.050	1	1
rs1010735	1	221222164	0.032	0.063	0.000	1	
rs10495234	1	221800318	0.031	0.047	0.037		
rs11577454	1	222795561	0.032	0.065	0.002	1	
rs1319103	1	223526049	0.028	0.003	0.040		
rs748405	1	224254169	0.020	0.017	0.032		
rs1925716	1	225014274	0.052	0.101	0.000	1	1
rs12097506	1	225156336	0.058	0.056	0.002	1	
rs3811473	1	226079428	0.061	0.001	0.008	1	
rs4925505	1	226116995	0.029	0.034	0.037		
rs1890350	1	227201100	0.019	0.015	0.036		
rs545937	1	227850278	0.036	0.052	0.004		
rs821717	1	228366540	0.049	0.071	0.032		
rs12567201	1	229105689	0.037	0.052	0.003		
rs1294318	1	229842911	0.026	0.015	0.066	1	1
rs6667354	1	230090957	0.032	0.061	0.004	1	
rs6659304	1	231188184	0.058	0.077	0.001	1	
rs582666	1	232925149	0.025	0.008	0.040		
rs4659455	1	232949864	0.028	0.045	0.000		
rs7538075	1	233781064	0.016	0.011	0.035		
rs2805446	1	234019231	0.058	0.065	0.006	1	
rs10495406	1	234706771	0.024	0.046	0.002		
rs1553385	1	235144403	0.037	0.011	0.033		
rs9428757	1	235377966	0.021	0.045	0.002		
rs536477	1	236371949	0.058	0.077	0.003	1	1
rs12093992	1	236486163	0.060	0.018	0.010	1	
rs9662100	1	236905300	0.038	0.079	0.000	1	1
rs1341463	1	237851563	0.041	0.074	0.015	1	1
rs2050513	1	238030789	0.023	0.030	0.049	1	
rs6695943	1	241203996	0.058	0.000	0.027	1	
rs10924373	1	242365272	0.032	0.001	0.064	1	1
rs10924763	1	243007157	0.015	0.002	0.033		
rs3737277	1	244350007	0.044	0.079	0.016	1	1
rs4589796	2	816069	0.056	0.000	0.014	1	
rs10165034	2	3945253	0.056	0.071	0.000	1	
rs17360123	2	5564812	0.062	0.023	0.029	1	
rs6432010	2	5568279	0.041	0.007	0.032		
rs12692317	2	6607073	0.039	0.073	0.013	1	1
rs307973	2	6791928	0.063	0.010	0.006	1	1
rs1566982	2	6874765	0.024	0.000	0.032		
rs454999	2	7113477	0.038	0.051	0.000		
rs7582203	2	7763697	0.031	0.050	0.022		
rs1423712	2	8079464	0.054	0.012	0.002		
rs6431940	2	8552876	0.039	0.047	0.004		
rs4669347	2	9108760	0.061	0.084	0.001	1	
rs4668600	2	9185014	0.058	0.086	0.000	1	1
rs4668654	2	10015175	0.030	0.051	0.013		
rs4143555	2	11068415	0.028	0.058	0.036		
rs10495577	2	11074113	0.028	0.059	0.025	1	
rs10520097	2	11883984	0.037	0.051	0.005		
rs1401697	2	12732051	0.057	0.005	0.026	1	

rs4669869	2	12849058	0.024	0.052	0.008		
rs1818595	2	13118830	0.026	0.001	0.041		
rs6432444	2	14208966	0.032	0.062	0.029	1	
rs1434975	2	14756040	0.034	0.004	0.034		
rs2333979	2	16381164	0.026	0.051	0.053	1	1
rs2193698	2	16969779	0.020	0.002	0.040		
rs1377082	2	18255498	0.030	0.062	0.011	1	
rs6718502	2	19156206	0.031	0.004	0.058	1	1
rs1579204	2	19644542	0.035	0.056	0.006		
rs3731661	2	20032484	0.054	0.013	0.031		
rs931257	2	20610569	0.045	0.070	0.004	1	1
rs9085	2	20806601	0.034	0.006	0.037		
rs6547953	2	21957369	0.069	0.000	0.034	1	1
rs328539	2	22213665	0.043	0.000	0.059	1	1
rs10195540	2	22914905	0.055	0.006	0.010		
rs7603997	2	24436609	0.043	0.070	0.052	1	1
rs6751254	2	25617079	0.022	0.052	0.008		
rs4450561	2	26565108	0.025	0.052	0.009		
rs1522098	2	26669225	0.023	0.003	0.041		
rs4665960	2	27379249	0.066	0.030	0.002	1	1
rs2338545	2	28711426	0.042	0.054	0.001		
rs2028983	2	30211687	0.023	0.017	0.044	1	
rs829597	2	30550125	0.039	0.069	0.037	1	1
rs1862969	2	31148567	0.059	0.012	0.001	1	
rs8179695	2	31194996	0.020	0.048	0.010		
rs1346007	2	34217210	0.041	0.082	0.014	1	1
rs4327215	2	34440668	0.038	0.002	0.039		
rs10495861	2	36166474	0.028	0.069	0.007	1	
rs3821147	2	36922225	0.034	0.003	0.034		
rs4670624	2	36957770	0.041	0.052	0.000		
rs163077	2	38197256	0.017	0.007	0.041		
rs162330	2	38231147	0.028	0.057	0.000		
rs2043188	2	38840632	0.030	0.000	0.032		
rs2716694	2	39822987	0.024	0.036	0.042		
rs1866670	2	42221973	0.016	0.003	0.037		
rs895514	2	43122857	0.076	0.036	0.000	1	1
rs11683623	2	44228564	0.034	0.007	0.040		
rs7583873	2	45399117	0.024	0.051	0.001		
rs883413	2	45441246	0.061	0.010	0.060	1	
rs3820729	2	46304426	0.027	0.064	0.011	1	
rs4953413	2	46830688	0.066	0.072	0.009	1	1
rs2241736	2	47099044	0.032	0.001	0.034		
rs7559733	2	47769306	0.021	0.043	0.002		
rs6732149	2	48310017	0.035	0.051	0.003		
rs1015823	2	50599529	0.097	0.055	0.008	1	1
rs2194390	2	50814582	0.048	0.071	0.000	1	1
rs6706076	2	51618083	0.026	0.054	0.002		
rs10205157	2	52178025	0.034	0.072	0.001	1	1
rs2552356	2	52566395	0.031	0.000	0.041		
rs2356473	2	52847053	0.023	0.053	0.009		
rs11125515	2	53798450	0.070	0.000	0.029	1	1

rs805360	2	54076499	0.051	0.028	0.051	1	1
rs11894205	2	54354348	0.026	0.045	0.016		
rs698752	2	54365461	0.073	0.035	0.010	1	1
rs11677058	2	55009200	0.025	0.006	0.054	1	1
rs3850354	2	56237947	0.044	0.059	0.003	1	
rs7557284	2	57253099	0.027	0.048	0.059	1	1
rs13405071	2	58004405	0.014	0.025	0.032		
rs17644588	2	59491977	0.032	0.070	0.018	1	1
rs1005038	2	60017333	0.042	0.005	0.076	1	1
rs6735059	2	60090964	0.072	0.029	0.004	1	1
rs1861151	2	60196265	0.028	0.044	0.022		
rs4563212	2	62414668	0.033	0.045	0.000		
rs7603526	2	62505283	0.067	0.037	0.017	1	1
rs2710638	2	63035265	0.030	0.045	0.007		
rs10496114	2	64579904	0.056	0.091	0.004	1	1
rs6729083	2	64592618	0.058	0.060	0.000	1	
rs10179158	2	65923683	0.043	0.071	0.001	1	1
rs12713568	2	66653889	0.041	0.001	0.079	1	1
rs13385154	2	67424769	0.078	0.084	0.012	1	1
rs10490726	2	67778193	0.031	0.001	0.042	1	
rs6759864	2	68482804	0.042	0.056	0.000		
rs6760496	2	71146326	0.041	0.062	0.009	1	
rs227782	2	71778497	0.027	0.017	0.066	1	1
rs2900976	2	71870135	0.028	0.053	0.028		
rs6714189	2	75592456	0.046	0.072	0.019	1	1
rs13019578	2	75614326	0.039	0.017	0.062	1	1
rs436000	2	76152016	0.056	0.013	0.002	1	
rs10519330	2	76597623	0.054	0.074	0.000	1	1
rs10520170	2	77081856	0.045	0.000	0.057	1	1
rs6715934	2	78379067	0.021	0.014	0.048	1	
rs4852150	2	79649777	0.074	0.118	0.000	1	1
rs2974142	2	80073853	0.027	0.009	0.038		
rs2043015	2	83533819	0.023	0.042	0.006		
rs4832067	2	84241961	0.027	0.004	0.055	1	1
rs4832163	2	85452741	0.034	0.008	0.037		
rs2288123	2	86297386	0.023	0.026	0.047	1	
rs1033287	2	86561405	0.055	0.099	0.026		
rs2176569	2	88315550	0.033	0.080	0.023	1	1
rs7574579	2	98272572	0.022	0.050	0.003		
rs2009094	2	100223595	0.019	0.012	0.045	1	
rs6748892	2	100324563	0.024	0.057	0.005		
rs3768984	2	101009505	0.030	0.071	0.005	1	1
rs1110947	2	101577230	0.026	0.016	0.047	1	
rs4851475	2	101720114	0.026	0.045	0.028		
rs4399750	2	102376306	0.026	0.024	0.047	1	
rs2732821	2	102888214	0.020	0.042	0.017		
rs6761331	2	105249157	0.042	0.053	0.003		
rs2118291	2	105444189	0.037	0.000	0.047	1	
rs333234	2	108065912	0.064	0.002	0.014	1	1
rs1470874	2	108337229	0.040	0.025	0.078	1	1
rs3747653	2	111682771	0.042	0.007	0.035		

rs4308124	2	111726717	0.061	0.059	0.009	1	
rs6746923	2	113269657	0.042	0.096	0.036		
rs17197611	2	114360283	0.033	0.005	0.070	1	1
rs3105488	2	114628299	0.059	0.096	0.002	1	
rs998429	2	115363813	0.023	0.049	0.001		
rs870925	2	115863267	0.018	0.002	0.036		
rs1374597	2	116449567	0.017	0.004	0.038		
rs4848429	2	117443275	0.020	0.031	0.037		
rs10192071	2	118922071	0.024	0.045	0.000		
rs2304708	2	119465707	0.033	0.000	0.052	1	1
rs10496555	2	119595818	0.022	0.045	0.006		
rs7562511	2	120894482	0.031	0.066	0.002	1	
rs2580347	2	121800658	0.063	0.010	0.005	1	1
rs1453675	2	122856109	0.018	0.003	0.034		
rs10169108	2	122926574	0.038	0.048	0.002		
rs1881137	2	123387431	0.027	0.041	0.040		
rs2419976	2	123996379	0.020	0.002	0.043	1	
rs13008660	2	124219435	0.035	0.052	0.009		
rs779984	2	124844026	0.022	0.000	0.033		
rs2028211	2	127617887	0.026	0.012	0.056	1	1
rs4663114	2	127654208	0.023	0.050	0.032		
rs4662834	2	129195072	0.024	0.034	0.033		
rs10210526	2	129804947	0.023	0.042	0.011		
rs6732952	2	129900012	0.021	0.030	0.044	1	
rs2672124	2	131371917	0.015	0.023	0.034		
rs1835429	2	133333569	0.018	0.007	0.044	1	
rs1519305	2	134647518	0.031	0.065	0.023	1	
rs892878	2	137705592	0.037	0.070	0.002	1	1
rs986447	2	137822103	0.016	0.009	0.036		
rs6738271	2	138479416	0.018	0.042	0.010		
rs938827	2	140366967	0.026	0.062	0.007	1	
rs2129483	2	140838261	0.020	0.008	0.042	1	
rs10496858	2	141473377	0.058	0.013	0.001	1	
rs13016717	2	141551378	0.019	0.009	0.037		
rs1020426	2	143106635	0.069	0.000	0.000	1	1
rs1017995	2	143419269	0.042	0.059	0.001	1	
rs17689833	2	145132055	0.022	0.013	0.035		
rs4662248	2	146189188	0.057	0.086	0.018	1	
rs4632277	2	146238081	0.057	0.123	0.009	1	1
rs2552546	2	146888684	0.020	0.009	0.038		
rs6760166	2	147788051	0.067	0.018	0.062	1	1
rs1522663	2	147953795	0.052	0.113	0.004	1	1
rs4265953	2	149084454	0.021	0.000	0.035		
rs6735775	2	150679504	0.045	0.045	0.006		
rs6727863	2	150841582	0.055	0.007	0.007	1	
rs2190368	2	151578283	0.035	0.003	0.035		
rs10930342	2	151619135	0.057	0.055	0.015	1	
rs12618502	2	151635289	0.054	0.065	0.005	1	
rs2444263	2	152137078	0.064	0.000	0.002	1	1
rs7568759	2	152980120	0.027	0.043	0.008		
rs808948	2	155022869	0.038	0.000	0.042	1	

rs1993788	2	157436402	0.031	0.052	0.001		
rs2289942	2	157966225	0.026	0.053	0.022		
rs155594	2	158095650	0.032	0.001	0.034		
rs1011770	2	160064225	0.055	0.108	0.062		
rs1425044	2	160300444	0.039	0.036	0.081	1	1
rs10221808	2	162639314	0.032	0.045	0.001		
rs16850017	2	165688110	0.033	0.072	0.017	1	1
rs12996382	2	166885402	0.038	0.000	0.042	1	
rs1511200	2	167623610	0.029	0.063	0.005	1	
rs734284	2	168286668	0.035	0.068	0.024	1	
rs7577337	2	169044983	0.054	0.116	0.000	1	1
rs977171	2	169109525	0.064	0.074	0.009	1	1
rs830994	2	169955035	0.036	0.013	0.048	1	
rs700550	2	169983963	0.036	0.066	0.018	1	
rs4668261	2	171169796	0.029	0.059	0.001	1	
rs1362487	2	171466449	0.071	0.003	0.035	1	1
rs2884005	2	172093568	0.021	0.000	0.033		
rs1843758	2	172823277	0.028	0.057	0.001		
rs3754751	2	173712526	0.030	0.045	0.003		
rs6731322	2	173942527	0.020	0.001	0.037		
rs3769148	2	173954020	0.074	0.039	0.030	1	1
rs2043920	2	174364616	0.039	0.044	0.000		
rs9287970	2	174742814	0.022	0.037	0.036		
rs6433615	2	177479264	0.030	0.008	0.032		
rs4893980	2	178380539	0.047	0.053	0.005		
rs334032	2	178911586	0.070	0.031	0.010	1	1
rs6433796	2	180248569	0.022	0.052	0.027		
rs7566676	2	180818007	0.053	0.080	0.001	1	1
rs11900518	2	180834466	0.055	0.053	0.002		
rs4077924	2	181816895	0.033	0.051	0.005		
rs13409979	2	184179316	0.032	0.025	0.047	1	
rs9678612	2	184350715	0.037	0.046	0.002		
rs2054548	2	185327530	0.034	0.000	0.037		
rs850880	2	186030695	0.038	0.060	0.004	1	
rs1424528	2	186065113	0.018	0.025	0.033		
rs10188692	2	190297682	0.018	0.003	0.037		
rs3024908	2	191719647	0.044	0.068	0.002	1	
rs11890239	2	192468528	0.025	0.043	0.039		
rs4853669	2	193012287	0.054	0.010	0.009		
rs1484900	2	193193507	0.029	0.056	0.011		
rs2360101	2	195187820	0.019	0.010	0.040		
rs6727480	2	197969377	0.042	0.083	0.041	1	1
rs2196176	2	198730420	0.021	0.045	0.017		
rs4850839	2	198973924	0.035	0.008	0.067	1	1
rs966744	2	199635222	0.028	0.050	0.011		
rs6752494	2	200138829	0.029	0.013	0.054	1	1
rs2348121	2	201436090	0.045	0.000	0.070	1	1
rs6435268	2	205715152	0.032	0.050	0.001		
rs12694049	2	206969310	0.019	0.021	0.032		
rs2059304	2	207193027	0.027	0.055	0.003		
rs2359858	2	207631937	0.043	0.006	0.044	1	

rs6710342	2	208032823	0.071	0.138	0.025	1	1
rs3769521	2	208942259	0.028	0.045	0.000		
rs6714124	2	211261571	0.024	0.047	0.001		
rs10932380	2	212215856	0.029	0.044	0.000		
rs6712126	2	212741663	0.026	0.000	0.040		
rs2372258	2	214543244	0.066	0.097	0.000	1	1
rs6749380	2	215065449	0.056	0.000	0.013	1	
rs4362557	2	215190556	0.041	0.015	0.066	1	1
rs4672775	2	216284480	0.032	0.070	0.004	1	1
rs10498052	2	216729905	0.059	0.005	0.018	1	
rs6728171	2	217267457	0.032	0.062	0.002	1	
rs10204522	2	217472344	0.018	0.006	0.034		
rs2373072	2	218199572	0.031	0.041	0.002		
rs2042542	2	218561798	0.031	0.025	0.036		
rs10498070	2	220523630	0.026	0.001	0.050	1	
rs6741459	2	221055345	0.052	0.053	0.017		
rs997701	2	221056844	0.056	0.029	0.001	1	
rs2392816	2	221706486	0.025	0.057	0.010		
rs1583236	2	221884272	0.022	0.004	0.035		
rs824939	2	222647066	0.041	0.078	0.000	1	1
rs6738997	2	222686780	0.058	0.010	0.002	1	
rs10932956	2	223152229	0.031	0.005	0.036		
rs1523921	2	225134483	0.067	0.037	0.003	1	1
rs4347829	2	226044825	0.021	0.001	0.034		
rs891583	2	226225975	0.040	0.045	0.000		
rs1273829	2	227291702	0.033	0.061	0.000	1	
rs6727477	2	227885176	0.066	0.000	0.108	1	1
rs4321359	2	228349815	0.040	0.087	0.011	1	1
rs12471368	2	229506592	0.025	0.006	0.037		
rs1544688	2	229699784	0.026	0.048	0.000		
rs7604147	2	231423368	0.055	0.031	0.032		
rs10933358	2	231516486	0.041	0.005	0.052	1	1
rs4973588	2	233660480	0.028	0.062	0.010	1	
rs2241880	2	233965368	0.034	0.000	0.049	1	
rs12996139	2	234555441	0.044	0.032	0.053	1	1
rs12994993	2	235532718	0.028	0.052	0.002		
rs3731646	2	235732002	0.029	0.003	0.045	1	
rs6754331	2	236223590	0.056	0.032	0.000	1	
rs7557044	2	236714554	0.032	0.000	0.043	1	
rs13000320	2	237505694	0.026	0.059	0.023	1	
rs10929201	2	237614366	0.022	0.020	0.049	1	
rs6431565	2	238618683	0.027	0.007	0.032		
rs4852033	2	239606151	0.025	0.045	0.014		
rs3755534	2	241401754	0.054	0.062	0.004		
rs10049372	3	682654	0.054	0.033	0.000		
rs6442164	3	1071770	0.042	0.047	0.001		
rs718559	3	1469475	0.033	0.028	0.034		
rs12495067	3	2016956	0.014	0.025	0.032		
rs9310717	3	2375576	0.060	0.030	0.001	1	
rs2675307	3	2700060	0.037	0.062	0.016	1	
rs7629924	3	3042850	0.023	0.002	0.051	1	1

rs10510268	3	3539506	0.021	0.043	0.027		
rs11130036	3	4518463	0.041	0.040	0.059	1	1
rs4685827	3	4839877	0.065	0.009	0.008	1	1
rs4684476	3	5340063	0.045	0.058	0.002	1	
rs161885	3	7772492	0.023	0.003	0.038		
rs162212	3	7814477	0.034	0.064	0.000	1	
rs7626393	3	8470425	0.062	0.062	0.004	1	
rs4470492	3	10561843	0.038	0.088	0.012	1	1
rs2028760	3	12347882	0.026	0.047	0.007		
rs2648701	3	13041535	0.026	0.044	0.001		
rs1350344	3	14192580	0.029	0.000	0.036		
rs900174	3	14255140	0.023	0.049	0.011		
rs744892	3	14846770	0.031	0.052	0.018		
rs480644	3	15099806	0.022	0.028	0.033		
rs13075694	3	15425404	0.034	0.067	0.013	1	
rs689960	3	16393216	0.027	0.000	0.039		
rs7624893	3	16408305	0.062	0.022	0.037	1	
rs4077616	3	17148419	0.018	0.001	0.042	1	
rs6577628	3	18302350	0.083	0.016	0.000	1	1
rs1513755	3	18505983	0.048	0.063	0.002	1	
rs6785093	3	18578117	0.020	0.027	0.042	1	
rs6804525	3	19628391	0.027	0.057	0.018		
rs1466962	3	20590936	0.025	0.050	0.018		
rs7619783	3	21781436	0.038	0.045	0.040		
rs1496653	3	23429794	0.027	0.006	0.037		
rs826219	3	24244605	0.059	0.049	0.000	1	
rs12496073	3	25008192	0.030	0.056	0.000		
rs11129210	3	25860034	0.019	0.025	0.042	1	
rs7626646	3	27243320	0.029	0.010	0.044	1	
rs4680899	3	28094644	0.040	0.057	0.001		
rs2218746	3	28991443	0.025	0.053	0.007		
rs7431530	3	29357545	0.025	0.011	0.054	1	1
rs1594827	3	30226040	0.072	0.013	0.028	1	1
rs2132298	3	30377867	0.043	0.002	0.075	1	1
rs795432	3	30716794	0.043	0.066	0.001	1	
rs11058	3	31678468	0.040	0.092	0.004	1	1
rs4955278	3	32340453	0.018	0.002	0.036		
rs4076661	3	34036426	0.030	0.043	0.004		
rs7642509	3	34265040	0.024	0.006	0.058	1	1
rs1529079	3	34519264	0.054	0.004	0.019		
rs6550422	3	36395632	0.062	0.086	0.003	1	
rs1053006	3	37199103	0.038	0.000	0.047	1	
rs9866555	3	37711803	0.033	0.035	0.062	1	1
rs3922844	3	38599257	0.019	0.015	0.044	1	
rs11716493	3	38789887	0.057	0.042	0.002	1	
rs6769485	3	40205410	0.025	0.024	0.051	1	1
rs7639223	3	40260164	0.019	0.043	0.004		
rs9838277	3	41278091	0.020	0.002	0.033		
rs900569	3	41834977	0.032	0.001	0.044	1	
rs7633017	3	42187738	0.038	0.052	0.004		
rs2372580	3	44024949	0.054	0.003	0.012		

rs9819447	3	45032630	0.039	0.033	0.074	1	1
rs1463680	3	45971765	0.023	0.008	0.047	1	
rs1979672	3	46249113	0.020	0.049	0.021		
rs1042779	3	52796051	0.055	0.045	0.001		
rs3736151	3	53234648	0.036	0.064	0.001	1	
rs3774503	3	53652225	0.017	0.003	0.033		
rs6766099	3	53869452	0.025	0.056	0.003		
rs934083	3	54765419	0.022	0.048	0.021		
rs4955912	3	54971488	0.035	0.003	0.049	1	
rs2120785	3	55285731	0.029	0.070	0.010	1	1
rs4681888	3	56805127	0.036	0.051	0.005		
rs4681869	3	58548574	0.033	0.010	0.033		
rs4289361	3	58562496	0.036	0.055	0.002		
rs1443941	3	59303257	0.029	0.057	0.000		
rs717821	3	60490818	0.031	0.054	0.000		
rs1904347	3	61929413	0.021	0.004	0.045	1	
rs833668	3	62590338	0.029	0.009	0.040		
rs1398096	3	62636049	0.030	0.062	0.018	1	
rs7615771	3	64616738	0.032	0.017	0.052	1	1
rs1482596	3	65043188	0.049	0.054	0.005		
rs4501148	3	65696308	0.026	0.009	0.032		
rs984038	3	65737292	0.033	0.048	0.001		
rs9814525	3	67177665	0.038	0.080	0.005	1	1
rs745052	3	67669015	0.034	0.071	0.044	1	
rs1459912	3	67869427	0.055	0.128	0.018		
rs12635949	3	69029799	0.033	0.050	0.011		
rs7647186	3	69623185	0.022	0.045	0.020		
rs4677611	3	71490580	0.036	0.024	0.072	1	1
rs9856074	3	72095067	0.026	0.053	0.003		
rs2322272	3	72259305	0.026	0.001	0.051	1	1
rs10511022	3	72626886	0.037	0.077	0.006	1	1
rs1532190	3	73086847	0.031	0.012	0.047	1	
rs291485	3	73961357	0.024	0.005	0.039		
rs6772123	3	74098459	0.032	0.056	0.001		
rs1488798	3	74504266	0.064	0.022	0.001	1	1
rs7431887	3	74612282	0.038	0.070	0.005	1	1
rs9840407	3	74753871	0.014	0.004	0.034		
rs998037	3	77429653	0.028	0.045	0.007		
rs2055707	3	78706269	0.019	0.000	0.032		
rs4264688	3	79629038	0.039	0.000	0.032		
rs4396907	3	83989494	0.019	0.043	0.009		
rs1037231	3	85845797	0.027	0.054	0.002		
rs4571247	3	89149278	0.021	0.050	0.011		
rs1512912	3	89390621	0.030	0.000	0.049	1	
rs9290469	3	95067163	0.015	0.011	0.034		
rs13081582	3	95977726	0.032	0.061	0.007	1	
rs2221205	3	98357050	0.028	0.043	0.004		
rs278376	3	99975821	0.033	0.064	0.013	1	
rs6793682	3	101840361	0.020	0.001	0.037		
rs2263357	3	105681807	0.028	0.001	0.034		
rs2633682	3	105891898	0.025	0.045	0.004		

rs1159290	3	107241839	0.057	0.081	0.000	1	
rs968072	3	107669610	0.021	0.004	0.040		
rs4358307	3	110071085	0.027	0.048	0.011		
rs1177385	3	110409180	0.022	0.001	0.049	1	
rs9832962	3	110739100	0.065	0.064	0.003	1	1
rs6437989	3	112686381	0.025	0.007	0.039		
rs9864383	3	113264489	0.023	0.002	0.036		
rs2399460	3	114254195	0.018	0.018	0.044	1	
rs6782826	3	114302501	0.025	0.051	0.033		
rs2718419	3	115619659	0.039	0.007	0.053	1	1
rs1818768	3	118321694	0.052	0.074	0.008	1	1
rs12494616	3	118444511	0.024	0.027	0.046	1	
rs10511370	3	118808667	0.055	0.000	0.000		
rs982040	3	119925186	0.018	0.014	0.034		
rs9713311	3	120558458	0.030	0.030	0.046	1	
rs334547	3	121322559	0.031	0.000	0.037		
rs322457	3	122072417	0.025	0.056	0.003		
rs1464099	3	122807333	0.021	0.008	0.032		
rs1523519	3	123114531	0.058	0.097	0.001	1	
rs1909808	3	123808157	0.030	0.051	0.000		
rs1343700	3	125054444	0.075	0.084	0.000	1	1
rs3755702	3	125897144	0.071	0.078	0.006	1	1
rs3772836	3	126018325	0.027	0.000	0.041		
rs6777173	3	127571036	0.024	0.001	0.040		
rs729154	3	128359718	0.046	0.086	0.000	1	1
rs729153	3	128359783	0.055	0.063	0.001		
rs6439099	3	129114259	0.039	0.004	0.037		
rs10934861	3	129910437	0.055	0.002	0.011		
rs6771574	3	130186400	0.021	0.015	0.038		
rs4073085	3	130986725	0.025	0.048	0.016		
rs2718872	3	132354747	0.054	0.069	0.000		
rs11710141	3	132632964	0.027	0.014	0.042	1	
rs11714960	3	132909883	0.048	0.091	0.005	1	1
rs1225071	3	133161260	0.066	0.040	0.002	1	1
rs13059279	3	134723804	0.023	0.000	0.039		
rs2715609	3	135032636	0.017	0.042	0.008		
rs9843732	3	135505754	0.022	0.002	0.033		
rs11920780	3	136125365	0.045	0.057	0.007		
rs39699	3	136180882	0.059	0.001	0.015	1	
rs3912122	3	138538014	0.016	0.006	0.037		
rs9289559	3	139573887	0.034	0.029	0.054	1	1
rs661711	3	140237657	0.058	0.051	0.005	1	
rs1026775	3	141567136	0.042	0.001	0.061	1	1
rs1426955	3	142235236	0.029	0.005	0.037		
rs9850416	3	142754914	0.021	0.001	0.045	1	
rs13064646	3	145999262	0.020	0.005	0.035		
rs4073319	3	146354509	0.060	0.027	0.001	1	
rs10513274	3	147168224	0.059	0.088	0.000	1	
rs9867807	3	147250382	0.028	0.000	0.039		
rs342892	3	147817329	0.029	0.061	0.014	1	
rs9873630	3	148246089	0.075	0.007	0.041	1	1

rs1354053	3	148468462	0.042	0.077	0.018	1	1
rs10935749	3	150582273	0.028	0.022	0.039		
rs7616812	3	150848198	0.034	0.065	0.011	1	
rs7627651	3	151404783	0.018	0.007	0.038		
rs16862676	3	151661637	0.065	0.041	0.004	1	1
rs1703802	3	152230849	0.026	0.000	0.041		
rs6778082	3	152607619	0.033	0.074	0.006	1	1
rs1715649	3	153263633	0.071	0.066	0.004	1	1
rs323614	3	153382422	0.062	0.069	0.002	1	
rs11709568	3	156660035	0.032	0.002	0.054	1	1
rs2201932	3	156976617	0.044	0.050	0.000		
rs10804774	3	157136719	0.063	0.017	0.002	1	1
rs11924934	3	157684070	0.036	0.005	0.040		
rs4254605	3	157721237	0.057	0.018	0.037	1	
rs1367311	3	157901458	0.040	0.057	0.000		
rs2200883	3	160731525	0.021	0.050	0.013		
rs1378661	3	161907001	0.049	0.005	0.038		
rs1387010	3	163420667	0.054	0.035	0.011		
rs12633503	3	163565385	0.026	0.037	0.054	1	1
rs12488754	3	163568522	0.025	0.047	0.040		
rs9878865	3	165846074	0.028	0.011	0.048	1	
rs6548380	3	166141326	0.031	0.047	0.004		
rs10513619	3	166897539	0.057	0.025	0.019	1	
rs7627395	3	167149256	0.037	0.047	0.000		
rs998749	3	170455504	0.044	0.096	0.007	1	1
rs16853620	3	170582819	0.038	0.061	0.041		
rs499061	3	171890860	0.013	0.003	0.032		
rs9872465	3	172997533	0.031	0.000	0.048	1	
rs13092828	3	173006892	0.068	0.003	0.040	1	1
rs7642511	3	173528815	0.018	0.006	0.040		
rs1499626	3	174065304	0.038	0.001	0.037		
rs7649709	3	174612521	0.073	0.143	0.003	1	1
rs563892	3	175919503	0.035	0.055	0.000		
rs6443305	3	176700708	0.058	0.020	0.013	1	
rs9882167	3	177250078	0.027	0.047	0.000		
rs2220099	3	177528438	0.032	0.016	0.060	1	1
rs7429900	3	180052167	0.027	0.049	0.032		
rs6443590	3	180052629	0.025	0.060	0.020	1	
rs1805589	3	182130112	0.079	0.052	0.003	1	1
rs1871355	3	182628753	0.043	0.071	0.005	1	1
rs482912	3	184336372	0.018	0.023	0.038		
rs17543340	3	184687625	0.026	0.062	0.023	1	
rs902417	3	185434133	0.025	0.040	0.034		
rs2230571	3	185529172	0.057	0.024	0.010	1	
rs10937169	3	185613733	0.024	0.055	0.011		
rs4686709	3	187112001	0.038	0.057	0.005		
rs16862964	3	189275413	0.025	0.002	0.050	1	1
rs12494557	3	189533236	0.030	0.045	0.001		
rs1055152	3	190521350	0.031	0.050	0.000		
rs2362605	3	191962879	0.025	0.055	0.020		
rs7618303	3	192618260	0.029	0.050	0.002		

rs9851984	3	194573508	0.059	0.105	0.024	1	
rs951016	3	194576404	0.050	0.099	0.072	1	1
rs2630237	3	195073947	0.035	0.081	0.062	1	1
rs2293232	3	196986690	0.055	0.134	0.018		
rs768858	3	198564031	0.020	0.048	0.019		
rs1203860	4	2472322	0.023	0.008	0.049	1	
rs2748777	4	3756798	0.027	0.018	0.059	1	1
rs10027109	4	4464309	0.015	0.018	0.032		
rs3733397	4	4967474	0.022	0.045	0.000		
rs4513510	4	5839846	0.021	0.005	0.045	1	
rs1871584	4	5863792	0.027	0.057	0.007		
rs4327561	4	6499395	0.027	0.056	0.041		
rs9291117	4	6565679	0.054	0.017	0.003		
rs4696709	4	8084883	0.018	0.005	0.040		
rs1519097	4	9479122	0.061	0.009	0.001	1	
rs4697695	4	9592119	0.026	0.060	0.013	1	
rs6448182	4	10369491	0.029	0.027	0.040		
rs1533338	4	10943855	0.020	0.007	0.042	1	
rs1454884	4	11932770	0.034	0.059	0.000	1	
rs1476211	4	13218305	0.021	0.034	0.032		
rs10939499	4	13374230	0.023	0.052	0.014		
rs3111811	4	13939470	0.031	0.005	0.068	1	1
rs11944971	4	14549749	0.028	0.008	0.042	1	
rs16893097	4	15833233	0.032	0.047	0.020		
rs7660122	4	15980583	0.067	0.000	0.049	1	1
rs16893797	4	16371132	0.030	0.051	0.002		
rs11731260	4	16794033	0.023	0.008	0.032		
rs13434430	4	19986442	0.023	0.000	0.040		
rs10155343	4	20635557	0.014	0.006	0.035		
rs919856	4	20759822	0.037	0.070	0.014	1	1
rs412218	4	22519727	0.029	0.053	0.014		
rs215410	4	23100329	0.035	0.000	0.033		
rs1511454	4	23258750	0.029	0.043	0.004		
rs2324153	4	23703606	0.059	0.004	0.008	1	
rs7656884	4	23834298	0.022	0.043	0.004		
rs6823107	4	24121054	0.045	0.005	0.035		
rs7682064	4	24763977	0.023	0.013	0.035		
rs316783	4	25018732	0.024	0.054	0.004		
rs10939088	4	25663865	0.023	0.030	0.039		
rs4407490	4	26515729	0.017	0.013	0.034		
rs6448619	4	28648302	0.018	0.045	0.007		
rs12641755	4	29402363	0.018	0.000	0.033		
rs2121659	4	30461576	0.036	0.084	0.031	1	1
rs6813096	4	31685155	0.031	0.052	0.001		
rs12513173	4	31791578	0.040	0.001	0.045	1	
rs9761257	4	32847612	0.019	0.020	0.039		
rs1459452	4	34367227	0.035	0.009	0.032		
rs10023150	4	34418390	0.027	0.056	0.015		
rs7695386	4	36902139	0.028	0.043	0.018		
rs7672883	4	37829252	0.058	0.000	0.007	1	
rs11096936	4	38035690	0.029	0.055	0.003		

rs6853067	4	38385260	0.020	0.000	0.037		
rs11728133	4	38532964	0.059	0.055	0.001	1	
rs2044917	4	38972057	0.058	0.089	0.000	1	1
rs2125312	4	39363978	0.058	0.037	0.014	1	
rs2381501	4	40040272	0.062	0.015	0.000	1	
rs12647748	4	40294280	0.025	0.048	0.004		
rs2575516	4	42641817	0.054	0.046	0.004		
rs6828560	4	43169635	0.062	0.000	0.025	1	
rs6828553	4	43990492	0.021	0.031	0.040		
rs1995287	4	45124794	0.028	0.050	0.012		
rs2279334	4	53573373	0.020	0.019	0.041		
rs13122232	4	53776195	0.025	0.061	0.026	1	
rs9312642	4	54296979	0.058	0.034	0.003	1	
rs7691817	4	54298789	0.033	0.008	0.033		
rs12233727	4	54914198	0.032	0.059	0.014	1	
rs3020827	4	55420009	0.031	0.074	0.019	1	1
rs6554290	4	56277958	0.016	0.020	0.034		
rs11728328	4	56827857	0.040	0.057	0.001		
rs4865075	4	56954058	0.075	0.015	0.012	1	1
rs1579019	4	57183563	0.020	0.003	0.033		
rs7680140	4	57664385	0.038	0.072	0.005	1	1
rs2333727	4	59779497	0.028	0.008	0.038		
rs1482699	4	60329474	0.040	0.043	0.000		
rs6551519	4	60812867	0.033	0.006	0.036		
rs10517498	4	61039534	0.041	0.071	0.004	1	1
rs2348698	4	65291062	0.033	0.070	0.008	1	1
rs1514269	4	65950042	0.029	0.055	0.002		
rs4425430	4	66169733	0.027	0.000	0.036		
rs2218058	4	68669310	0.024	0.045	0.041		
rs1709287	4	71194480	0.029	0.068	0.016	1	
rs1526691	4	72878213	0.030	0.047	0.009		
rs1401144	4	73871852	0.080	0.058	0.006	1	1
rs7656798	4	75753008	0.051	0.088	0.003	1	1
rs12642133	4	75900468	0.056	0.027	0.000	1	
rs2645651	4	78221667	0.023	0.024	0.040		
rs881997	4	78455985	0.044	0.068	0.017	1	
rs6836305	4	78971401	0.030	0.054	0.002		
rs4975147	4	79845449	0.044	0.087	0.012	1	1
rs2867709	4	81607620	0.029	0.000	0.032		
rs13119650	4	82151004	0.070	0.038	0.000	1	1
rs17005004	4	82199847	0.049	0.045	0.005		
rs2643925	4	82341375	0.029	0.001	0.040		
rs10033009	4	82849542	0.024	0.035	0.055	1	1
rs9307787	4	83047673	0.079	0.019	0.023	1	1
rs4693614	4	84617700	0.032	0.057	0.023		
rs11728848	4	86435862	0.060	0.002	0.050	1	
rs1563060	4	86548491	0.068	0.001	0.036	1	1
rs1462359	4	88799596	0.034	0.053	0.002		
rs10516801	4	89298728	0.045	0.014	0.037		
rs2622629	4	89451243	0.054	0.073	0.000		
rs10516817	4	89945245	0.052	0.090	0.046	1	

rs903600	4	90787873	0.039	0.008	0.036		
rs6824866	4	91666164	0.029	0.044	0.010		
rs11734837	4	93978598	0.017	0.001	0.033		
rs1821342	4	94529275	0.032	0.000	0.043	1	
rs7682651	4	96332648	0.024	0.002	0.039		
rs4699438	4	96923507	0.025	0.000	0.039		
rs17024571	4	97080375	0.062	0.077	0.002	1	
rs10488827	4	98218319	0.032	0.002	0.040		
rs2162369	4	99230845	0.026	0.002	0.036		
rs6835899	4	99816155	0.028	0.002	0.040		
rs6532774	4	100205130	0.044	0.099	0.014	1	1
rs10516441	4	100664345	0.060	0.008	0.040		
rs991811	4	100868037	0.060	0.108	0.003	1	1
rs10516451	4	100983727	0.101	0.095	0.010	1	1
rs7661798	4	101477103	0.017	0.012	0.039		
rs2695219	4	102441070	0.035	0.071	0.026	1	1
rs4647992	4	103812532	0.019	0.016	0.039		
rs1609798	4	103894643	0.041	0.070	0.000	1	1
rs11722288	4	104890554	0.073	0.081	0.000	1	1
rs7657942	4	104898564	0.043	0.091	0.032		
rs975534	4	105859891	0.029	0.048	0.001		
rs7698522	4	106476360	0.014	0.025	0.032		
rs6849573	4	107381612	0.019	0.002	0.039		
rs17483781	4	108472626	0.057	0.005	0.024	1	
rs219472	4	109669245	0.019	0.013	0.035		
rs10516554	4	110279668	0.056	0.038	0.006	1	
rs3853444	4	112091740	0.018	0.010	0.036		
rs879069	4	113737322	0.032	0.048	0.010		
rs916874	4	114749997	0.056	0.004	0.002	1	
rs11562909	4	118900647	0.029	0.045	0.000		
rs1523742	4	119556656	0.045	0.064	0.000	1	
rs13135303	4	121206922	0.041	0.000	0.051	1	1
rs903148	4	121441284	0.076	0.008	0.044	1	1
rs2390024	4	121828654	0.027	0.057	0.016		
rs1450467	4	122250095	0.026	0.000	0.032		
rs4833229	4	122958269	0.019	0.017	0.039		
rs2589858	4	124928112	0.047	0.067	0.002	1	
rs1820372	4	125026851	0.038	0.001	0.058	1	1
rs17007675	4	125233541	0.060	0.031	0.000	1	
rs508410	4	125852109	0.030	0.054	0.015		
rs7666354	4	126675973	0.057	0.089	0.036	1	
rs1156232	4	126790819	0.036	0.001	0.066	1	1
rs574805	4	127422275	0.076	0.070	0.001	1	1
rs2390992	4	127462965	0.065	0.076	0.003	1	1
rs7674389	4	128163071	0.057	0.007	0.088	1	
rs6841658	4	128747387	0.029	0.034	0.052	1	1
rs12509092	4	129401414	0.079	0.036	0.001	1	1
rs3733260	4	129565864	0.025	0.056	0.039		
rs6847753	4	130131980	0.054	0.053	0.012		
rs11933240	4	130151164	0.021	0.006	0.033		
rs337277	4	130388257	0.028	0.060	0.008	1	

rs12507354	4	131211445	0.014	0.004	0.034		
rs500131	4	132483444	0.017	0.015	0.038		
rs348071	4	133492158	0.023	0.044	0.002		
rs2248714	4	134894015	0.028	0.007	0.054	1	1
rs10013441	4	136132843	0.023	0.010	0.040		
rs11099322	4	136436598	0.034	0.060	0.002	1	
rs13113232	4	137509310	0.064	0.045	0.004	1	1
rs10519404	4	138233054	0.038	0.078	0.014	1	1
rs1398925	4	139658289	0.025	0.000	0.041		
rs7660661	4	140509341	0.068	0.015	0.017	1	1
rs11726692	4	142277642	0.024	0.053	0.004		
rs1909868	4	143193702	0.018	0.000	0.039		
rs11947381	4	145895455	0.021	0.051	0.007		
rs1992598	4	146692359	0.024	0.032	0.035		
rs7689088	4	147595857	0.036	0.000	0.039		
rs17021996	4	148112424	0.024	0.045	0.000		
rs4405992	4	151368067	0.028	0.060	0.005	1	
rs1907155	4	155718849	0.023	0.021	0.050	1	1
rs6825017	4	155992880	0.090	0.142	0.000	1	1
rs9998104	4	156527964	0.022	0.032	0.044	1	
rs10517629	4	157461554	0.032	0.059	0.005	1	
rs4324595	4	157799246	0.076	0.038	0.014	1	1
rs10027000	4	159332158	0.027	0.057	0.000		
rs1946869	4	161743988	0.034	0.067	0.028	1	
rs11100291	4	161793946	0.027	0.032	0.060	1	1
rs7684439	4	163602779	0.023	0.034	0.036		
rs10517764	4	163626661	0.056	0.000	0.002	1	
rs6536693	4	164193176	0.023	0.051	0.011		
rs11730342	4	166757343	0.041	0.069	0.016	1	1
rs4596200	4	167726417	0.024	0.056	0.005		
rs10517948	4	168899699	0.021	0.000	0.040		
rs11131751	4	168966986	0.031	0.045	0.002		
rs4373124	4	171205912	0.028	0.058	0.009	1	
rs951632	4	172355072	0.025	0.053	0.035		
rs6857801	4	172833330	0.073	0.081	0.000	1	1
rs6553726	4	175057496	0.053	0.060	0.000	1	
rs4695894	4	175192440	0.059	0.028	0.003	1	
rs7656851	4	176012620	0.030	0.012	0.037		
rs4382019	4	176322368	0.061	0.101	0.007	1	
rs12644889	4	177898122	0.040	0.050	0.051	1	1
rs1920968	4	178876442	0.021	0.008	0.034		
rs1121015	4	179102588	0.025	0.045	0.008		
rs9998128	4	180271856	0.024	0.048	0.008		
rs7655585	4	181174294	0.020	0.034	0.037		
rs1435499	4	182751506	0.062	0.001	0.036	1	
rs10520487	4	182852585	0.046	0.062	0.011	1	
rs13435909	4	185162308	0.031	0.000	0.041		
rs2309942	4	185214978	0.066	0.030	0.029	1	1
rs6846007	4	185366753	0.039	0.061	0.001	1	
rs2014472	4	185787471	0.028	0.000	0.042	1	
rs2046535	4	186445581	0.029	0.004	0.033		

rs4862633	4	187385121	0.033	0.003	0.038		
rs7700014	4	187616646	0.029	0.051	0.004		
rs10026909	4	188227050	0.024	0.000	0.040		
rs4323178	4	189470939	0.021	0.013	0.041		
rs4476671	4	189546195	0.030	0.044	0.000		
rs1551137	4	190813755	0.022	0.032	0.042	1	
rs10475459	5	989218	0.026	0.045	0.001		
rs27061	5	1415793	0.025	0.008	0.033		
rs178421	5	2339472	0.050	0.083	0.018	1	1
rs10075636	5	2532446	0.031	0.009	0.051	1	1
rs3112424	5	2535544	0.067	0.001	0.005	1	1
rs438418	5	2902436	0.066	0.067	0.004	1	
rs4242090	5	3418033	0.016	0.001	0.032		
rs1541834	5	4641493	0.036	0.020	0.053	1	1
rs10512838	5	5936115	0.018	0.010	0.035		
rs4702379	5	6696886	0.019	0.002	0.037		
rs4701750	5	6761483	0.038	0.052	0.004		
rs331413	5	8011031	0.027	0.048	0.040		
rs9313243	5	8602411	0.020	0.047	0.008		
rs268489	5	9448673	0.039	0.087	0.009	1	1
rs3110981	5	9846538	0.058	0.017	0.010	1	
rs6555623	5	9955340	0.023	0.002	0.032		
rs884355	5	10162791	0.037	0.071	0.005	1	1
rs2589661	5	10545095	0.040	0.007	0.043	1	
rs3776421	5	10763247	0.024	0.051	0.008		
rs4998086	5	11835642	0.021	0.043	0.002		
rs2061392	5	11961641	0.058	0.031	0.000	1	
rs4701996	5	13925584	0.014	0.014	0.033		
rs697546	5	14140633	0.023	0.053	0.023		
rs638191	5	17236851	0.028	0.009	0.060	1	1
rs2134242	5	17405084	0.025	0.060	0.017	1	
rs2132502	5	18712631	0.034	0.011	0.076	1	1
rs1565004	5	19609930	0.023	0.000	0.044	1	
rs1867072	5	22784364	0.021	0.008	0.032		
rs4380611	5	23385474	0.037	0.057	0.001		
rs4701487	5	25573172	0.016	0.001	0.033		
rs10473829	5	27337279	0.040	0.056	0.005		
rs1379072	5	29814905	0.023	0.010	0.038		
rs6870987	5	30119588	0.029	0.048	0.000		
rs1276198	5	30957784	0.024	0.045	0.007		
rs7705566	5	31294886	0.020	0.007	0.035		
rs403457	5	32084967	0.020	0.000	0.036		
rs2218522	5	36050356	0.019	0.000	0.035		
rs16902947	5	36344854	0.059	0.071	0.004	1	
rs665327	5	37621606	0.042	0.054	0.000		
rs7444561	5	38262303	0.025	0.043	0.014		
rs384267	5	41302975	0.038	0.065	0.005	1	
rs10064702	5	49668519	0.023	0.005	0.051	1	1
rs4507452	5	51468426	0.028	0.042	0.005		
rs6883810	5	52146916	0.054	0.052	0.012		
rs4147737	5	53000893	0.039	0.076	0.000	1	1

rs3776742	5	53233816	0.040	0.001	0.050	1	1
rs2241403	5	53863852	0.056	0.038	0.009	1	
rs10058862	5	54158338	0.040	0.096	0.018	1	1
rs149140	5	55552745	0.034	0.004	0.050	1	1
rs30365	5	55864542	0.064	0.102	0.000	1	1
rs2194193	5	57535251	0.035	0.067	0.003	1	
rs1508918	5	57804349	0.035	0.006	0.037		
rs1115728	5	58443782	0.019	0.002	0.039		
rs7728286	5	58854479	0.057	0.090	0.000	1	
rs918592	5	59733060	0.032	0.008	0.034		
rs10471540	5	61534955	0.029	0.048	0.000		
rs2112508	5	62797490	0.022	0.049	0.012		
rs13157802	5	63873027	0.036	0.000	0.032		
rs2366778	5	63968206	0.041	0.082	0.001	1	1
rs6890124	5	65946072	0.025	0.000	0.037		
rs33344	5	67874980	0.033	0.051	0.003		
rs389686	5	68490922	0.028	0.003	0.036		
rs33425	5	71928668	0.036	0.000	0.033		
rs609659	5	72447556	0.038	0.036	0.068	1	1
rs638333	5	72455023	0.061	0.089	0.037	1	
rs10515216	5	75373492	0.029	0.070	0.012	1	1
rs4704356	5	76029331	0.021	0.043	0.003		
rs2434651	5	76259121	0.056	0.037	0.010	1	
rs252751	5	77424969	0.021	0.008	0.032		
rs413921	5	78020121	0.040	0.007	0.055	1	1
rs337895	5	78257702	0.029	0.046	0.000		
rs10072008	5	79471439	0.062	0.014	0.013	1	
rs12522787	5	79562102	0.041	0.000	0.064	1	1
rs540564	5	80439148	0.020	0.007	0.038		
rs749401	5	81597125	0.081	0.111	0.004	1	1
rs1193693	5	82573075	0.029	0.007	0.039		
rs871759	5	82784704	0.035	0.078	0.008	1	1
rs1510063	5	83732185	0.035	0.055	0.000		
rs7717527	5	83892667	0.063	0.018	0.000	1	1
rs4704022	5	84305873	0.034	0.073	0.010	1	1
rs412458	5	88065383	0.045	0.002	0.034		
rs10514321	5	89503580	0.063	0.010	0.088	1	1
rs1489531	5	92262106	0.020	0.001	0.037		
rs9314117	5	93916847	0.031	0.051	0.002		
rs956274	5	94972889	0.023	0.001	0.034		
rs3822754	5	95142971	0.069	0.068	0.008	1	1
rs10476736	5	97523185	0.072	0.106	0.033	1	1
rs11242493	5	102742862	0.064	0.102	0.027	1	1
rs6596533	5	102767658	0.065	0.102	0.027	1	1
rs40054	5	103265644	0.023	0.000	0.037		
rs7443549	5	103357930	0.034	0.053	0.000		
rs296206	5	106177292	0.027	0.046	0.003		
rs7712577	5	106385872	0.017	0.020	0.034		
rs12654281	5	107068341	0.022	0.001	0.044	1	
rs1363212	5	108619178	0.040	0.075	0.001	1	1
rs1862205	5	108684534	0.020	0.043	0.032		

rs6885593	5	111015566	0.021	0.011	0.047	1	
rs2248930	5	112839018	0.038	0.046	0.006		
rs10077453	5	114608551	0.030	0.004	0.035		
rs1422289	5	115088129	0.059	0.026	0.000	1	
rs10044983	5	115113102	0.031	0.000	0.037		
rs6878801	5	116235804	0.020	0.006	0.044	1	
rs6886341	5	116525597	0.057	0.004	0.001	1	
rs2929729	5	116814112	0.025	0.060	0.021	1	
rs905831	5	118652089	0.037	0.000	0.037		
rs7734808	5	120959748	0.028	0.025	0.033		
rs3811909	5	121775133	0.024	0.056	0.005		
rs6882579	5	123174963	0.021	0.033	0.046	1	
rs393547	5	123807199	0.030	0.054	0.011		
rs1530448	5	124668490	0.034	0.004	0.058	1	1
rs6595690	5	125654877	0.030	0.065	0.034	1	
rs968355	5	126168249	0.083	0.001	0.059	1	1
rs4355569	5	128036255	0.045	0.075	0.003	1	1
rs7733814	5	131584208	0.025	0.041	0.043	1	
rs7717377	5	132519461	0.075	0.112	0.001	1	1
rs7717860	5	134218124	0.053	0.068	0.005		
rs17168353	5	134808808	0.026	0.032	0.040		
rs12520809	5	134810409	0.030	0.051	0.016		
rs916950	5	135366498	0.031	0.000	0.033		
rs2967789	5	137034740	0.068	0.101	0.009	1	1
rs7722600	5	137222661	0.071	0.101	0.004	1	1
rs643502	5	140565698	0.024	0.048	0.006		
rs32928	5	141127429	0.026	0.060	0.020	1	
rs351260	5	141290008	0.027	0.003	0.032		
rs3776346	5	142411386	0.035	0.060	0.001	1	
rs1427864	5	143017053	0.041	0.062	0.001	1	
rs309333	5	143405856	0.016	0.034	0.032		
rs168705	5	144175846	0.036	0.001	0.049	1	
rs2910272	5	144831718	0.024	0.056	0.005		
rs161041	5	146165144	0.036	0.069	0.001	1	
rs11746607	5	147131372	0.020	0.009	0.039		
rs2438490	5	148706355	0.034	0.051	0.000		
rs30884	5	149218109	0.032	0.046	0.000		
rs152266	5	150615622	0.032	0.058	0.000	1	
rs4958532	5	151752045	0.031	0.034	0.034		
rs7705865	5	151860213	0.042	0.080	0.022	1	1
rs4958379	5	153990234	0.025	0.027	0.036		
rs42673	5	154658886	0.045	0.001	0.048	1	
rs9324820	5	154793593	0.032	0.048	0.000		
rs244954	5	155268654	0.019	0.034	0.034		
rs4502803	5	155783442	0.032	0.066	0.020	1	
rs1353249	5	156049220	0.028	0.027	0.032		
rs11738432	5	157122196	0.061	0.061	0.003	1	
rs11135022	5	157343757	0.016	0.004	0.037		
rs1027355	5	157525186	0.051	0.106	0.008	1	1
rs7445443	5	158341472	0.034	0.064	0.000	1	
rs2287763	5	158878412	0.036	0.056	0.001		

rs4921508	5	158905051	0.034	0.000	0.037		
rs2431697	5	159812556	0.047	0.070	0.000	1	1
rs9784608	5	161153666	0.036	0.048	0.003		
rs2964639	5	161617516	0.056	0.003	0.057	1	
rs5022631	5	161625396	0.048	0.002	0.062	1	1
rs7704440	5	163520113	0.048	0.090	0.009	1	1
rs4563644	5	163708849	0.084	0.040	0.001	1	1
rs2431176	5	164877527	0.037	0.002	0.051	1	1
rs262730	5	165031672	0.056	0.058	0.002	1	
rs1527822	5	165656273	0.091	0.004	0.030	1	1
rs2915858	5	166475199	0.038	0.047	0.002		
rs4257797	5	166801773	0.020	0.018	0.047	1	
rs4976576	5	167518934	0.030	0.007	0.064	1	1
rs2085449	5	167818328	0.064	0.064	0.000	1	1
rs10036727	5	168112659	0.054	0.097	0.009	1	1
rs13154825	5	168417072	0.033	0.008	0.042	1	
rs2112705	5	169057056	0.063	0.040	0.038	1	1
rs888810	5	170129216	0.025	0.053	0.032		
rs750459	5	170874577	0.036	0.055	0.000		
rs11741273	5	171884740	0.042	0.086	0.012	1	1
rs6862893	5	172103875	0.072	0.026	0.024	1	1
rs1427958	5	173752129	0.035	0.059	0.000	1	
rs1387767	5	173970940	0.015	0.007	0.032		
rs6556188	5	174483536	0.021	0.044	0.027		
rs2249160	5	174488211	0.029	0.032	0.042	1	
rs251925	5	174714725	0.059	0.025	0.031	1	
rs645574	5	175040069	0.026	0.020	0.052	1	1
rs2241577	5	175707471	0.045	0.052	0.104	1	1
rs4868644	5	175879657	0.039	0.054	0.000		
rs7715618	5	177545240	0.034	0.000	0.037		
rs2961663	5	177546150	0.066	0.008	0.037	1	1
rs1390890	5	178371565	0.031	0.001	0.062	1	1
rs10077711	5	179520355	0.019	0.045	0.007		
rs4959611	6	182215	0.026	0.058	0.020	1	
rs458399	6	1154774	0.034	0.047	0.005		
rs2338	6	1518612	0.017	0.002	0.035		
rs3823267	6	1722853	0.040	0.052	0.005		
rs318489	6	2838007	0.019	0.006	0.034		
rs3799216	6	3095514	0.032	0.055	0.001		
rs9328204	6	3832764	0.026	0.017	0.037		
rs3177253	6	4078854	0.057	0.014	0.017	1	
rs17284610	6	4885122	0.031	0.070	0.005	1	1
rs629744	6	5982119	0.027	0.004	0.052	1	1
rs2050056	6	6620338	0.031	0.044	0.000		
rs2764085	6	7223328	0.039	0.078	0.017	1	1
rs632177	6	7852138	0.033	0.000	0.055	1	1
rs4246086	6	8853959	0.026	0.055	0.009		
rs480965	6	10495237	0.036	0.000	0.042	1	
rs3798703	6	10700147	0.057	0.064	0.001	1	
rs9379840	6	10964087	0.035	0.069	0.020	1	1
rs2064111	6	11424424	0.031	0.002	0.049	1	

rs219950	6	12273349	0.030	0.009	0.059	1	1
rs9367034	6	12322312	0.055	0.114	0.009	1	1
rs6908010	6	12325985	0.099	0.096	0.016	1	1
rs7738252	6	13168554	0.029	0.024	0.044	1	
rs6922822	6	13902023	0.042	0.067	0.000	1	
rs4712229	6	14331698	0.054	0.032	0.010		
rs7770731	6	14706799	0.034	0.060	0.006	1	
rs441370	6	15058499	0.066	0.015	0.001	1	1
rs1150639	6	16851755	0.027	0.061	0.007	1	
rs6910563	6	19718814	0.045	0.001	0.050	1	1
rs9465498	6	19814532	0.040	0.081	0.027	1	1
rs1459047	6	21308369	0.098	0.164	0.009	1	1
rs807835	6	21385742	0.030	0.064	0.040		
rs7745461	6	22019595	0.083	0.020	0.026	1	1
rs1935007	6	22451280	0.040	0.065	0.005	1	
rs1570086	6	22821220	0.029	0.003	0.054	1	1
rs3920500	6	23577670	0.038	0.072	0.015	1	1
rs10498716	6	23870370	0.023	0.002	0.035		
rs1054899	6	24642172	0.020	0.001	0.035		
rs7748771	6	25463078	0.048	0.052	0.000		
rs1012899	6	25713070	0.027	0.007	0.045	1	
rs806977	6	26297594	0.026	0.004	0.057	1	1
rs2498399	6	26814523	0.062	0.064	0.000	1	
rs149971	6	28090131	0.060	0.019	0.010	1	
rs393414	6	28629295	0.021	0.000	0.036		
rs442439	6	28680985	0.048	0.081	0.003	1	1
rs6904130	6	29343172	0.034	0.058	0.001	1	
rs2734985	6	29926641	0.057	0.023	0.050	1	1
rs1611710	6	29936895	0.081	0.059	0.006	1	1
rs6904029	6	30051046	0.050	0.077	0.008	1	1
rs1012411	6	30440534	0.078	0.029	0.001	1	1
rs11753326	6	31033964	0.017	0.000	0.036		
rs3094211	6	31194381	0.065	0.029	0.000	1	1
rs3778638	6	31200103	0.064	0.049	0.015		
rs2261033	6	31711570	0.070	0.011	0.031	1	1
rs1042663	6	32013109	0.055	0.122	0.032		
rs3129939	6	32444744	0.091	0.120	0.004	1	1
rs9277027	6	33106216	0.030	0.002	0.062	1	1
rs9277554	6	33163516	0.071	0.116	0.004	1	1
rs13205164	6	33987751	0.023	0.047	0.001		
rs4144258	6	37195327	0.025	0.002	0.051	1	1
rs9380645	6	37210800	0.032	0.070	0.014	1	1
rs2815090	6	39244796	0.059	0.005	0.000	1	
rs12214600	6	39376714	0.028	0.059	0.017	1	
rs9296294	6	39447539	0.014	0.006	0.034		
rs9462739	6	41788225	0.057	0.043	0.016	1	
rs4415146	6	42049958	0.014	0.015	0.033		
rs6928781	6	42797389	0.022	0.000	0.038		
rs1041524	6	44264448	0.030	0.049	0.000		
rs6458573	6	47627280	0.025	0.046	0.005		
rs9381766	6	49400996	0.024	0.004	0.057	1	1

rs1060912	6	52497670	0.044	0.051	0.005		
rs4715354	6	52816756	0.055	0.052	0.010		
rs4715382	6	53150102	0.023	0.057	0.021		
rs816385	6	54202818	0.055	0.003	0.015		
rs7771743	6	55144879	0.022	0.014	0.045	1	
rs1024195	6	56615094	0.077	0.071	0.026	1	1
rs1482455	6	64755780	0.024	0.049	0.006		
rs11753432	6	64985775	0.035	0.041	0.055	1	1
rs13197839	6	66046013	0.034	0.008	0.067	1	1
rs2245704	6	66576477	0.019	0.016	0.042	1	
rs2812152	6	67200623	0.086	0.066	0.010	1	1
rs2816903	6	68436846	0.033	0.059	0.005	1	
rs12176580	6	68620320	0.018	0.019	0.033		
rs507326	6	69621367	0.039	0.078	0.007	1	1
rs7765774	6	70579276	0.020	0.009	0.039		
rs3909058	6	71143812	0.031	0.010	0.040		
rs2494976	6	71397368	0.028	0.059	0.014	1	
rs828655	6	72113631	0.022	0.047	0.001		
rs2492970	6	73182251	0.063	0.002	0.006	1	
rs12191488	6	74322596	0.027	0.050	0.040		
rs583443	6	74347012	0.028	0.065	0.012	1	
rs2274348	6	76801018	0.022	0.045	0.005		
rs9352339	6	77215934	0.035	0.009	0.037		
rs9448057	6	78068077	0.033	0.008	0.032		
rs4055090	6	80142606	0.041	0.068	0.005	1	
rs9359435	6	81603875	0.030	0.043	0.000		
rs623879	6	83944214	0.045	0.056	0.003		
rs9341971	6	84795153	0.056	0.035	0.001	1	
rs484746	6	85894007	0.034	0.008	0.052	1	1
rs1321737	6	85923761	0.056	0.012	0.034	1	
rs9688436	6	87328443	0.015	0.008	0.038		
rs4235832	6	88629353	0.042	0.091	0.025	1	1
rs9351162	6	89229292	0.022	0.001	0.032		
rs1055403	6	90695684	0.026	0.000	0.042	1	
rs2141484	6	91531266	0.034	0.007	0.038		
rs9342313	6	92881421	0.029	0.060	0.005	1	
rs12189899	6	93987813	0.032	0.051	0.003		
rs967520	6	94057234	0.024	0.004	0.045	1	
rs10498988	6	94568251	0.034	0.062	0.001	1	
rs416366	6	95090842	0.067	0.129	0.003	1	1
rs2472888	6	96972222	0.033	0.017	0.052	1	1
rs386936	6	97264417	0.057	0.028	0.000	1	
rs2144216	6	97645378	0.018	0.023	0.043	1	
rs9387218	6	97840992	0.030	0.045	0.020		
rs6930802	6	98200747	0.025	0.009	0.059	1	1
rs210394	6	99060054	0.017	0.010	0.034		
rs4077236	6	99915068	0.043	0.053	0.003		
rs609531	6	102161603	0.033	0.051	0.001		
rs1547384	6	102398035	0.069	0.034	0.000	1	1
rs7752919	6	103125667	0.019	0.044	0.015		
rs9386402	6	104626479	0.056	0.036	0.000	1	

rs10457135	6	106379949	0.027	0.052	0.001		
rs1934593	6	106666295	0.078	0.080	0.004	1	1
rs6935583	6	107001230	0.064	0.103	0.006	1	1
rs4530901	6	107003343	0.020	0.010	0.040		
rs2500561	6	107803794	0.026	0.020	0.051	1	1
rs527609	6	108292560	0.047	0.083	0.000	1	1
rs9386796	6	109725397	0.023	0.008	0.039		
rs4245544	6	110396286	0.033	0.001	0.035		
rs9400372	6	110761298	0.023	0.051	0.007		
rs2691165	6	110990487	0.020	0.008	0.041		
rs10499076	6	114299470	0.058	0.083	0.003	1	
rs9320485	6	114642670	0.043	0.060	0.055	1	1
rs2858829	6	116875610	0.030	0.025	0.057	1	1
rs2243384	6	117784776	0.035	0.000	0.043	1	
rs9401003	6	117824996	0.039	0.052	0.006		
rs7747363	6	118728750	0.023	0.029	0.034		
rs9374847	6	120299557	0.025	0.009	0.058	1	1
rs9372587	6	120623424	0.061	0.088	0.024	1	
rs12206641	6	121481259	0.073	0.039	0.032		
rs9490140	6	121586575	0.097	0.091	0.023	1	1
rs2679648	6	122392195	0.062	0.010	0.002	1	
rs7759311	6	123223892	0.030	0.062	0.050	1	1
rs9388176	6	123419833	0.044	0.064	0.002	1	
rs670842	6	124906854	0.029	0.005	0.064	1	1
rs3806997	6	125613725	0.032	0.068	0.002	1	
rs2027033	6	127262945	0.019	0.042	0.003		
rs6925601	6	127767194	0.016	0.010	0.036		
rs270041	6	128226431	0.018	0.043	0.010		
rs529756	6	129058667	0.049	0.064	0.000	1	
rs7754832	6	129081038	0.065	0.001	0.000	1	1
rs3816665	6	129613023	0.034	0.065	0.037	1	
rs13214956	6	129930727	0.020	0.005	0.041		
rs9388732	6	130122966	0.070	0.041	0.001	1	1
rs12660061	6	130273301	0.041	0.061	0.002	1	
rs933064	6	131225371	0.025	0.023	0.037		
rs1998075	6	132602661	0.039	0.048	0.002		
rs1467404	6	133131834	0.020	0.043	0.037		
rs212788	6	133783159	0.060	0.015	0.010	1	
rs9493627	6	133831421	0.037	0.054	0.009		
rs6908581	6	134459015	0.046	0.083	0.005	1	1
rs6569926	6	134461705	0.057	0.081	0.036	1	
rs6920152	6	134989213	0.028	0.044	0.017		
rs878175	6	135601850	0.059	0.003	0.027	1	
rs12179084	6	135621590	0.048	0.003	0.033		
rs9399161	6	136122157	0.017	0.009	0.041		
rs17256861	6	137629162	0.027	0.064	0.011	1	
rs12209996	6	137914914	0.055	0.055	0.011		
rs4896283	6	137932267	0.038	0.049	0.063	1	1
rs4896370	6	138860530	0.056	0.000	0.027	1	
rs7757846	6	139688699	0.043	0.096	0.007	1	1
rs1572674	6	140305927	0.014	0.009	0.032		

rs4896618	6	143417819	0.027	0.051	0.001		
rs1107205	6	146996513	0.028	0.036	0.064	1	1
rs1147855	6	148886144	0.040	0.018	0.067	1	1
rs6570956	6	149604532	0.021	0.003	0.034		
rs742636	6	151188533	0.043	0.000	0.067	1	1
rs6917461	6	151247455	0.058	0.088	0.016	1	
rs674724	6	152716034	0.025	0.001	0.049	1	
rs1359964	6	153272210	0.024	0.017	0.052	1	1
rs1321602	6	153680372	0.034	0.060	0.006	1	
rs1022404	6	154991815	0.054	0.003	0.017		
rs2281253	6	155840325	0.019	0.021	0.042	1	
rs7743663	6	156079651	0.019	0.046	0.011		
rs9478712	6	156950597	0.040	0.073	0.005	1	1
rs10946113	6	158555100	0.063	0.082	0.001	1	1
rs9457507	6	159286839	0.021	0.049	0.011		
rs3120629	6	159485008	0.074	0.041	0.004	1	1
rs7771621	6	159897586	0.056	0.077	0.001	1	1
rs372440	6	159923558	0.030	0.000	0.056	1	1
rs3798168	6	160530218	0.046	0.044	0.000		
rs657030	6	161363722	0.038	0.014	0.048	1	
rs9365310	6	162051953	0.056	0.074	0.012	1	
rs952388	6	162686952	0.033	0.062	0.023	1	
rs11968371	6	163262364	0.035	0.017	0.062	1	1
rs1909056	6	164915751	0.030	0.045	0.001		
rs9365729	6	164960849	0.054	0.040	0.001		
rs9355566	6	166660011	0.023	0.001	0.039		
rs10484524	6	167066688	0.032	0.056	0.002		
rs9455968	6	168399918	0.051	0.059	0.000	1	
rs4708745	6	168730176	0.096	0.022	0.005	1	1
rs196489	6	168950666	0.018	0.016	0.033		
rs9383480	6	170011091	0.027	0.027	0.039		
rs7762762	6	170046330	0.027	0.062	0.002	1	
rs10275469	7	234196	0.019	0.000	0.037		
rs1476636	7	2756761	0.037	0.038	0.080	1	1
rs1636412	7	3955772	0.030	0.000	0.032		
rs314582	7	4250265	0.077	0.106	0.000	1	1
rs7808785	7	7578969	0.027	0.057	0.001		
rs7785709	7	7711584	0.064	0.036	0.000	1	1
rs11764675	7	7758473	0.034	0.009	0.034		
rs1158990	7	8710531	0.047	0.065	0.001	1	
rs6979515	7	8801078	0.018	0.030	0.032		
rs2049387	7	10232035	0.033	0.056	0.036		
rs6973809	7	10655067	0.056	0.035	0.011	1	
rs4720989	7	11292158	0.035	0.068	0.000	1	
rs2354952	7	11342122	0.025	0.006	0.055	1	1
rs2883998	7	12599638	0.037	0.068	0.006	1	
rs16877717	7	13011736	0.089	0.031	0.004	1	1
rs2723441	7	13295181	0.051	0.053	0.009		
rs7780524	7	13982379	0.040	0.080	0.030	1	1
rs2043221	7	14239132	0.014	0.008	0.033		
rs917037	7	14798572	0.030	0.010	0.042	1	

rs11984306	7	15024254	0.028	0.065	0.008	1	
rs38242	7	15711054	0.026	0.058	0.016	1	
rs1120230	7	15923857	0.024	0.043	0.042	1	
rs10486293	7	18409035	0.018	0.016	0.039		
rs10486333	7	18973372	0.062	0.003	0.018	1	
rs1023541	7	20535064	0.029	0.004	0.057	1	1
rs10270923	7	20846194	0.030	0.056	0.046		
rs2069837	7	22541267	0.022	0.000	0.042		
rs1637193	7	23033491	0.023	0.057	0.018		
rs10268610	7	23048004	0.021	0.031	0.046	1	
rs6950415	7	23714338	0.028	0.015	0.058	1	1
rs5574	7	24102373	0.028	0.060	0.020	1	
rs2525764	7	26251836	0.047	0.047	0.003		
rs10254677	7	27162438	0.018	0.017	0.044	1	
rs4722752	7	27843551	0.031	0.000	0.043	1	
rs317712	7	28875836	0.028	0.052	0.045		
rs1568658	7	28914798	0.024	0.023	0.059	1	1
rs2529447	7	30308450	0.026	0.011	0.053	1	1
rs7809542	7	30986887	0.034	0.061	0.000	1	
rs2270218	7	31650006	0.051	0.001	0.039		
rs7791492	7	32293439	0.024	0.021	0.037		
rs7799346	7	33085260	0.018	0.000	0.041		
rs10243554	7	33661735	0.043	0.005	0.048	1	
rs324383	7	34543735	0.019	0.010	0.039		
rs10275497	7	35889778	0.020	0.045	0.014		
rs4723596	7	36724284	0.021	0.006	0.037		
rs1016321	7	37068355	0.034	0.045	0.001		
rs1524050	7	37820089	0.025	0.003	0.035		
rs1019179	7	37992216	0.034	0.049	0.001		
rs4618604	7	39307014	0.019	0.043	0.021		
rs13224312	7	39461392	0.022	0.000	0.040		
rs718224	7	41086232	0.021	0.005	0.050	1	1
rs2035425	7	41351207	0.093	0.002	0.015	1	1
rs2049622	7	41949522	0.029	0.054	0.000		
rs10246620	7	43197352	0.024	0.003	0.049	1	
rs1969510	7	45164055	0.026	0.001	0.047	1	
rs2471267	7	45532502	0.031	0.066	0.007	1	
rs17172724	7	46395548	0.025	0.047	0.005		
rs983398	7	46415637	0.021	0.008	0.032		
rs17172801	7	46812503	0.056	0.007	0.034	1	
rs1438603	7	46961304	0.035	0.066	0.008	1	
rs6463394	7	46998015	0.025	0.033	0.056	1	1
rs1915960	7	47817718	0.032	0.046	0.000		
rs6949474	7	48107203	0.017	0.005	0.032		
rs10236459	7	48280553	0.061	0.000	0.018	1	
rs10487574	7	48552509	0.038	0.071	0.009	1	1
rs7780012	7	50212929	0.059	0.000	0.052	1	
rs956117	7	50499894	0.029	0.064	0.019	1	
rs7786795	7	51495767	0.058	0.018	0.002	1	
rs1526765	7	53847822	0.051	0.109	0.000	1	1
rs7803359	7	54567311	0.041	0.055	0.000		

rs9656709	7	55498654	0.038	0.063	0.000	1	
rs698626	7	56057064	0.059	0.004	0.031	1	
rs1543109	7	56506407	0.025	0.003	0.045	1	
rs10807715	7	61470122	0.019	0.044	0.005		
rs13240999	7	66830664	0.063	0.084	0.000	1	
rs6978595	7	67809896	0.021	0.020	0.051	1	1
rs2293501	7	69696574	0.023	0.050	0.026		
rs10950250	7	70131177	0.017	0.016	0.038		
rs992125	7	70677643	0.056	0.080	0.000	1	
rs2856728	7	72915433	0.029	0.057	0.003		
rs2868248	7	76154870	0.028	0.043	0.000		
rs16885938	7	77604880	0.020	0.026	0.037		
rs9649304	7	77737325	0.039	0.093	0.034	1	1
rs2074725	7	80983955	0.027	0.055	0.003		
rs1010344	7	81553044	0.032	0.025	0.070	1	1
rs10808300	7	82052795	0.027	0.042	0.016		
rs2189103	7	82560081	0.022	0.007	0.044	1	
rs10499878	7	83284338	0.028	0.045	0.002		
rs12668550	7	85321570	0.032	0.004	0.032		
rs1202186	7	86857909	0.020	0.008	0.032		
rs10487018	7	87720783	0.021	0.051	0.008		
rs1208853	7	88549325	0.017	0.000	0.033		
rs10953089	7	92373078	0.023	0.048	0.005		
rs1034620	7	93708066	0.051	0.067	0.000	1	
rs7790776	7	94128988	0.031	0.000	0.039		
rs319331	7	95189014	0.031	0.005	0.034		
rs10486028	7	95664124	0.031	0.068	0.005	1	
rs6975031	7	99384013	0.067	0.093	0.011	1	1
rs12705099	7	100163109	0.025	0.050	0.009		
rs1008064	7	100709653	0.033	0.063	0.000	1	
rs496907	7	102956435	0.038	0.056	0.002		
rs1706896	7	104850325	0.031	0.014	0.075	1	1
rs176489	7	105330922	0.028	0.062	0.030	1	
rs10953522	7	106182377	0.044	0.043	0.037		
rs1724278	7	106183181	0.054	0.083	0.000		
rs10499978	7	108801148	0.051	0.014	0.045	1	
rs1917717	7	109826515	0.073	0.051	0.000	1	1
rs2030780	7	109959163	0.024	0.001	0.035		
rs6974167	7	111353535	0.083	0.007	0.091	1	1
rs760444	7	115590807	0.024	0.051	0.001		
rs4730972	7	119901169	0.032	0.050	0.000		
rs1899689	7	121558300	0.024	0.032	0.055	1	1
rs1006091	7	122136741	0.019	0.012	0.044	1	
rs1019200	7	123489540	0.050	0.096	0.001	1	1
rs2191470	7	123493407	0.073	0.089	0.017	1	1
rs6949899	7	126739674	0.015	0.004	0.032		
rs4731416	7	127438046	0.023	0.051	0.003		
rs1806626	7	128923624	0.018	0.009	0.036		
rs2280680	7	129143119	0.066	0.125	0.002	1	1
rs901799	7	129525353	0.073	0.122	0.001	1	1
rs6954253	7	129950927	0.028	0.004	0.055	1	1

rs1426470	7	130624825	0.019	0.005	0.040		
rs10242684	7	132314965	0.018	0.025	0.042	1	
rs10488449	7	133849129	0.040	0.002	0.076	1	1
rs12540516	7	133864216	0.062	0.047	0.002	1	
rs4236657	7	134855349	0.018	0.010	0.041		
rs923336	7	135135382	0.050	0.061	0.003	1	
rs10954532	7	135644017	0.018	0.005	0.036		
rs7796493	7	135883478	0.037	0.043	0.011		
rs864434	7	136686683	0.026	0.051	0.000		
rs6467735	7	137243570	0.022	0.011	0.036		
rs1906889	7	137342436	0.028	0.054	0.002		
rs2267698	7	139069977	0.034	0.000	0.037		
rs801089	7	140104707	0.023	0.043	0.001		
rs7796556	7	141706219	0.029	0.000	0.040		
rs4595035	7	142658312	0.026	0.045	0.000		
rs2949739	7	142703490	0.021	0.000	0.035		
rs2023995	7	144428939	0.027	0.045	0.004		
rs6464815	7	146621930	0.020	0.003	0.032		
rs4501499	7	147396515	0.028	0.047	0.003		
rs9648855	7	147760233	0.022	0.006	0.041		
rs10464475	7	148491325	0.021	0.046	0.004		
rs855740	7	148805446	0.057	0.031	0.009	1	
rs2215564	7	150001612	0.028	0.043	0.005		
rs1547958	7	150077933	0.055	0.043	0.009		
rs2536085	7	150825179	0.055	0.001	0.001		
rs1523641	7	152041990	0.023	0.040	0.038		
rs4074568	7	153332837	0.022	0.050	0.005		
rs4960602	7	153945936	0.020	0.048	0.014		
rs879763	7	155222169	0.024	0.003	0.034		
rs4716952	7	155266388	0.024	0.045	0.019		
rs2001763	7	157548897	0.034	0.006	0.054	1	1
rs17812491	8	286848	0.048	0.089	0.028	1	1
rs876412	8	539870	0.027	0.038	0.036		
rs17754335	8	1237670	0.030	0.059	0.004	1	
rs2198944	8	2342522	0.022	0.045	0.035		
rs922790	8	3387027	0.018	0.006	0.043	1	
rs7815959	8	3447471	0.085	0.000	0.007	1	1
rs2624087	8	3508279	0.053	0.051	0.008		
rs2407941	8	4097308	0.021	0.002	0.047	1	
rs893465	8	4749173	0.032	0.009	0.046	1	
rs12335274	8	4856110	0.033	0.062	0.014	1	
rs6981227	8	5454564	0.056	0.042	0.000	1	
rs2214689	8	5543206	0.051	0.056	0.000		
rs2442579	8	6435457	0.032	0.062	0.014	1	
rs1914826	8	8258498	0.029	0.053	0.004		
rs885000	8	8606285	0.058	0.034	0.001	1	
rs9329172	8	8804291	0.044	0.057	0.005		
rs11787063	8	9483710	0.026	0.000	0.032		
rs10086770	8	10547921	0.028	0.043	0.009		
rs4840508	8	10600524	0.024	0.000	0.041		
rs3735814	8	11644805	0.028	0.064	0.003	1	

rs2977124	8	12817272	0.059	0.046	0.003	1	
rs1188306	8	13110780	0.038	0.071	0.000	1	1
rs10503475	8	13647847	0.037	0.077	0.049	1	
rs12680924	8	15098795	0.058	0.027	0.030	1	
rs9325747	8	15112351	0.039	0.072	0.003	1	1
rs12676548	8	16115389	0.057	0.007	0.046	1	
rs891320	8	16290025	0.027	0.057	0.020		
rs3780121	8	17462963	0.055	0.071	0.000		
rs2410501	8	17585971	0.025	0.039	0.045	1	
rs2717728	8	18454168	0.070	0.071	0.011	1	1
rs12544783	8	19074617	0.024	0.041	0.000		
rs991359	8	19129452	0.031	0.005	0.041		
rs1868662	8	20593730	0.024	0.051	0.008		
rs4739199	8	21268454	0.057	0.001	0.019	1	
rs10503707	8	21407401	0.052	0.063	0.007	1	
rs1552286	8	22020747	0.072	0.018	0.006	1	1
rs12545575	8	22330562	0.033	0.051	0.008		
rs7957	8	23049312	0.030	0.026	0.037		
rs7824768	8	23305892	0.041	0.093	0.019	1	1
rs192841	8	23681007	0.017	0.025	0.042	1	
rs2291578	8	24312524	0.021	0.052	0.010		
rs1035722	8	25689448	0.027	0.001	0.039		
rs11135938	8	26369653	0.051	0.108	0.010	1	1
rs563097	8	26773040	0.059	0.065	0.044	1	
rs573514	8	26777198	0.047	0.076	0.070	1	1
rs4515501	8	27819048	0.031	0.062	0.001	1	
rs924184	8	28352090	0.017	0.014	0.041		
rs10091038	8	29416224	0.056	0.063	0.001	1	
rs323344	8	30822067	0.040	0.000	0.037		
rs9297171	8	31298593	0.057	0.071	0.000	1	
rs7000656	8	32432396	0.036	0.074	0.014	1	1
rs12544147	8	32962597	0.064	0.000	0.022	1	1
rs10105616	8	33906372	0.035	0.080	0.010	1	1
rs7839962	8	34579656	0.035	0.054	0.002		
rs10955033	8	37765824	0.034	0.054	0.001		
rs11783920	8	41520070	0.033	0.056	0.000		
rs11785763	8	41552050	0.017	0.025	0.032		
rs5029748	8	42259706	0.021	0.042	0.001		
rs12334834	8	49445899	0.034	0.047	0.000		
rs12542203	8	49868312	0.057	0.013	0.000	1	
rs6987879	8	49970615	0.051	0.043	0.012		
rs3886248	8	53089326	0.034	0.050	0.004		
rs7822058	8	53847414	0.020	0.008	0.042	1	
rs6991827	8	53917714	0.061	0.045	0.002	1	
rs12675595	8	54330478	0.035	0.055	0.037		
rs9298497	8	55098835	0.058	0.069	0.002	1	
rs145290	8	55692165	0.054	0.067	0.006		
rs6473978	8	56311365	0.059	0.034	0.002	1	
rs2939654	8	56543364	0.026	0.044	0.003		
rs7833645	8	56645090	0.032	0.027	0.058	1	1
rs7834625	8	57930505	0.068	0.060	0.022	1	

rs918911	8	57934562	0.076	0.035	0.055	1	1
rs6471606	8	58530376	0.015	0.013	0.033		
rs17239618	8	59104017	0.064	0.116	0.002	1	1
rs6471696	8	59345986	0.025	0.040	0.037		
rs2726556	8	59955332	0.037	0.077	0.003	1	1
rs1562612	8	59979622	0.089	0.063	0.047	1	1
rs721011	8	60005056	0.040	0.052	0.074	1	1
rs1948508	8	62890692	0.030	0.064	0.001	1	
rs6472088	8	64381899	0.018	0.034	0.032		
rs1371685	8	65360294	0.020	0.043	0.004		
rs3110289	8	65509490	0.027	0.038	0.050	1	1
rs2128105	8	68682239	0.039	0.057	0.003		
rs1568828	8	69284682	0.034	0.046	0.000		
rs12542665	8	70406494	0.028	0.015	0.034		
rs7831688	8	70708773	0.036	0.074	0.005	1	1
rs6472494	8	70974604	0.029	0.038	0.032		
rs10957744	8	76061989	0.058	0.092	0.000	1	1
rs1543024	8	76165342	0.060	0.034	0.001	1	
rs13249060	8	76500610	0.027	0.039	0.039		
rs919577	8	77308136	0.035	0.007	0.032		
rs1526662	8	77410965	0.025	0.051	0.002		
rs11996335	8	77536681	0.060	0.031	0.002	1	
rs12677622	8	78144200	0.027	0.049	0.009		
rs1051919	8	81600865	0.033	0.052	0.018		
rs10957985	8	81952476	0.034	0.000	0.050	1	
rs10504737	8	82319137	0.065	0.003	0.000	1	1
rs7842063	8	83901003	0.054	0.015	0.021		
rs10958161	8	84710007	0.064	0.006	0.009	1	1
rs6473531	8	85280011	0.021	0.047	0.008		
rs1483767	8	87103841	0.028	0.061	0.014	1	
rs997597	8	88259667	0.020	0.043	0.014		
rs2915524	8	88266138	0.034	0.004	0.036		
rs7819401	8	88900831	0.020	0.005	0.046	1	
rs10090064	8	92965198	0.044	0.061	0.001	1	
rs2339657	8	93740838	0.025	0.052	0.014		
rs278606	8	94253315	0.026	0.010	0.049	1	
rs1447307	8	94355216	0.027	0.056	0.001		
rs2978133	8	95164339	0.026	0.054	0.035		
rs2613225	8	95301625	0.041	0.057	0.007		
rs3104993	8	96641338	0.059	0.070	0.000	1	
rs2013433	8	97005477	0.031	0.005	0.037		
rs7827095	8	97308302	0.038	0.069	0.000	1	
rs2319928	8	97892288	0.036	0.000	0.054	1	1
rs1835740	8	98236089	0.038	0.066	0.024	1	
rs1478947	8	98552411	0.056	0.062	0.000	1	
rs2512413	8	98652601	0.016	0.001	0.032		
rs4734429	8	100362919	0.023	0.014	0.053	1	1
rs4734021	8	102137336	0.065	0.068	0.021	1	1
rs979891	8	102526462	0.031	0.000	0.052	1	1
rs1265121	8	103259273	0.034	0.049	0.000		
rs2513927	8	103776657	0.020	0.043	0.009		

rs12541167	8	104314846	0.035	0.060	0.001	1	
rs2441851	8	105380081	0.030	0.056	0.015		
rs2166646	8	105397705	0.034	0.031	0.045	1	
rs974208	8	106190921	0.066	0.009	0.019	1	1
rs921335	8	106644508	0.039	0.065	0.003	1	
rs1901781	8	107565788	0.021	0.014	0.046	1	
rs10505105	8	108376513	0.037	0.049	0.005		
rs1513520	8	114526293	0.077	0.129	0.006	1	1
rs10505210	8	115047181	0.015	0.015	0.032		
rs10505229	8	115908561	0.026	0.045	0.002		
rs2721940	8	116705456	0.028	0.000	0.041		
rs720133	8	117985462	0.022	0.038	0.034		
rs1499439	8	118353561	0.040	0.064	0.019	1	
rs10955841	8	119015722	0.035	0.069	0.006	1	
rs9297575	8	119160349	0.056	0.018	0.014	1	
rs7005520	8	119412676	0.020	0.023	0.046	1	
rs4876418	8	119608693	0.026	0.045	0.010		
rs7016899	8	122243666	0.017	0.024	0.037		
rs10505398	8	122370337	0.033	0.057	0.017		
rs1371713	8	122849986	0.024	0.004	0.032		
rs13280282	8	123865362	0.061	0.045	0.001	1	
rs13252935	8	124202699	0.041	0.052	0.014		
rs7843017	8	124964990	0.025	0.053	0.006		
rs4871473	8	125305567	0.027	0.005	0.037		
rs4870938	8	126421004	0.023	0.002	0.041		
rs7016867	8	126663920	0.061	0.116	0.000	1	
rs283721	8	128379675	0.067	0.082	0.006	1	1
rs4733597	8	128686794	0.026	0.038	0.054	1	1
rs6470586	8	128957501	0.052	0.106	0.024	1	1
rs1516959	8	129472209	0.062	0.012	0.027	1	
rs1835756	8	129772601	0.040	0.065	0.004	1	
rs4733711	8	130614627	0.063	0.116	0.001	1	1
rs2163951	8	130672539	0.072	0.004	0.017	1	1
rs7007679	8	130773250	0.029	0.001	0.032		
rs263264	8	131905912	0.032	0.073	0.031	1	1
rs6470860	8	131974372	0.033	0.004	0.076	1	1
rs1031635	8	133320979	0.038	0.061	0.040		
rs7834336	8	133517751	0.069	0.062	0.019	1	1
rs2958692	8	134203855	0.038	0.065	0.000	1	
rs11775725	8	134635315	0.047	0.052	0.062	1	1
rs1401346	8	134667286	0.088	0.032	0.031	1	1
rs4909899	8	136273509	0.027	0.000	0.032		
rs2317571	8	136970267	0.023	0.001	0.050	1	
rs7007471	8	137280441	0.047	0.071	0.000	1	1
rs7824890	8	138375413	0.063	0.091	0.001	1	
rs6987402	8	138390253	0.032	0.003	0.033		
rs7814738	8	139246451	0.055	0.000	0.004		
rs7465307	8	139668002	0.022	0.052	0.018		
rs4736098	8	140883298	0.023	0.001	0.042	1	
rs6987480	8	141227997	0.045	0.070	0.001	1	1
rs2944765	8	141629816	0.015	0.003	0.032		

rs4917274	8	143085094	0.035	0.052	0.009		
rs4424237	8	144146656	0.025	0.049	0.020		
rs2297075	9	380512	0.030	0.017	0.057	1	1
rs12685329	9	715147	0.048	0.065	0.020	1	
rs279903	9	952184	0.084	0.041	0.040	1	1
rs2095614	9	1707960	0.016	0.005	0.037		
rs7045699	9	2218689	0.046	0.111	0.062	1	1
rs6476554	9	3637685	0.032	0.001	0.049	1	
rs627947	9	3971076	0.060	0.034	0.011	1	
rs10758590	9	4267721	0.023	0.009	0.052	1	1
rs911734	9	5352699	0.019	0.000	0.035		
rs10815211	9	5402546	0.064	0.006	0.002	1	1
rs1887089	9	7349096	0.055	0.075	0.005		
rs1039336	9	8366287	0.036	0.085	0.010	1	1
rs10739169	9	8774446	0.024	0.005	0.053	1	1
rs10511511	9	8890067	0.036	0.080	0.007	1	1
rs7863177	9	9253980	0.068	0.000	0.005	1	1
rs768225	9	9760741	0.022	0.051	0.003		
rs1359095	9	10266100	0.062	0.001	0.003	1	
rs10511554	9	10743557	0.048	0.050	0.000		
rs970360	9	11438094	0.063	0.018	0.013	1	1
rs1596341	9	11489438	0.036	0.051	0.033		
rs10124285	9	11541941	0.018	0.003	0.042	1	
rs1538515	9	12257378	0.017	0.006	0.032		
rs10809808	9	12614463	0.062	0.075	0.001	1	
rs894822	9	13799735	0.025	0.045	0.001		
rs13298062	9	13931513	0.026	0.000	0.032		
rs1556029	9	14552630	0.044	0.008	0.067	1	1
rs10810269	9	14834777	0.028	0.068	0.018	1	
rs10511609	9	15325996	0.021	0.025	0.045	1	
rs3927536	9	16777670	0.032	0.000	0.040		
rs2383024	9	17477945	0.025	0.011	0.038		
rs569644	9	18101646	0.014	0.002	0.033		
rs7024439	9	18267618	0.029	0.046	0.011		
rs10122919	9	18822027	0.031	0.001	0.033		
rs7865226	9	19460631	0.031	0.054	0.022		
rs12000054	9	19703500	0.044	0.016	0.046	1	
rs2780841	9	20602516	0.106	0.127	0.003	1	1
rs4636294	9	21737803	0.072	0.020	0.053	1	1
rs1463006	9	22907331	0.034	0.005	0.055	1	1
rs4977824	9	23111911	0.028	0.051	0.001		
rs515951	9	23887432	0.047	0.089	0.033		
rs1926677	9	24784512	0.061	0.021	0.027	1	
rs7848925	9	25349813	0.054	0.037	0.009		
rs1231360	9	25740661	0.029	0.000	0.040		
rs1434882	9	26197358	0.055	0.084	0.001		
rs1889899	9	26779940	0.036	0.078	0.051	1	1
rs2123204	9	28048435	0.029	0.066	0.019	1	
rs2026372	9	28420173	0.028	0.043	0.057	1	1
rs1041683	9	29843258	0.016	0.007	0.040		
rs1333616	9	31227357	0.019	0.001	0.037		

rs1856193	9	33620008	0.071	0.077	0.008	1	1
rs7871764	9	34061541	0.040	0.004	0.062	1	1
rs11788985	9	35454754	0.018	0.002	0.036		
rs867194	9	35695086	0.026	0.048	0.005		
rs10973250	9	37188176	0.024	0.000	0.048	1	
rs7021824	9	37640203	0.029	0.043	0.000		
rs10973827	9	38435998	0.027	0.021	0.053	1	1
rs1330327	9	69392942	0.032	0.057	0.007		
rs1927099	9	70065129	0.027	0.041	0.028		
rs915490	9	70295281	0.041	0.010	0.070	1	1
rs2274699	9	71579586	0.029	0.000	0.037		
rs1567698	9	73330380	0.057	0.091	0.036	1	
rs10869430	9	74465817	0.031	0.076	0.022	1	1
rs2377527	9	76020743	0.070	0.007	0.047	1	1
rs3739524	9	76549649	0.061	0.133	0.023	1	1
rs12003722	9	76683907	0.016	0.009	0.035		
rs944558	9	76755152	0.063	0.052	0.004	1	1
rs7041310	9	77208454	0.039	0.068	0.055	1	1
rs10870022	9	77913406	0.028	0.046	0.001		
rs2482471	9	78013043	0.021	0.028	0.032		
rs4410986	9	78630440	0.023	0.017	0.042	1	
rs1688964	9	79140621	0.059	0.071	0.004	1	
rs6559541	9	80146547	0.072	0.088	0.005	1	1
rs1889065	9	80834585	0.026	0.010	0.043	1	
rs7872891	9	83309167	0.038	0.015	0.044	1	
rs7042038	9	84924270	0.032	0.064	0.002	1	
rs11140865	9	84965139	0.019	0.005	0.040		
rs4559324	9	86249869	0.054	0.024	0.002		
rs4877980	9	86275836	0.022	0.034	0.047	1	
rs10514832	9	86597888	0.036	0.064	0.001	1	
rs1930061	9	87234166	0.108	0.192	0.011	1	1
rs1571515	9	87406806	0.030	0.014	0.041		
rs7870611	9	88135219	0.038	0.000	0.042	1	
rs1556384	9	88993681	0.020	0.008	0.032		
rs4077800	9	89527722	0.024	0.008	0.037		
rs1754073	9	90113478	0.039	0.009	0.040		
rs690567	9	90433541	0.050	0.104	0.014	1	1
rs290986	9	90643091	0.063	0.004	0.055	1	1
rs7856591	9	91361054	0.020	0.016	0.037		
rs7848472	9	92277026	0.066	0.039	0.021	1	1
rs1980705	9	92453923	0.043	0.001	0.046	1	
rs12338859	9	94743175	0.026	0.055	0.003		
rs357528	9	95247137	0.017	0.001	0.038		
rs6478302	9	95471895	0.020	0.043	0.007		
rs913911	9	97398013	0.049	0.085	0.003	1	1
rs1059273	9	97929055	0.062	0.068	0.003	1	
rs10760233	9	98114181	0.034	0.020	0.060	1	1
rs4742738	9	98591695	0.024	0.047	0.015		
rs446207	9	99156963	0.064	0.039	0.004	1	1
rs2771064	9	101000133	0.071	0.075	0.005	1	1
rs1323413	9	101506279	0.040	0.006	0.035		

rs1364820	9	102223300	0.022	0.000	0.032		
rs1448577	9	103063335	0.049	0.076	0.000	1	1
rs303549	9	103118948	0.029	0.004	0.049	1	
rs1842565	9	103160934	0.071	0.057	0.012	1	1
rs535966	9	103822347	0.030	0.059	0.039		
rs4149313	9	104666308	0.018	0.016	0.037		
rs2066717	9	104671033	0.062	0.016	0.019	1	
rs1039406	9	104828513	0.040	0.071	0.011	1	1
rs10816332	9	105625993	0.055	0.016	0.001		
rs2417658	9	105753947	0.049	0.045	0.000		
rs4255200	9	105872984	0.025	0.017	0.057	1	1
rs4978688	9	107030714	0.049	0.043	0.000		
rs10816494	9	107191745	0.037	0.003	0.039		
rs817870	9	107224243	0.058	0.000	0.018	1	
rs7852099	9	107716987	0.040	0.079	0.013	1	1
rs10759317	9	108641599	0.032	0.068	0.017	1	
rs10512395	9	109322876	0.024	0.017	0.032		
rs2769151	9	109688265	0.039	0.053	0.003		
rs2017392	9	109900175	0.018	0.001	0.035		
rs952950	9	110203426	0.043	0.092	0.003	1	1
rs3739707	9	110872261	0.025	0.059	0.015	1	
rs10980811	9	111012761	0.023	0.019	0.043	1	
rs10817197	9	111461999	0.056	0.109	0.013	1	
rs2127744	9	113470623	0.034	0.048	0.000		
rs1249710	9	114089959	0.023	0.045	0.011		
rs7032231	9	114105325	0.057	0.023	0.050	1	
rs1490742	9	114114303	0.057	0.005	0.070	1	1
rs3181360	9	114771112	0.045	0.064	0.009	1	
rs16932027	9	114833660	0.025	0.000	0.034		
rs2809380	9	117931133	0.023	0.032	0.032		
rs230089	9	119035630	0.024	0.045	0.001		
rs1889274	9	119650882	0.064	0.008	0.000	1	1
rs12344392	9	119870690	0.038	0.066	0.003	1	
rs7036213	9	120094770	0.020	0.004	0.040		
rs1476860	9	122470795	0.033	0.059	0.000	1	
rs7859831	9	123285308	0.025	0.054	0.004		
rs1409897	9	123926288	0.029	0.050	0.003		
rs10819296	9	127315451	0.046	0.043	0.003		
rs10819490	9	129135872	0.078	0.064	0.024	1	1
rs913771	9	129164439	0.040	0.084	0.012	1	1
rs3808810	9	130938307	0.030	0.008	0.047	1	
rs2282004	9	131516930	0.032	0.021	0.040		
rs1412076	9	132062444	0.017	0.016	0.032		
rs2073819	9	132893744	0.024	0.000	0.043	1	
rs425850	9	133873528	0.040	0.007	0.035		
rs7867061	9	134338708	0.034	0.054	0.006		
rs3128570	9	134969254	0.027	0.045	0.001		
rs10858396	9	135501721	0.016	0.019	0.035		
rs4880807	10	1296745	0.027	0.001	0.034		
rs10903497	10	1614900	0.029	0.046	0.002		
rs1000416	10	2246731	0.029	0.068	0.004	1	

rs7080980	10	2999063	0.021	0.017	0.032		
rs6601758	10	3804773	0.066	0.009	0.000	1	1
rs12217962	10	3944953	0.030	0.062	0.014	1	
rs2398152	10	5284191	0.033	0.052	0.000		
rs6602236	10	5694173	0.019	0.008	0.044	1	
rs1129614	10	5848092	0.031	0.054	0.015		
rs7907053	10	5966160	0.061	0.043	0.006	1	
rs7916923	10	6326845	0.035	0.006	0.034		
rs11592599	10	7389950	0.027	0.041	0.044	1	
rs7077972	10	7520698	0.034	0.074	0.004	1	1
rs6602311	10	8548252	0.068	0.053	0.001	1	1
rs2388741	10	8593450	0.025	0.015	0.060	1	1
rs6602325	10	8623811	0.028	0.054	0.000		
rs1149986	10	9389527	0.022	0.049	0.004		
rs2431621	10	12914721	0.063	0.077	0.001	1	1
rs472806	10	13334317	0.036	0.017	0.057	1	1
rs1881586	10	13959631	0.054	0.108	0.002		
rs3740120	10	14642449	0.031	0.034	0.066	1	1
rs7904177	10	14658852	0.038	0.077	0.006	1	1
rs7923056	10	15553996	0.057	0.077	0.003	1	
rs7091294	10	15839648	0.026	0.035	0.049	1	
rs4748282	10	16440078	0.068	0.080	0.002	1	1
rs7919985	10	16560790	0.048	0.000	0.039		
rs10508531	10	17596382	0.041	0.048	0.000		
rs2437271	10	18316767	0.023	0.023	0.055	1	1
rs12767511	10	18425190	0.031	0.051	0.000		
rs10829239	10	19023726	0.066	0.031	0.039	1	1
rs6482067	10	20175733	0.020	0.048	0.020		
rs1326236	10	20244634	0.022	0.022	0.052	1	1
rs10828064	10	20823719	0.045	0.008	0.057	1	1
rs3818673	10	24823390	0.054	0.058	0.008		
rs12354674	10	26885205	0.033	0.025	0.052	1	1
rs2274636	10	27483018	0.025	0.050	0.007		
rs10829219	10	27600126	0.015	0.012	0.035		
rs1418759	10	28573724	0.021	0.046	0.002		
rs787116	10	29074879	0.026	0.000	0.032		
rs1328325	10	29885898	0.037	0.064	0.000	1	
rs1148214	10	29999771	0.024	0.027	0.044	1	
rs7067585	10	30882797	0.034	0.042	0.002		
rs722988	10	33466153	0.021	0.009	0.035		
rs869636	10	33612045	0.054	0.046	0.002		
rs2749601	10	34215191	0.021	0.008	0.039		
rs10764019	10	35295871	0.016	0.004	0.032		
rs7099606	10	36215246	0.044	0.054	0.011		
rs1219615	10	36630158	0.053	0.012	0.008		
rs1933748	10	37538233	0.025	0.031	0.040		
rs661697	10	44060704	0.014	0.008	0.032		
rs11593585	10	44391199	0.064	0.069	0.003	1	1
rs3851262	10	44978084	0.027	0.017	0.055	1	1
rs12259267	10	49184177	0.034	0.052	0.000		
rs1002196	10	49530046	0.078	0.044	0.048	1	1

rs10857437	10	50051927	0.032	0.043	0.004		
rs1258326	10	50579270	0.035	0.061	0.001	1	
rs2177371	10	51758739	0.057	0.009	0.009	1	
rs293228	10	52826465	0.065	0.013	0.049	1	1
rs14168	10	53125568	0.057	0.014	0.064	1	1
rs10824201	10	53604290	0.030	0.065	0.006	1	
rs2840323	10	54387029	0.018	0.014	0.039		
rs6481026	10	55041794	0.034	0.056	0.000		
rs1930158	10	56007561	0.032	0.061	0.003	1	
rs2488827	10	56804994	0.021	0.008	0.032		
rs7916845	10	58138173	0.066	0.074	0.003	1	1
rs10509055	10	58142473	0.038	0.089	0.004	1	1
rs1717532	10	58687032	0.020	0.043	0.001		
rs919827	10	60555306	0.032	0.066	0.007	1	
rs9804190	10	61509837	0.038	0.043	0.047	1	
rs2288358	10	61610905	0.032	0.071	0.019	1	1
rs2393638	10	61929230	0.059	0.047	0.008	1	
rs7911472	10	62539986	0.026	0.053	0.009		
rs4745960	10	64387046	0.039	0.080	0.004	1	1
rs10509178	10	64395661	0.066	0.017	0.024	1	1
rs938036	10	65328750	0.025	0.025	0.032		
rs10491072	10	65487798	0.032	0.057	0.000		
rs1509937	10	66725911	0.032	0.053	0.002		
rs10509234	10	67143979	0.022	0.016	0.039		
rs6480123	10	67417750	0.060	0.010	0.005	1	
rs1948946	10	67838960	0.060	0.085	0.001	1	1
rs1911355	10	67948325	0.067	0.022	0.006	1	1
rs2394319	10	68464762	0.031	0.001	0.056	1	1
rs7081820	10	69584015	0.033	0.003	0.037		
rs3758626	10	69895510	0.061	0.011	0.004	1	
rs12249735	10	70461241	0.026	0.000	0.032		
rs2394525	10	70482414	0.042	0.074	0.002	1	1
rs10509320	10	71021017	0.033	0.052	0.027		
rs4746906	10	71185736	0.031	0.034	0.042	1	
rs7914787	10	71532660	0.033	0.056	0.000		
rs4747001	10	71758852	0.035	0.007	0.040		
rs10999920	10	73056033	0.019	0.003	0.039		
rs3012053	10	76963115	0.023	0.007	0.055	1	1
rs10824423	10	77970548	0.020	0.017	0.047	1	
rs499437	10	78663949	0.041	0.087	0.011	1	1
rs7908220	10	79725576	0.057	0.020	0.022	1	
rs10762795	10	80075943	0.021	0.004	0.046	1	
rs7904670	10	80208866	0.024	0.049	0.005		
rs6586155	10	82461812	0.022	0.045	0.002		
rs9332404	10	83263295	0.030	0.064	0.010	1	
rs7897955	10	83505858	0.037	0.000	0.043	1	
rs10509452	10	84035623	0.031	0.075	0.032		
rs1041626	10	85448683	0.033	0.057	0.001		
rs2488624	10	86332002	0.024	0.015	0.044	1	
rs11595188	10	87896816	0.025	0.001	0.035		
rs2814352	10	88004329	0.057	0.083	0.003	1	

rs4934423	10	90575118	0.042	0.073	0.002	1	1
rs1408583	10	91607736	0.058	0.060	0.011	1	
rs1418213	10	92491630	0.045	0.080	0.002	1	1
rs10785985	10	92813519	0.016	0.000	0.032		
rs7910977	10	94199856	0.025	0.002	0.033		
rs1111875	10	94452862	0.046	0.070	0.001	1	1
rs505010	10	98511506	0.057	0.071	0.000	1	
rs1172377	10	98536450	0.041	0.000	0.055	1	1
rs6584185	10	100077218	0.022	0.055	0.009		
rs11189572	10	100108255	0.031	0.021	0.033		
rs10786489	10	100694587	0.027	0.046	0.028		
rs4491154	10	100805312	0.031	0.001	0.033		
rs284859	10	104563007	0.034	0.000	0.043	1	
rs6584555	10	105289601	0.052	0.117	0.024	1	1
rs11191727	10	105312363	0.074	0.117	0.008	1	1
rs805722	10	105800390	0.036	0.069	0.026	1	1
rs10883978	10	105940506	0.055	0.068	0.017	1	
rs703343	10	105979654	0.037	0.069	0.034		
rs10786848	10	106925199	0.032	0.048	0.005		
rs876183	10	107467567	0.029	0.018	0.067	1	1
rs11193900	10	109873649	0.031	0.008	0.062	1	1
rs10884707	10	110959073	0.039	0.053	0.022		
rs483685	10	111539386	0.017	0.003	0.036		
rs11195191	10	112325746	0.048	0.045	0.002		
rs2419577	10	112481610	0.021	0.010	0.045	1	
rs1878247	10	113308454	0.022	0.045	0.008		
rs2001808	10	113934403	0.039	0.076	0.043	1	
rs12573128	10	114720787	0.058	0.013	0.018	1	
rs1362943	10	114878598	0.026	0.008	0.052	1	1
rs9325542	10	114967359	0.032	0.057	0.005		
rs1413620	10	116082745	0.025	0.056	0.031		
rs4751940	10	117703211	0.022	0.041	0.001		
rs2420242	10	117900746	0.065	0.016	0.041	1	1
rs181586	10	119258198	0.056	0.074	0.020	1	
rs2296546	10	120474116	0.036	0.057	0.007		
rs1929681	10	122012168	0.022	0.046	0.015		
rs2420724	10	122288020	0.024	0.001	0.045	1	
rs3011369	10	122545836	0.035	0.055	0.000		
rs2981428	10	123319419	0.031	0.009	0.043	1	
rs17104498	10	124786160	0.022	0.045	0.002		
rs7909932	10	127390423	0.039	0.072	0.032	1	1
rs7070013	10	127616692	0.017	0.005	0.039		
rs4962566	10	128193535	0.038	0.053	0.001		
rs2186047	10	129367818	0.053	0.065	0.002		
rs1001990	10	130298844	0.016	0.012	0.034		
rs4750722	10	130594317	0.030	0.054	0.003		
rs7911165	10	131516640	0.023	0.030	0.032		
rs11597160	10	132163778	0.015	0.003	0.033		
rs2397395	10	132617591	0.039	0.053	0.008		
rs10830037	10	133247503	0.060	0.002	0.018	1	
rs10734456	11	1563922	0.048	0.088	0.019	1	1

rs12271352	11	4634804	0.020	0.043	0.014		
rs11036815	11	5301257	0.033	0.052	0.003		
rs2723636	11	6426633	0.021	0.017	0.032		
rs7943470	11	7483685	0.036	0.074	0.004	1	1
rs110419	11	8209429	0.026	0.049	0.006		
rs7116459	11	9232474	0.018	0.043	0.012		
rs4909926	11	10373830	0.023	0.053	0.017		
rs11021927	11	11533125	0.036	0.083	0.002	1	1
rs1471014	11	11697651	0.036	0.068	0.041		
rs4471398	11	12548352	0.102	0.101	0.001	1	1
rs7124295	11	12576495	0.020	0.043	0.037		
rs17418644	11	13098656	0.040	0.060	0.011	1	
rs1919295	11	14027143	0.019	0.021	0.032		
rs4757292	11	15120693	0.047	0.083	0.001	1	1
rs7932410	11	15355177	0.035	0.013	0.065	1	1
rs11023692	11	15704865	0.032	0.068	0.012	1	
rs11024358	11	17624137	0.033	0.010	0.035		
rs6486406	11	18062447	0.029	0.056	0.027		
rs1648222	11	19269592	0.035	0.048	0.060	1	1
rs2028570	11	20061245	0.044	0.105	0.036		
rs10833367	11	20598084	0.020	0.010	0.049	1	
rs1519717	11	20658610	0.040	0.048	0.001		
rs327036	11	20742093	0.065	0.004	0.010	1	1
rs11026033	11	21319340	0.030	0.000	0.037		
rs2665691	11	22327832	0.016	0.017	0.032		
rs1155331	11	22342766	0.023	0.042	0.009		
rs10500975	11	24291855	0.035	0.045	0.001		
rs10767218	11	24570362	0.026	0.017	0.045	1	
rs2059541	11	25273068	0.019	0.040	0.035		
rs10501061	11	26641729	0.062	0.078	0.004	1	
rs11029691	11	26717320	0.044	0.089	0.008	1	1
rs1372902	11	27090462	0.035	0.008	0.052	1	1
rs11030014	11	27437403	0.032	0.042	0.002		
rs7941325	11	28422659	0.023	0.051	0.001		
rs4520584	11	28568968	0.016	0.010	0.032		
rs10768015	11	33633185	0.021	0.021	0.043	1	
rs353615	11	35134055	0.028	0.055	0.008		
rs10768140	11	35467483	0.044	0.000	0.053	1	1
rs2289989	11	36246854	0.038	0.048	0.001		
rs10501151	11	36333179	0.058	0.003	0.032	1	
rs1381161	11	37083646	0.025	0.043	0.000		
rs10768256	11	37162511	0.030	0.000	0.033		
rs12788223	11	38363752	0.033	0.000	0.042	1	
rs11035204	11	39255833	0.049	0.000	0.062	1	1
rs1874291	11	40521785	0.027	0.057	0.000		
rs2956782	11	41391613	0.058	0.123	0.008	1	
rs178512	11	44043887	0.043	0.089	0.032		
rs16938094	11	44805851	0.038	0.018	0.039		
rs1488674	11	45530373	0.030	0.048	0.058	1	1
rs7103380	11	46166835	0.034	0.052	0.001		
rs1018136	11	48198397	0.055	0.008	0.003		

rs504661	11	55069259	0.018	0.043	0.007		
rs567766	11	56708341	0.032	0.010	0.054	1	1
rs1892964	11	56797296	0.031	0.065	0.011	1	
rs6591507	11	58699424	0.031	0.050	0.000		
rs2165832	11	59863681	0.026	0.046	0.009		
rs1941031	11	59992259	0.025	0.002	0.032		
rs174570	11	61353788	0.124	0.155	0.000	1	1
rs1675102	11	61710585	0.027	0.039	0.050	1	1
rs10501391	11	62595087	0.019	0.002	0.039		
rs2511439	11	67610611	0.018	0.044	0.004		
rs7119726	11	70136496	0.032	0.056	0.039		
rs4539352	11	74084917	0.025	0.001	0.037		
rs633473	11	74930524	0.034	0.070	0.003	1	1
rs11236720	11	75731175	0.020	0.000	0.032		
rs921383	11	77388489	0.024	0.027	0.049	1	
rs7927923	11	77657062	0.040	0.042	0.000		
rs2019214	11	78412824	0.027	0.062	0.004	1	
rs4944231	11	78937424	0.070	0.020	0.010	1	1
rs7940615	11	79416743	0.036	0.043	0.000		
rs11232234	11	79975026	0.025	0.045	0.013		
rs4945440	11	80211127	0.025	0.004	0.048	1	
rs7109922	11	80696335	0.059	0.002	0.008	1	
rs976928	11	81156691	0.024	0.026	0.034		
rs666649	11	81460553	0.029	0.058	0.017	1	
rs2037197	11	83246813	0.022	0.034	0.032		
rs11234096	11	83994094	0.032	0.034	0.077	1	1
rs7118119	11	84465202	0.029	0.045	0.003		
rs938727	11	84603291	0.022	0.013	0.033		
rs12226269	11	85873866	0.034	0.084	0.019	1	1
rs11234958	11	86466364	0.056	0.014	0.032	1	
rs301603	11	86652224	0.032	0.001	0.037		
rs7483764	11	88023261	0.052	0.076	0.006	1	1
rs6483387	11	88079083	0.056	0.030	0.000	1	
rs10830200	11	88385087	0.046	0.000	0.056	1	1
rs520751	11	89816605	0.042	0.056	0.008		
rs4483549	11	90595620	0.023	0.008	0.040		
rs1187154	11	91681501	0.023	0.000	0.040		
rs4753485	11	93013924	0.034	0.044	0.069	1	1
rs7130432	11	94035144	0.021	0.043	0.037		
rs526108	11	94793821	0.029	0.045	0.044	1	
rs728333	11	95329611	0.073	0.087	0.001	1	1
rs1893057	11	95341243	0.088	0.087	0.002	1	1
rs2298487	11	95447186	0.034	0.007	0.057	1	1
rs632374	11	96331679	0.020	0.022	0.040		
rs7930825	11	98736947	0.025	0.058	0.022	1	
rs655922	11	100153283	0.024	0.025	0.047	1	
rs7948940	11	100816569	0.031	0.034	0.072	1	1
rs2604532	11	101655698	0.063	0.038	0.018	1	
rs10501998	11	101670531	0.059	0.054	0.018		
rs470215	11	102166309	0.029	0.001	0.032		
rs1471602	11	102913537	0.038	0.068	0.014	1	

rs2047698	11	103349543	0.024	0.001	0.044	1	
rs7119086	11	103723263	0.053	0.083	0.002	1	1
rs1942510	11	104002817	0.055	0.046	0.000		
rs648762	11	105303986	0.022	0.043	0.004		
rs1791459	11	105925316	0.054	0.019	0.007		
rs3741060	11	107885191	0.056	0.033	0.002	1	
rs1789374	11	109411408	0.019	0.046	0.006		
rs12420766	11	109709110	0.038	0.010	0.032		
rs321125	11	112093919	0.027	0.057	0.021		
rs2604296	11	113764863	0.055	0.068	0.003		
rs4938144	11	114223280	0.042	0.008	0.052	1	1
rs11601059	11	115889721	0.058	0.004	0.011	1	
rs11216046	11	115997911	0.017	0.015	0.038		
rs563828	11	116726380	0.019	0.024	0.032		
rs2155195	11	117286828	0.025	0.036	0.033		
rs1894177	11	117483474	0.058	0.089	0.002	1	1
rs3741311	11	117494764	0.061	0.077	0.001	1	
rs480958	11	118083200	0.033	0.069	0.008	1	
rs7121784	11	119664262	0.030	0.052	0.014		
rs876501	11	120713908	0.055	0.000	0.002		
rs1699102	11	120962172	0.028	0.000	0.045	1	
rs549180	11	121568793	0.072	0.077	0.018	1	1
rs11605702	11	122416301	0.017	0.025	0.037		
rs513136	11	122595165	0.031	0.074	0.012	1	1
rs1453654	11	123369426	0.022	0.045	0.003		
rs2186888	11	124472984	0.021	0.028	0.032		
rs2708507	11	126597228	0.058	0.047	0.001	1	
rs675308	11	128144986	0.032	0.028	0.039		
rs3758770	11	128178036	0.039	0.083	0.014	1	1
rs752450	11	128772691	0.056	0.007	0.002	1	
rs4075242	11	130025759	0.019	0.005	0.039		
rs1448361	11	130766852	0.029	0.061	0.037	1	
rs1448354	11	130801775	0.027	0.008	0.052	1	1
rs2105606	11	131337414	0.030	0.017	0.037		
rs2163561	11	132531564	0.035	0.077	0.031	1	1
rs7107264	11	132803817	0.071	0.036	0.006	1	1
rs4937787	11	132880858	0.051	0.022	0.041		
rs12279437	11	133108704	0.059	0.105	0.003	1	1
rs1784391	11	133431914	0.033	0.010	0.044	1	
rs2119297	11	133742830	0.040	0.056	0.000		
rs2846186	11	134203586	0.029	0.051	0.040		
rs735295	12	417944	0.027	0.017	0.064	1	1
rs11831085	12	2385119	0.023	0.052	0.018		
rs1860094	12	2457579	0.019	0.014	0.042	1	
rs727864	12	3286660	0.024	0.053	0.003		
rs2878579	12	3462193	0.027	0.019	0.057	1	1
rs3217863	12	4269757	0.024	0.055	0.021		
rs10492185	12	5552427	0.031	0.035	0.077	1	1
rs1860545	12	6317038	0.017	0.014	0.037		
rs2707209	12	6775525	0.041	0.080	0.000	1	1
rs7975829	12	7960148	0.020	0.044	0.001		

rs7979266	12	10124344	0.036	0.005	0.033		
rs10772493	12	11679189	0.041	0.007	0.045	1	
rs11054813	12	12371355	0.033	0.045	0.006		
rs892267	12	13431489	0.035	0.005	0.044	1	
rs17219978	12	13541930	0.039	0.081	0.000	1	1
rs7980066	12	14551761	0.040	0.070	0.000	1	1
rs1861594	12	15552237	0.062	0.045	0.006	1	
rs10846255	12	15985428	0.032	0.044	0.000		
rs726206	12	16541935	0.038	0.070	0.012	1	1
rs7301074	12	16565898	0.021	0.019	0.048	1	
rs2971177	12	17396780	0.020	0.042	0.001		
rs1471891	12	18742801	0.020	0.001	0.037		
rs6487006	12	19713504	0.030	0.044	0.035		
rs17316054	12	20302786	0.030	0.043	0.009		
rs1910186	12	21065184	0.016	0.017	0.037		
rs1914361	12	21937120	0.026	0.049	0.003		
rs3847888	12	23025546	0.025	0.045	0.003		
rs7132369	12	23916968	0.023	0.051	0.003		
rs11047263	12	24153453	0.022	0.004	0.039		
rs11047806	12	25112841	0.025	0.009	0.039		
rs4963884	12	25457779	0.020	0.045	0.007		
rs11048246	12	25836643	0.026	0.019	0.051	1	1
rs1871554	12	26231325	0.024	0.048	0.017		
rs12422850	12	26432518	0.033	0.022	0.032		
rs412978	12	27532740	0.026	0.029	0.047	1	
rs306665	12	27590587	0.072	0.108	0.000	1	1
rs4931161	12	29230133	0.030	0.014	0.042	1	
rs12821923	12	29236581	0.054	0.086	0.002		
rs7342346	12	31965665	0.023	0.000	0.037		
rs1260003	12	31977828	0.028	0.056	0.021		
rs1384601	12	33606133	0.026	0.000	0.046	1	
rs7300022	12	37854158	0.016	0.017	0.037		
rs4491316	12	38489316	0.055	0.081	0.000	1	
rs4768245	12	39063514	0.026	0.040	0.053	1	1
rs10880088	12	40192164	0.040	0.057	0.005		
rs4288814	12	41353718	0.019	0.003	0.032		
rs7966374	12	44255693	0.024	0.040	0.049	1	
rs1049358	12	44863338	0.026	0.002	0.049	1	
rs10783196	12	46184941	0.029	0.050	0.005		
rs1544410	12	46526102	0.023	0.004	0.039		
rs584878	12	48610834	0.039	0.002	0.062	1	1
rs7131715	12	48693553	0.028	0.045	0.002		
rs764606	12	49599798	0.038	0.051	0.002		
rs12369067	12	50088923	0.019	0.011	0.042	1	
rs4761901	12	51153892	0.027	0.000	0.050	1	1
rs949734	12	51660229	0.019	0.008	0.037		
rs7969151	12	52445544	0.023	0.001	0.034		
rs754133	12	52705187	0.022	0.045	0.001		
rs2887909	12	53558451	0.025	0.020	0.055	1	1
rs324015	12	55776367	0.026	0.044	0.000		
rs2292657	12	56146199	0.030	0.021	0.062	1	1

rs1908537	12	57125093	0.034	0.004	0.046	1	
rs2712502	12	57431189	0.026	0.045	0.000		
rs1105925	12	58756300	0.031	0.056	0.008		
rs1021885	12	58994542	0.065	0.049	0.008	1	1
rs4763158	12	60513873	0.024	0.000	0.038		
rs3951389	12	61378608	0.037	0.005	0.040		
rs12581172	12	61394033	0.032	0.065	0.005	1	
rs1909327	12	61960235	0.030	0.049	0.002		
rs508792	12	63368597	0.025	0.047	0.000		
rs4237904	12	64253454	0.020	0.043	0.004		
rs2358939	12	64393114	0.028	0.001	0.033		
rs11176203	12	65146355	0.029	0.005	0.033		
rs11838180	12	65198038	0.028	0.050	0.000		
rs4913249	12	66126255	0.066	0.069	0.004	1	
rs2870805	12	66200187	0.074	0.046	0.001	1	1
rs2620777	12	66267269	0.021	0.022	0.041		
rs2193045	12	66820787	0.058	0.077	0.009	1	
rs7131697	12	67044106	0.029	0.000	0.049	1	
rs12810179	12	67458318	0.065	0.121	0.003	1	1
rs6581928	12	68898502	0.038	0.027	0.045	1	
rs1371578	12	72403177	0.021	0.001	0.033		
rs7965034	12	72642095	0.029	0.047	0.002		
rs11180523	12	74128034	0.021	0.044	0.008		
rs7315847	12	74596228	0.029	0.040	0.032		
rs7311560	12	75107386	0.063	0.003	0.000	1	1
rs4469939	12	75790814	0.035	0.056	0.040		
rs10777960	12	77321333	0.039	0.093	0.024	1	1
rs2279664	12	79694310	0.017	0.042	0.005		
rs10862802	12	82845715	0.025	0.052	0.008		
rs977781	12	85659181	0.057	0.002	0.020	1	
rs1344791	12	85966742	0.024	0.017	0.032		
rs4429143	12	86911662	0.035	0.000	0.047	1	
rs3782176	12	87441601	0.058	0.028	0.011	1	
rs10161354	12	87847957	0.024	0.046	0.037		
rs11611632	12	87948007	0.047	0.043	0.053	1	1
rs2116497	12	89289105	0.019	0.033	0.032		
rs1864785	12	89392018	0.029	0.053	0.004		
rs12426486	12	91030565	0.019	0.001	0.038		
rs4760506	12	91835518	0.037	0.057	0.000		
rs11107520	12	93269878	0.031	0.053	0.012		
rs11109097	12	96430905	0.017	0.003	0.035		
rs10492273	12	97736221	0.047	0.091	0.014	1	1
rs17472445	12	98487382	0.027	0.053	0.001		
rs9651924	12	99991966	0.057	0.029	0.005	1	
rs2062167	12	100116871	0.041	0.065	0.004	1	
rs35760	12	101452685	0.029	0.053	0.031		
rs7959287	12	102104105	0.040	0.080	0.043	1	
rs7306825	12	102273883	0.065	0.005	0.003	1	1
rs954921	12	102676097	0.018	0.002	0.036		
rs4964392	12	104512909	0.023	0.056	0.026		
rs2912750	12	105045414	0.041	0.095	0.008	1	1

rs1344608	12	107031552	0.035	0.057	0.000		
rs4964246	12	107205027	0.053	0.000	0.000		
rs4766542	12	110053212	0.048	0.053	0.015		
rs1920582	12	113047957	0.036	0.061	0.001	1	
rs4073170	12	113552756	0.055	0.006	0.042		
rs10507259	12	114284031	0.036	0.063	0.003	1	
rs2254578	12	115292641	0.022	0.049	0.003		
rs1446425	12	116579181	0.021	0.049	0.005		
rs1151901	12	117132554	0.021	0.000	0.032		
rs2649951	12	117669215	0.019	0.001	0.036		
rs7296507	12	120309855	0.030	0.065	0.008	1	
rs1798192	12	121725648	0.067	0.076	0.018	1	1
rs7973683	12	122974103	0.025	0.051	0.002		
rs1609942	12	123259607	0.066	0.015	0.027	1	1
rs837957	12	123621910	0.031	0.045	0.001		
rs838853	12	123729252	0.038	0.010	0.036		
rs325090	12	124392483	0.062	0.013	0.040	1	
rs2240496	12	124664405	0.031	0.047	0.005		
rs2191192	12	125258961	0.022	0.006	0.035		
rs2302270	12	125436923	0.018	0.043	0.013		
rs10847264	12	126042985	0.025	0.049	0.014		
rs1490889	12	126689766	0.063	0.099	0.020	1	1
rs10847463	12	126804305	0.036	0.004	0.050	1	1
rs1514897	12	127288955	0.033	0.052	0.018		
rs7135137	12	127664895	0.055	0.039	0.000		
rs10847674	12	127742893	0.031	0.020	0.045	1	
rs898802	12	129123565	0.066	0.068	0.000	1	1
rs11830243	12	131210924	0.027	0.045	0.005		
rs12423631	12	131273116	0.055	0.040	0.023		
rs10870511	12	131972193	0.025	0.001	0.034		
rs7981716	13	18649932	0.033	0.059	0.002	1	
rs1547721	13	19766060	0.058	0.104	0.001	1	
rs9506777	13	21534768	0.040	0.045	0.000		
rs1323168	13	21542217	0.020	0.000	0.037		
rs9316766	13	21590292	0.054	0.044	0.020		
rs9580490	13	22447148	0.065	0.081	0.000	1	1
rs7323053	13	22710567	0.014	0.009	0.034		
rs2147990	13	23397249	0.024	0.007	0.034		
rs12427946	13	23476484	0.055	0.115	0.007		
rs4770718	13	24083487	0.031	0.064	0.014	1	
rs2722	13	24183768	0.021	0.006	0.041		
rs7995087	13	24894178	0.020	0.026	0.045	1	
rs4442642	13	25090118	0.024	0.048	0.024		
rs2094497	13	26808122	0.039	0.080	0.050	1	1
rs7987436	13	26828545	0.044	0.086	0.044	1	1
rs9508029	13	27889988	0.036	0.080	0.003	1	1
rs1495934	13	28361968	0.025	0.000	0.036		
rs4594093	13	28905523	0.046	0.070	0.000	1	1
rs9551722	13	29188947	0.055	0.035	0.018		
rs278040	13	29357712	0.019	0.032	0.040		
rs2150796	13	30047837	0.021	0.050	0.020		

rs6561013	13	31412050	0.028	0.062	0.027	1	
rs206319	13	31884219	0.017	0.017	0.032		
rs10492405	13	32659455	0.029	0.049	0.008		
rs4500590	13	34979108	0.027	0.061	0.009	1	
rs4941815	13	35207635	0.056	0.015	0.006	1	
rs1517881	13	36027895	0.053	0.072	0.002	1	1
rs1517887	13	36102783	0.058	0.002	0.001	1	
rs1535520	13	36202614	0.029	0.000	0.037		
rs1322806	13	36847152	0.024	0.056	0.023		
rs9547947	13	37029993	0.031	0.002	0.037		
rs658206	13	37774887	0.023	0.003	0.047	1	
rs656044	13	37775361	0.020	0.045	0.012		
rs9634767	13	41168305	0.043	0.088	0.017	1	1
rs11620516	13	41483714	0.069	0.065	0.024	1	1
rs9315923	13	41968894	0.027	0.060	0.026	1	
rs7984001	13	42384473	0.026	0.004	0.044	1	
rs1008913	13	42627828	0.039	0.049	0.010		
rs2050638	13	44239834	0.055	0.051	0.019		
rs1984191	13	44269573	0.027	0.000	0.040		
rs1175397	13	44390146	0.027	0.061	0.031	1	
rs7998252	13	45018410	0.030	0.045	0.000		
rs1172416	13	47119744	0.024	0.045	0.013		
rs9535388	13	49339862	0.028	0.063	0.016	1	
rs484304	13	50076371	0.042	0.095	0.008	1	1
rs1328361	13	50492742	0.057	0.000	0.073	1	
rs913465	13	50493338	0.056	0.000	0.077	1	1
rs2488376	13	52972416	0.028	0.009	0.055	1	1
rs9536723	13	53908334	0.038	0.071	0.005	1	1
rs3105070	13	54888586	0.047	0.086	0.004	1	1
rs166861	13	55557523	0.029	0.051	0.012		
rs577241	13	56596687	0.027	0.045	0.003		
rs559648	13	56601847	0.022	0.032	0.037		
rs9538282	13	58519787	0.037	0.089	0.010	1	1
rs777771	13	59710657	0.020	0.051	0.007		
rs3106594	13	60557845	0.033	0.063	0.000	1	
rs3010965	13	60937883	0.033	0.006	0.045	1	
rs12430149	13	64106367	0.018	0.013	0.033		
rs1629705	13	64696304	0.034	0.000	0.035		
rs9592458	13	65719917	0.027	0.053	0.034		
rs1015870	13	66696251	0.040	0.061	0.000	1	
rs7992379	13	67023621	0.032	0.013	0.044	1	
rs17086731	13	69782413	0.027	0.020	0.059	1	1
rs7987027	13	70111148	0.045	0.077	0.003	1	1
rs966168	13	71299046	0.097	0.026	0.001	1	1
rs9572987	13	71936940	0.032	0.075	0.029	1	1
rs9542959	13	71937310	0.027	0.065	0.034		
rs1333099	13	72589237	0.054	0.006	0.095	1	1
rs7327131	13	72699429	0.060	0.005	0.073	1	
rs7983696	13	72842112	0.028	0.058	0.003	1	
rs7321013	13	73776938	0.060	0.005	0.023	1	
rs525884	13	74689394	0.034	0.082	0.029	1	1

rs7335576	13	74941319	0.062	0.044	0.021	1	
rs9593147	13	75440088	0.045	0.081	0.023	1	1
rs384857	13	76032801	0.022	0.007	0.039		
rs642394	13	76463656	0.040	0.086	0.004	1	1
rs1144384	13	77261064	0.094	0.029	0.015	1	1
rs1324791	13	77642784	0.020	0.043	0.014		
rs1417673	13	78402812	0.023	0.044	0.034		
rs6563138	13	79309824	0.021	0.046	0.011		
rs2876770	13	81083358	0.018	0.009	0.040		
rs261475	13	81237360	0.054	0.015	0.006		
rs1333592	13	82096952	0.028	0.014	0.046	1	
rs1386706	13	82314548	0.024	0.051	0.024		
rs1331944	13	83239028	0.024	0.047	0.005		
rs1413854	13	83394769	0.043	0.034	0.040		
rs4564467	13	83827832	0.063	0.056	0.008	1	1
rs4773945	13	86733845	0.019	0.017	0.044	1	
rs1325330	13	89846938	0.044	0.094	0.015	1	1
rs7997883	13	92310485	0.023	0.042	0.000		
rs9561401	13	93026747	0.020	0.017	0.048	1	
rs7990128	13	93084509	0.031	0.076	0.018	1	1
rs1886932	13	93749266	0.028	0.060	0.016	1	
rs7330519	13	94673419	0.025	0.053	0.001		
rs7330673	13	94740493	0.020	0.004	0.037		
rs4772035	13	97358819	0.039	0.089	0.001	1	1
rs9517237	13	97711127	0.024	0.008	0.037		
rs6491500	13	98871666	0.034	0.072	0.003	1	1
rs927596	13	99334817	0.062	0.034	0.037	1	
rs7324365	13	99399328	0.049	0.001	0.062	1	1
rs1999168	13	101654507	0.037	0.070	0.025	1	1
rs1434345	13	103996510	0.025	0.051	0.009		
rs3916959	13	104640379	0.054	0.002	0.006		
rs3916972	13	105044635	0.025	0.003	0.049	1	
rs2149145	13	105192803	0.026	0.062	0.023	1	
rs1550602	13	105558962	0.018	0.000	0.034		
rs9520124	13	106061919	0.024	0.053	0.018		
rs4480661	13	106095440	0.018	0.002	0.033		
rs9558993	13	106734506	0.034	0.034	0.042	1	
rs3858807	13	107267996	0.036	0.056	0.001		
rs1235133	13	107550437	0.021	0.004	0.038		
rs17485138	13	108300850	0.026	0.032	0.053	1	1
rs9555547	13	108499628	0.022	0.045	0.003		
rs9521415	13	108968251	0.054	0.002	0.017		
rs630943	13	109675047	0.035	0.086	0.013	1	1
rs328794	13	110246771	0.026	0.001	0.035		
rs4773281	13	110382375	0.037	0.051	0.008		
rs1151449	13	110931714	0.027	0.038	0.032		
rs7986446	13	112283063	0.032	0.050	0.000		
rs9604118	13	113154699	0.054	0.105	0.005		
rs3813131	13	114108121	0.027	0.056	0.001		
rs4981264	14	20130405	0.052	0.123	0.024	1	1
rs17182151	14	20348310	0.054	0.000	0.000		

rs4981361	14	20690535	0.038	0.001	0.051	1	1
rs7151770	14	21149744	0.022	0.046	0.003		
rs11621456	14	21681537	0.028	0.058	0.013	1	
rs1005495	14	21710981	0.016	0.004	0.034		
rs1952426	14	22184362	0.069	0.012	0.000	1	1
rs10132585	14	22399286	0.022	0.047	0.009		
rs2239578	14	22973220	0.029	0.014	0.051	1	1
rs3212240	14	23880738	0.022	0.010	0.037		
rs5250	14	24044461	0.034	0.043	0.009		
rs2146918	14	24470949	0.044	0.007	0.045	1	
rs11846747	14	25833243	0.018	0.006	0.033		
rs10143739	14	27156570	0.029	0.044	0.035		
rs1954578	14	27457754	0.036	0.066	0.008	1	
rs17114073	14	28135440	0.032	0.003	0.059	1	1
rs17630716	14	28300611	0.085	0.096	0.000	1	1
rs7159513	14	28926702	0.066	0.097	0.000	1	1
rs10498313	14	29468627	0.020	0.049	0.004		
rs1278886	14	31460960	0.035	0.043	0.002		
rs1958913	14	31582647	0.030	0.006	0.033		
rs727447	14	33005973	0.030	0.017	0.053	1	1
rs2000015	14	33550151	0.036	0.011	0.074	1	1
rs1244376	14	33748414	0.051	0.062	0.027	1	
rs6571727	14	35210859	0.066	0.082	0.000	1	1
rs8009480	14	35549250	0.034	0.001	0.040		
rs378836	14	35561627	0.086	0.020	0.003	1	1
rs718997	14	36512337	0.059	0.068	0.000	1	
rs718998	14	36512422	0.056	0.074	0.000	1	1
rs3809444	14	37437935	0.023	0.042	0.004		
rs2415449	14	37667678	0.054	0.002	0.000		
rs1955540	14	39871501	0.020	0.008	0.034		
rs1431031	14	40854451	0.032	0.045	0.036		
rs10498389	14	43028963	0.016	0.015	0.032		
rs1955985	14	43649706	0.025	0.004	0.037		
rs3007103	14	46436090	0.018	0.007	0.039		
rs10483565	14	47086453	0.023	0.053	0.003		
rs4255712	14	48854983	0.021	0.004	0.037		
rs7140473	14	49746697	0.069	0.001	0.000	1	1
rs1890978	14	49914606	0.022	0.002	0.045	1	
rs7143869	14	50397972	0.023	0.041	0.001		
rs10498432	14	51269221	0.034	0.057	0.001		
rs809116	14	51730248	0.057	0.022	0.002	1	
rs7494379	14	52481141	0.068	0.054	0.011	1	1
rs1958909	14	52514477	0.065	0.082	0.001	1	1
rs4901444	14	53295858	0.032	0.044	0.001		
rs883598	14	55285230	0.025	0.001	0.047	1	
rs1957889	14	55644910	0.028	0.047	0.005		
rs17091959	14	56227519	0.027	0.001	0.045	1	
rs2881345	14	56737476	0.042	0.053	0.000		
rs1080241	14	57250437	0.039	0.063	0.001	1	
rs11158187	14	57507106	0.022	0.002	0.045	1	
rs698020	14	58193533	0.029	0.045	0.002		

rs4151374	14	60471706	0.033	0.065	0.001	1	
rs217692	14	61473002	0.024	0.000	0.037		
rs2353777	14	61616009	0.032	0.050	0.016		
rs2883559	14	62008482	0.055	0.009	0.034		
rs12588552	14	62406682	0.071	0.017	0.005	1	1
rs10143368	14	64761847	0.022	0.052	0.016		
rs1256501	14	64793860	0.027	0.016	0.051	1	1
rs7160924	14	65388154	0.021	0.008	0.045	1	
rs1956529	14	67858677	0.038	0.063	0.001	1	
rs8007929	14	69270216	0.032	0.060	0.001	1	
rs1952202	14	69431827	0.026	0.020	0.038		
rs10483832	14	70130590	0.025	0.020	0.053	1	1
rs3814872	14	70297666	0.019	0.045	0.014		
rs10483853	14	72826052	0.025	0.045	0.039		
rs10143790	14	75391574	0.043	0.072	0.008	1	1
rs10483879	14	76367923	0.030	0.070	0.010	1	1
rs1013652	14	76583644	0.030	0.000	0.044	1	
rs158105	14	76699434	0.060	0.023	0.025	1	
rs2052444	14	77382671	0.031	0.027	0.035		
rs1569326	14	77721949	0.029	0.055	0.005		
rs6574472	14	78338055	0.021	0.005	0.044	1	
rs7141906	14	78737063	0.066	0.008	0.033	1	1
rs2205226	14	79425094	0.031	0.000	0.045	1	
rs9323682	14	79536316	0.032	0.060	0.015	1	
rs7159238	14	81135391	0.039	0.000	0.061	1	1
rs17116436	14	81726882	0.045	0.051	0.000		
rs10498554	14	82441909	0.039	0.051	0.001		
rs11852029	14	82892479	0.056	0.045	0.015	1	
rs1481420	14	84229253	0.023	0.024	0.040		
rs2625342	14	84342096	0.033	0.073	0.007	1	1
rs10484101	14	85887379	0.026	0.000	0.032		
rs8022732	14	86360515	0.064	0.013	0.021	1	1
rs1957443	14	87065371	0.023	0.047	0.033		
rs10484021	14	88945650	0.036	0.001	0.040		
rs435376	14	89191686	0.057	0.065	0.012	1	
rs2181663	14	89193260	0.043	0.071	0.003	1	1
rs1889360	14	89691182	0.018	0.004	0.035		
rs10139335	14	90637635	0.083	0.060	0.000	1	1
rs11850195	14	92396614	0.069	0.000	0.069	1	1
rs3783913	14	92527504	0.030	0.061	0.001	1	
rs6575340	14	93093725	0.034	0.077	0.007	1	1
rs1243519	14	94370110	0.031	0.052	0.000		
rs9671369	14	94976074	0.028	0.008	0.055	1	1
rs3742480	14	95626021	0.016	0.005	0.034		
rs10149792	14	95702880	0.050	0.078	0.000	1	1
rs1885159	14	96458922	0.028	0.048	0.012		
rs1351805	14	96877218	0.036	0.057	0.035		
rs2781388	14	97369867	0.042	0.094	0.011	1	1
rs1456988	14	97557760	0.029	0.032	0.065	1	1
rs10141191	14	97957263	0.049	0.062	0.000	1	
rs1257676	14	98567665	0.033	0.053	0.000		

rs941566	14	99090171	0.026	0.045	0.050	1	
rs2145505	14	100627149	0.054	0.005	0.019		
rs11625070	14	100749749	0.029	0.062	0.014	1	
rs729438	14	103162542	0.024	0.020	0.032		
rs8019526	14	103930464	0.021	0.049	0.007		
rs4247027	14	104210939	0.085	0.032	0.005	1	1
rs1008628	14	104793771	0.021	0.044	0.000		
rs17112970	14	106107737	0.060	0.030	0.010	1	
rs988132	14	106256042	0.018	0.003	0.032		
rs2105992	14	106331778	0.043	0.062	0.000	1	
rs2289823	15	20479393	0.029	0.045	0.001		
rs11632341	15	21540364	0.026	0.000	0.042	1	
rs4778346	15	21686776	0.036	0.076	0.003	1	1
rs486535	15	23973086	0.017	0.012	0.037		
rs737098	15	24518263	0.033	0.064	0.019	1	
rs1432124	15	24851437	0.022	0.043	0.037		
rs1571280	15	25445120	0.039	0.065	0.000	1	
rs16950272	15	25552496	0.029	0.012	0.034		
rs1490079	15	27091095	0.054	0.022	0.000		
rs983901	15	27287591	0.026	0.062	0.007	1	
rs2125657	15	27693153	0.056	0.001	0.098	1	1
rs785450	15	27928636	0.022	0.049	0.005		
rs711355	15	27980760	0.060	0.006	0.019	1	
rs10519726	15	29109167	0.061	0.001	0.003	1	
rs2279662	15	31739202	0.025	0.046	0.000		
rs2293028	15	31802205	0.017	0.003	0.033		
rs644339	15	32820148	0.024	0.000	0.042		
rs2067724	15	32885447	0.041	0.069	0.007	1	1
rs2647682	15	33819520	0.055	0.016	0.002	1	
rs2256109	15	33827156	0.031	0.003	0.059	1	1
rs2947253	15	33836784	0.034	0.068	0.000	1	
rs1349344	15	34987349	0.023	0.020	0.045	1	
rs954431	15	36754057	0.028	0.007	0.046	1	
rs8024474	15	36799871	0.061	0.005	0.039	1	
rs973214	15	36904478	0.042	0.079	0.004	1	1
rs9302101	15	37382976	0.023	0.024	0.049	1	
rs1478606	15	37598478	0.024	0.043	0.004		
rs11630209	15	39680412	0.022	0.043	0.004		
rs1712429	15	40199305	0.022	0.014	0.045	1	
rs2255235	15	42790656	0.056	0.052	0.006	1	
rs1656623	15	45602776	0.038	0.062	0.014	1	
rs4775777	15	46918903	0.022	0.047	0.006		
rs4775845	15	47965714	0.059	0.060	0.000	1	
rs10519293	15	49259313	0.024	0.035	0.055	1	1
rs11070857	15	49644512	0.042	0.045	0.008		
rs12595160	15	50250777	0.019	0.007	0.039		
rs1693523	15	50614631	0.075	0.021	0.000	1	1
rs1501029	15	51100411	0.023	0.054	0.005		
rs12594449	15	51253705	0.042	0.016	0.036		
rs10518760	15	52312958	0.056	0.006	0.000	1	
rs12902903	15	52327884	0.030	0.000	0.032		

rs12050587	15	53414820	0.029	0.013	0.050	1	
rs11631355	15	53868918	0.023	0.052	0.007		
rs1511954	15	55486254	0.044	0.001	0.073	1	1
rs11856153	15	55836591	0.036	0.083	0.005	1	1
rs8032239	15	56292414	0.059	0.000	0.006	1	
rs10518978	15	56543465	0.024	0.053	0.009		
rs6083	15	56625302	0.039	0.004	0.057	1	1
rs1479884	15	57669034	0.034	0.048	0.000		
rs8033985	15	58409103	0.035	0.058	0.004	1	
rs4774394	15	59342906	0.027	0.014	0.066	1	1
rs1048570	15	60907059	0.028	0.037	0.049	1	
rs1463803	15	60915699	0.025	0.050	0.028		
rs352476	15	63592442	0.022	0.003	0.041		
rs1355857	15	64340703	0.060	0.078	0.000	1	
rs2278545	15	65253933	0.028	0.049	0.002		
rs11071986	15	66279082	0.032	0.062	0.002	1	
rs1392635	15	67185080	0.025	0.047	0.010		
rs2899755	15	67842954	0.055	0.039	0.002		
rs304955	15	67960053	0.029	0.063	0.023	1	
rs13379488	15	68210624	0.027	0.002	0.051	1	1
rs1713551	15	69096647	0.020	0.002	0.044	1	
rs4777409	15	69623478	0.029	0.000	0.039		
rs3825804	15	69857059	0.032	0.061	0.017	1	
rs2469534	15	74396778	0.025	0.045	0.006		
rs11072756	15	76441866	0.054	0.038	0.012		
rs1032975	15	77340889	0.065	0.083	0.000	1	1
rs11259964	15	81536538	0.027	0.005	0.044	1	
rs882888	15	82551193	0.021	0.049	0.014		
rs2937947	15	84162420	0.032	0.058	0.002	1	
rs2346713	15	84879341	0.021	0.044	0.027		
rs4609816	15	84903629	0.033	0.040	0.034		
rs10520648	15	85419597	0.040	0.016	0.036		
rs1034482	15	85428116	0.042	0.064	0.019	1	
rs4887326	15	86142404	0.034	0.044	0.005		
rs7164531	15	86615360	0.027	0.002	0.040		
rs4932432	15	87173605	0.035	0.084	0.019	1	1
rs7171194	15	88585254	0.028	0.060	0.007	1	
rs4290453	15	90127824	0.029	0.057	0.011		
rs12912184	15	90659105	0.031	0.015	0.073	1	1
rs1386775	15	90668194	0.026	0.044	0.034		
rs12593919	15	91450126	0.034	0.056	0.007		
rs294558	15	92656980	0.055	0.007	0.024	1	
rs4491457	15	93187447	0.109	0.099	0.001	1	1
rs955830	15	96164512	0.045	0.061	0.000	1	
rs4426332	15	97040118	0.060	0.024	0.019	1	
rs2684777	15	97214217	0.025	0.005	0.032		
rs1546713	15	97289389	0.056	0.056	0.018		
rs325383	15	98072569	0.043	0.047	0.000		
rs749516	15	98112073	0.058	0.045	0.005	1	
rs9920347	15	98156854	0.025	0.004	0.040		
rs4533267	15	98603794	0.048	0.077	0.000	1	1

rs2955965	15	99727305	0.058	0.116	0.008	1		
rs1436115	16	5853774	0.025	0.045	0.000			
rs1463243	16	6121345	0.047	0.009	0.041			
rs10492841	16	6679934	0.058	0.021	0.001	1		
rs12933074	16	6816985	0.046	0.087	0.012	1	1	
rs4786169	16	7436376	0.025	0.031	0.034			
rs7184130	16	7564046	0.028	0.046	0.005			
rs2313427	16	8722501	0.029	0.000	0.052	1	1	
rs1542541	16	8933901	0.067	0.051	0.022	1	1	
rs4628972	16	9934617	0.036	0.086	0.021	1	1	
rs7201574	16	10010559	0.024	0.017	0.052	1	1	
rs154148	16	11863979	0.036	0.068	0.005	1		
rs1346491	16	12708927	0.022	0.000	0.037			
rs204029	16	13227655	0.025	0.049	0.002			
rs3928240	16	14362021	0.026	0.050	0.003			
rs4321183	16	16823798	0.026	0.001	0.045	1		
rs11647626	16	17066939	0.070	0.023	0.004	1	1	
rs7186245	16	17545108	0.049	0.074	0.000	1	1	
rs8063494	16	17558047	0.025	0.008	0.052	1	1	
rs10492784	16	19274084	0.041	0.052	0.014			
rs2545886	16	20936698	0.026	0.005	0.040			
rs11074489	16	21118779	0.031	0.048	0.001			
rs10852259	16	23975815	0.039	0.052	0.000			
rs8054383	16	24364338	0.022	0.006	0.044	1		
rs6497910	16	25917161	0.034	0.057	0.003			
rs182679	16	26659551	0.027	0.000	0.032			
rs10400960	16	26738119	0.033	0.047	0.009			
rs8056488	16	27210230	0.027	0.000	0.037			
rs1072594	16	27758121	0.031	0.049	0.001			
rs8060853	16	30311359	0.027	0.042	0.010			
rs7200879	16	30855073	0.055	0.057	0.023			
rs4889545	16	31428777	0.031	0.052	0.039			
rs7206909	16	46794167	0.055	0.052	0.000			
rs8054696	16	47050362	0.040	0.097	0.024	1	1	
rs11649236	16	47189979	0.017	0.015	0.041			
rs1345405	16	47681629	0.069	0.096	0.004	1	1	
rs1510986	16	47722142	0.122	0.095	0.013	1	1	
rs8056754	16	47789853	0.072	0.017	0.045	1		
rs4785365	16	48506133	0.066	0.120	0.002	1	1	
rs12913	16	49225927	0.073	0.028	0.000	1	1	
rs2193098	16	49767996	0.033	0.060	0.006	1		
rs2648005	16	50124541	0.061	0.004	0.049	1		
rs1111316	16	50161400	0.034	0.027	0.065	1	1	
rs12595945	16	50291509	0.032	0.076	0.013	1	1	
rs9302555	16	51086348	0.031	0.051	0.000			
rs2110830	16	51408972	0.033	0.003	0.035			
rs8061239	16	52469967	0.030	0.056	0.059	1	1	
rs10521320	16	54133662	0.046	0.092	0.000	1	1	
rs17281761	16	54902878	0.038	0.065	0.005	1		
rs2303785	16	56495289	0.024	0.035	0.033			
rs7194392	16	57560512	0.024	0.053	0.009			

rs7201354	16	59390566	0.038	0.080	0.015	1	1
rs986348	16	59575312	0.020	0.013	0.042	1	
rs1162202	16	60524204	0.056	0.090	0.003	1	
rs2902323	16	67293793	0.068	0.042	0.003	1	1
rs4788692	16	71623157	0.061	0.045	0.025	1	
rs1517176	16	72142031	0.038	0.007	0.039		
rs929878	16	72848142	0.023	0.000	0.039		
rs8049101	16	73463615	0.017	0.017	0.035		
rs2194295	16	74798976	0.054	0.024	0.006		
rs8062038	16	74850593	0.044	0.070	0.051	1	1
rs308927	16	76326341	0.028	0.023	0.058	1	1
rs6564507	16	76660674	0.038	0.045	0.000		
rs11645023	16	78483635	0.035	0.073	0.009	1	1
rs7189799	16	78894664	0.072	0.003	0.009	1	1
rs881083	16	79210284	0.047	0.015	0.043	1	
rs9929106	16	80177464	0.087	0.130	0.002	1	1
rs4782622	16	81027428	0.019	0.008	0.040		
rs244844	16	82862175	0.039	0.014	0.061	1	1
rs173434	16	82869458	0.054	0.010	0.024		
rs9935170	16	83570758	0.020	0.041	0.009		
rs11643717	16	83870384	0.025	0.000	0.032		
rs10521318	16	84568838	0.025	0.045	0.001		
rs4843516	16	85667972	0.046	0.050	0.000		
rs12444562	16	86214995	0.019	0.043	0.009		
rs2011877	16	88342319	0.062	0.071	0.000	1	
rs7223902	17	779934	0.030	0.000	0.032		
rs9895971	17	1403257	0.036	0.054	0.004		
rs7503422	17	1662185	0.018	0.013	0.035		
rs5030740	17	1747350	0.055	0.044	0.004		
rs903160	17	2038515	0.048	0.060	0.002	1	
rs1476462	17	2654355	0.053	0.058	0.004	1	
rs2062184	17	3116208	0.063	0.032	0.005	1	1
rs422159	17	3395472	0.051	0.065	0.001	1	
rs2176324	17	3890292	0.059	0.004	0.017	1	
rs2309487	17	5777196	0.031	0.053	0.000		
rs3817003	17	8304349	0.020	0.001	0.040		
rs9901968	17	9011371	0.020	0.049	0.010		
rs8070490	17	9833581	0.016	0.021	0.036		
rs9913953	17	11156730	0.050	0.097	0.056	1	1
rs8070243	17	11692776	0.025	0.009	0.041		
rs10521210	17	12966928	0.057	0.025	0.001	1	
rs3967117	17	13277382	0.037	0.054	0.002		
rs8066913	17	14067353	0.059	0.008	0.028	1	
rs2127994	17	14239319	0.057	0.010	0.040		
rs671098	17	14338935	0.038	0.060	0.000	1	
rs2058178	17	14902367	0.026	0.000	0.040		
rs4792606	17	15265393	0.052	0.128	0.019	1	1
rs3813766	17	16498331	0.078	0.097	0.036	1	1
rs7226068	17	17172156	0.025	0.000	0.038		
rs4244595	17	18618054	0.055	0.056	0.004		
rs241771	17	23617073	0.034	0.048	0.022		

rs11080058	17	23766187	0.059	0.017	0.023	1	
rs883828	17	24777972	0.028	0.043	0.000		
rs1989805	17	27897975	0.028	0.048	0.000		
rs1021891	17	28735085	0.039	0.043	0.000		
rs797993	17	30432233	0.020	0.043	0.018		
rs295869	17	32221458	0.036	0.044	0.000		
rs9899502	17	33987599	0.033	0.007	0.037		
rs12453325	17	34190711	0.054	0.013	0.010		
rs16235	17	34526657	0.025	0.054	0.004		
rs2023906	17	35967212	0.033	0.004	0.035		
rs9303297	17	36259516	0.064	0.046	0.001		
rs9303298	17	36264327	0.066	0.046	0.001	1	1
rs2074107	17	39693502	0.024	0.021	0.044	1	
rs9908594	17	40328839	0.017	0.016	0.034		
rs4525555	17	42692948	0.054	0.009	0.006		
rs9900218	17	47055553	0.013	0.006	0.033		
rs12938412	17	47076013	0.020	0.044	0.005		
rs7207559	17	50886802	0.044	0.088	0.007	1	1
rs7225691	17	50920224	0.092	0.077	0.038	1	1
rs1553677	17	50928901	0.039	0.002	0.043	1	
rs9303389	17	52424031	0.035	0.035	0.062	1	1
rs7213565	17	53073036	0.030	0.043	0.009		
rs2070720	17	59372505	0.021	0.042	0.032		
rs2869825	17	60714723	0.028	0.049	0.018		
rs3760292	17	61656602	0.018	0.004	0.032		
rs1808693	17	62917942	0.038	0.045	0.025		
rs11658071	17	63046267	0.024	0.034	0.050	1	1
rs740519	17	64659545	0.033	0.053	0.001		
rs9895649	17	64769828	0.029	0.000	0.045	1	
rs2367008	17	66294236	0.041	0.048	0.023		
rs7214479	17	66702544	0.025	0.029	0.042	1	
rs2430549	17	67244986	0.025	0.000	0.039		
rs6501538	17	67885084	0.054	0.006	0.005		
rs7502006	17	68414311	0.024	0.009	0.037		
rs8065206	17	68859227	0.043	0.077	0.008	1	1
rs7220636	17	69021112	0.065	0.047	0.001	1	1
rs1522875	17	70084003	0.065	0.109	0.054	1	1
rs4789396	17	72503206	0.023	0.005	0.037		
rs971626	17	74928535	0.042	0.088	0.001	1	1
rs870681	17	75388471	0.067	0.000	0.012	1	1
rs3185057	17	75978442	0.020	0.017	0.037		
rs4969429	17	76379814	0.032	0.048	0.001		
rs899725	18	481967	0.022	0.013	0.034		
rs7231736	18	831321	0.027	0.060	0.009	1	
rs10513945	18	1466737	0.033	0.023	0.048	1	
rs10513935	18	1650497	0.060	0.092	0.004	1	
rs8095716	18	2511210	0.029	0.007	0.039		
rs506739	18	2846536	0.041	0.048	0.000		
rs4239320	18	3041297	0.027	0.011	0.054	1	1
rs9949946	18	3787659	0.058	0.130	0.006	1	
rs1701507	18	4474175	0.020	0.026	0.035		

rs8086761	18	4802752	0.066	0.116	0.006	1	1
rs1785394	18	5550258	0.024	0.045	0.009		
rs1917925	18	5749683	0.025	0.022	0.035		
rs334411	18	7111227	0.047	0.071	0.014	1	1
rs576192	18	7509001	0.057	0.008	0.002	1	
rs567595	18	8056414	0.030	0.002	0.032		
rs8092501	18	8981193	0.021	0.043	0.005		
rs206492	18	10277586	0.033	0.000	0.042	1	
rs1343886	18	10751475	0.024	0.052	0.012		
rs6505593	18	10781748	0.059	0.040	0.000	1	
rs4629032	18	11158361	0.022	0.005	0.038		
rs550315	18	13123577	0.032	0.066	0.007	1	
rs872906	18	13367699	0.019	0.018	0.043	1	
rs8099745	18	17971699	0.030	0.000	0.035		
rs894930	18	19948158	0.070	0.039	0.001	1	1
rs339935	18	20535478	0.029	0.053	0.000		
rs4516308	18	21248308	0.026	0.045	0.005		
rs7242518	18	22368804	0.020	0.025	0.037		
rs3763043	18	22689816	0.028	0.068	0.023	1	
rs1105237	18	23248050	0.034	0.071	0.009	1	1
rs8094680	18	23489448	0.017	0.013	0.032		
rs4445973	18	25397159	0.033	0.000	0.045	1	
rs1943575	18	26556026	0.023	0.000	0.039		
rs1466379	18	27310463	0.029	0.068	0.010	1	
rs10502584	18	28051476	0.031	0.051	0.001		
rs7239282	18	28855794	0.025	0.017	0.032		
rs4799355	18	29567122	0.046	0.071	0.000	1	1
rs2422313	18	31350797	0.029	0.065	0.004	1	
rs3786276	18	32091493	0.054	0.061	0.003		
rs11660741	18	33238069	0.055	0.078	0.001	1	1
rs2226726	18	33241353	0.058	0.067	0.002	1	
rs10853457	18	34419615	0.021	0.048	0.011		
rs2953409	18	34833086	0.055	0.001	0.004	1	
rs8089915	18	35605604	0.031	0.000	0.037		
rs431856	18	38175145	0.017	0.008	0.032		
rs16977580	18	39401490	0.030	0.052	0.001		
rs10853522	18	40494596	0.025	0.055	0.010		
rs1026186	18	40995408	0.024	0.032	0.049	1	
rs7237823	18	41601433	0.028	0.057	0.033		
rs9304333	18	42450420	0.048	0.052	0.000		
rs10502874	18	42480771	0.020	0.016	0.039		
rs4940307	18	43954301	0.063	0.030	0.009	1	1
rs4940348	18	44029179	0.039	0.060	0.000	1	
rs7229278	18	45276924	0.033	0.005	0.043	1	
rs2852100	18	45845366	0.044	0.054	0.066	1	1
rs1822462	18	46590325	0.066	0.123	0.030	1	1
rs322520	18	47435180	0.047	0.005	0.065	1	1
rs1480925	18	47744588	0.036	0.047	0.009		
rs1221869	18	48406960	0.035	0.061	0.001	1	
rs11082932	18	48434137	0.022	0.016	0.040		
rs8087644	18	49683496	0.054	0.006	0.019		

rs8086877	18	49928133	0.044	0.004	0.062	1	1
rs4800967	18	50626639	0.031	0.003	0.040		
rs2276195	18	51047507	0.032	0.042	0.004		
rs10871622	18	51677998	0.029	0.008	0.037		
rs1624292	18	53923233	0.037	0.057	0.008		
rs8083963	18	54142011	0.023	0.034	0.042	1	
rs6420554	18	54478472	0.027	0.045	0.027		
rs1400531	18	55017017	0.022	0.045	0.001		
rs6567072	18	55072290	0.072	0.002	0.018	1	1
rs1791320	18	55515946	0.036	0.005	0.044	1	
rs11152223	18	56257038	0.021	0.017	0.032		
rs12604169	18	57734426	0.070	0.037	0.007	1	1
rs8093819	18	57849856	0.043	0.063	0.001	1	
rs569162	18	58812521	0.013	0.005	0.032		
rs1587989	18	60554118	0.022	0.045	0.008		
rs8097554	18	61189187	0.038	0.003	0.053	1	1
rs976882	18	61305944	0.055	0.000	0.003		
rs1014824	18	62818784	0.029	0.047	0.009		
rs7506608	18	63451769	0.026	0.056	0.002		
rs964389	18	64075007	0.046	0.034	0.034		
rs7236376	18	64175633	0.072	0.152	0.013	1	1
rs1472870	18	65036963	0.031	0.065	0.003	1	
rs4325666	18	65460176	0.032	0.000	0.042	1	
rs1529220	18	66575175	0.028	0.048	0.002		
rs10513997	18	66972377	0.060	0.029	0.042	1	
rs8091618	18	67004778	0.036	0.000	0.061	1	1
rs1942371	18	67425288	0.024	0.052	0.009		
rs9957633	18	67876215	0.018	0.016	0.039		
rs6566644	18	68461160	0.059	0.026	0.008	1	
rs1539943	18	69254778	0.024	0.046	0.034		
rs8099090	18	69257965	0.035	0.085	0.016	1	1
rs4563123	18	69426411	0.056	0.072	0.003	1	
rs2702618	18	69833402	0.017	0.005	0.034		
rs1788645	18	69989839	0.029	0.061	0.000	1	
rs1558946	18	70176783	0.064	0.059	0.002	1	1
rs2849808	18	71455464	0.022	0.008	0.041		
rs2245011	18	71900446	0.038	0.060	0.000	1	
rs4890890	18	72936688	0.019	0.013	0.043	1	
rs11659609	18	74564026	0.073	0.028	0.018	1	1
rs4807291	19	2475685	0.041	0.074	0.000	1	1
rs2238640	19	5212506	0.029	0.005	0.032		
rs7256147	19	6872868	0.056	0.097	0.002	1	1
rs3895916	19	6878996	0.066	0.097	0.001	1	1
rs484870	19	7475282	0.019	0.014	0.045	1	
rs9426	19	8273158	0.023	0.009	0.049	1	
rs2015157	19	9207762	0.028	0.000	0.037		
rs1979262	19	10059441	0.035	0.052	0.027		
rs7248036	19	10509933	0.017	0.003	0.040		
rs1833619	19	13508830	0.027	0.056	0.002		
rs4528684	19	14212574	0.044	0.001	0.044	1	
rs11085905	19	14657841	0.055	0.034	0.019	1	

rs4808387	19	14700160	0.044	0.080	0.000	1	1
rs5021087	19	15008113	0.019	0.011	0.032		
rs3746199	19	17573711	0.037	0.060	0.000	1	
rs2305743	19	18054191	0.032	0.001	0.055	1	1
rs2269873	19	19253401	0.020	0.042	0.015		
rs12610185	19	19582722	0.019	0.001	0.036		
rs965945	19	35786303	0.017	0.011	0.033		
rs1982933	19	36549388	0.021	0.004	0.039		
rs8101880	19	37190066	0.030	0.053	0.013		
rs2903754	19	37783522	0.022	0.006	0.037		
rs12150889	19	38046895	0.039	0.093	0.017	1	1
rs934419	19	38152949	0.090	0.041	0.003	1	1
rs8112457	19	39827760	0.053	0.005	0.019		
rs1966961	19	43952310	0.026	0.054	0.008		
rs234346	19	45159516	0.028	0.043	0.001		
rs2304232	19	46454365	0.027	0.011	0.047	1	
rs8110384	19	51173175	0.026	0.054	0.029		
rs759290	19	51583951	0.032	0.001	0.054	1	1
rs10401590	19	52005171	0.042	0.042	0.004		
rs10426361	19	53210229	0.030	0.048	0.003		
rs1673028	19	55644865	0.063	0.018	0.027	1	1
rs2569433	19	56303075	0.051	0.047	0.005		
rs10422751	19	56930644	0.033	0.001	0.036		
rs8107315	19	57339705	0.037	0.073	0.001	1	1
rs217541	19	59135678	0.020	0.034	0.037		
rs410852	19	59492183	0.129	0.139	0.011	1	1
rs11672983	19	60074863	0.026	0.046	0.006		
rs890869	19	60324855	0.022	0.001	0.033		
rs306447	19	61241344	0.024	0.044	0.047	1	
rs7247474	19	62658274	0.036	0.057	0.031		
rs10414608	19	62773680	0.059	0.042	0.012	1	
rs1047128	19	63049358	0.020	0.009	0.032		
rs1465789	19	63637868	0.022	0.000	0.036		
rs1469781	20	118642	0.022	0.045	0.010		
rs6140113	20	691770	0.037	0.061	0.000	1	
rs4813044	20	1096783	0.019	0.002	0.034		
rs200885	20	1715896	0.077	0.082	0.011	1	1
rs6132153	20	1906145	0.038	0.001	0.055	1	1
rs6084229	20	2926928	0.058	0.094	0.015	1	
rs6076657	20	4249757	0.023	0.000	0.036		
rs402899	20	4368450	0.029	0.045	0.014		
rs4815763	20	4994930	0.024	0.008	0.036		
rs910702	20	5415063	0.041	0.053	0.002		
rs2232081	20	6008395	0.038	0.081	0.037	1	1
rs2326748	20	6241824	0.028	0.019	0.041		
rs1569727	20	7901248	0.043	0.057	0.004		
rs2745755	20	8224969	0.030	0.003	0.036		
rs926505	20	8738920	0.016	0.024	0.035		
rs226191	20	8903810	0.027	0.061	0.013	1	
rs6086900	20	9411693	0.059	0.079	0.002	1	
rs6056774	20	9572587	0.036	0.017	0.057	1	1

rs4816158	20	9580609	0.057	0.081	0.013	1	1
rs6039743	20	10088090	0.067	0.000	0.115	1	1
rs1232597	20	10553631	0.033	0.076	0.013	1	1
rs6041542	20	12609944	0.061	0.066	0.001	1	
rs6109626	20	12886069	0.048	0.096	0.000	1	1
rs719628	20	13210487	0.029	0.008	0.044	1	
rs6079149	20	13647281	0.028	0.053	0.001		
rs6079229	20	13871319	0.022	0.013	0.051	1	1
rs6042817	20	14538468	0.040	0.073	0.003	1	1
rs149820	20	15131343	0.040	0.051	0.010		
rs2021974	20	15330919	0.079	0.023	0.036		
rs6135407	20	15345676	0.098	0.051	0.003	1	1
rs6044386	20	16750429	0.022	0.001	0.039		
rs6034955	20	17992432	0.059	0.006	0.000	1	
rs6136307	20	18068761	0.030	0.042	0.036		
rs11299	20	18071022	0.032	0.078	0.017	1	1
rs6081461	20	18941730	0.021	0.012	0.034		
rs651862	20	19446915	0.032	0.051	0.000		
rs6035545	20	20009817	0.016	0.024	0.032		
rs4813431	20	21489403	0.017	0.021	0.032		
rs2757072	20	22339189	0.041	0.060	0.000	1	
rs6082952	20	22895253	0.033	0.047	0.003		
rs1102973	20	24178055	0.021	0.002	0.032		
rs6076248	20	24587916	0.023	0.051	0.001		
rs6036896	20	24661912	0.058	0.001	0.031	1	
rs7262822	20	31329358	0.047	0.099	0.000	1	1
rs6058150	20	33020478	0.032	0.045	0.026		
rs6088887	20	33634738	0.016	0.012	0.035		
rs3752292	20	37003889	0.031	0.047	0.000		
rs10485681	20	39926486	0.029	0.061	0.020	1	
rs6017227	20	42026634	0.043	0.064	0.025	1	
rs707577	20	43487763	0.030	0.058	0.045	1	
rs6074024	20	44176717	0.022	0.044	0.028		
rs7266119	20	44534168	0.019	0.000	0.035		
rs7262634	20	45267686	0.020	0.000	0.041		
rs6125196	20	46035357	0.032	0.001	0.039		
rs852371	20	46273725	0.078	0.109	0.002	1	1
rs4810898	20	46987799	0.033	0.045	0.001		
rs6013030	20	48907768	0.055	0.005	0.049		
rs2294902	20	48986311	0.037	0.066	0.052	1	1
rs4239662	20	49114684	0.035	0.084	0.033	1	1
rs6022196	20	51031721	0.036	0.001	0.046	1	
rs6063966	20	51626495	0.024	0.004	0.045	1	
rs6098293	20	52908663	0.020	0.013	0.037		
rs6069486	20	53948244	0.027	0.001	0.045	1	
rs6014727	20	54442590	0.057	0.008	0.007	1	
rs1004370	20	54617187	0.036	0.042	0.007		
rs4811766	20	54864978	0.025	0.019	0.036		
rs6128327	20	56375963	0.037	0.066	0.002	1	
rs6026243	20	56417500	0.054	0.008	0.023		
rs2064865	20	57267135	0.081	0.006	0.006	1	1

rs187989	20	57425395	0.021	0.018	0.034		
rs1946840	20	58527261	0.036	0.077	0.008	1	1
rs6100966	20	58568113	0.035	0.055	0.047	1	
rs817699	20	59192316	0.016	0.000	0.032		
rs2026164	20	59753400	0.037	0.055	0.001		
rs6142946	20	60106460	0.037	0.000	0.058	1	1
rs6062340	20	62153719	0.054	0.000	0.001		
rs996688	21	15374356	0.053	0.060	0.006		
rs2823363	21	15844564	0.032	0.077	0.044	1	
rs3737408	21	16713622	0.041	0.096	0.039		
rs964529	21	18506579	0.019	0.009	0.039		
rs2824720	21	18590848	0.027	0.054	0.019		
rs2825180	21	19124203	0.055	0.003	0.040		
rs7276040	21	19157139	0.053	0.001	0.051	1	1
rs457662	21	19248597	0.038	0.052	0.000		
rs1387618	21	19846416	0.017	0.008	0.037		
rs2256181	21	20053441	0.057	0.064	0.002	1	
rs2826498	21	21054936	0.028	0.043	0.009		
rs2849925	21	21149495	0.021	0.005	0.033		
rs2826875	21	21812513	0.041	0.068	0.001	1	
rs2828068	21	23618853	0.032	0.050	0.043	1	
rs2012307	21	24119257	0.040	0.052	0.001		
rs995938	21	24504929	0.061	0.034	0.024	1	
rs2829045	21	24701003	0.026	0.005	0.051	1	1
rs463164	21	26500739	0.030	0.070	0.002	1	1
rs2830818	21	27552800	0.070	0.077	0.006	1	1
rs2831306	21	28279043	0.055	0.031	0.011		
rs2831314	21	28282861	0.038	0.053	0.004		
rs13047178	21	28738014	0.040	0.001	0.054	1	1
rs2831914	21	28881324	0.059	0.017	0.042	1	
rs2832276	21	29530655	0.031	0.050	0.005		
rs403305	21	30039929	0.024	0.045	0.004		
rs2205177	21	30167132	0.026	0.024	0.040		
rs2832781	21	30605267	0.062	0.013	0.001	1	
rs2833401	21	31712967	0.025	0.056	0.009		
rs2834139	21	33497768	0.039	0.073	0.007	1	1
rs9976971	21	33689967	0.021	0.000	0.035		
rs2834380	21	34413370	0.029	0.056	0.020		
rs1010668	21	34660028	0.017	0.025	0.037		
rs2834651	21	35150230	0.048	0.057	0.012		
rs2834876	21	35578180	0.026	0.005	0.049	1	
rs928844	21	36921669	0.046	0.010	0.058	1	1
rs4817896	21	38123831	0.027	0.008	0.042	1	
rs2836162	21	38426226	0.034	0.058	0.000	1	
rs3746880	21	38717072	0.071	0.040	0.016	1	1
rs2150409	21	39173060	0.027	0.055	0.034		
rs2836747	21	39204426	0.049	0.014	0.070	1	1
rs2239385	21	39949617	0.036	0.078	0.008	1	1
rs2299787	21	40212841	0.024	0.019	0.052	1	1
rs9979680	21	41419328	0.036	0.056	0.005		
rs10483084	21	42268393	0.031	0.027	0.044	1	

rs4819338	21	44131453	0.045	0.074	0.012	1	1
rs4818919	21	44674292	0.031	0.002	0.042	1	
rs233274	21	44813916	0.061	0.060	0.006	1	
rs9605145	22	15639458	0.039	0.090	0.011	1	1
rs362128	22	16949269	0.038	0.057	0.003		
rs3788317	22	18264379	0.027	0.060	0.008	1	
rs1543779	22	20873046	0.023	0.001	0.036		
rs738803	22	22517433	0.026	0.059	0.010	1	
rs6004279	22	23501862	0.062	0.032	0.001	1	
rs4820615	22	23548216	0.018	0.000	0.033		
rs5752043	22	23740577	0.035	0.078	0.019	1	1
rs8137311	22	24620901	0.033	0.051	0.011		
rs582028	22	25073464	0.024	0.026	0.040		
rs4497966	22	25631384	0.019	0.020	0.038		
rs4822824	22	25662440	0.032	0.066	0.007	1	
rs6519716	22	26220565	0.031	0.008	0.047	1	
rs2207362	22	26237941	0.032	0.052	0.004		
rs2005397	22	26494771	0.070	0.038	0.040	1	1
rs134173	22	26943008	0.023	0.002	0.032		
rs134665	22	27819633	0.024	0.002	0.040		
rs2024120	22	28396498	0.094	0.055	0.001	1	1
rs2267249	22	32391400	0.027	0.060	0.006	1	
rs1009730	22	32585292	0.030	0.000	0.032		
rs10427794	22	33175663	0.059	0.001	0.007	1	
rs5755349	22	33550014	0.022	0.048	0.003		
rs2267347	22	35337453	0.030	0.045	0.042	1	
rs1157557	22	35706174	0.067	0.039	0.014	1	1
rs2284033	22	35858534	0.033	0.066	0.003	1	
rs4821843	22	37665137	0.025	0.042	0.001		
rs926914	22	39742654	0.041	0.012	0.034		
rs137831	22	40228313	0.060	0.013	0.006	1	
rs139228	22	42940334	0.022	0.043	0.034		
rs5765710	22	43234891	0.059	0.079	0.008	1	
rs136564	22	44085411	0.067	0.109	0.000	1	1
rs8138001	22	44106377	0.039	0.073	0.055	1	1
rs139596	22	45545000	0.040	0.003	0.061	1	1
rs131884	22	45758392	0.059	0.002	0.022	1	
rs2027855	22	46158032	0.026	0.005	0.048	1	
rs1157016	22	46573965	0.073	0.039	0.007	1	1
rs6007825	22	47002289	0.023	0.055	0.019		
rs10483243	22	47390986	0.036	0.002	0.066	1	1
rs2337496	22	47500018	0.054	0.044	0.011		
rs9616426	22	47812437	0.039	0.069	0.004	1	1
rs8138283	22	48162340	0.058	0.032	0.007	1	
rs7510868	22	49120188	0.035	0.067	0.005	1	

a. Five population In was determined using CHB, JPT, Korean, Filipino and Vietnamese participants as describe[illegible]
b. The 1.5K EAS-AIMs and 0.75K EAS-AIMs are indicated.

d in text.
